# Cell-type-resolved genetic variation shapes inflammatory bowel disease risk

**DOI:** 10.1038/s41586-026-10627-z

**Published:** 2026-06-03

**Authors:** Tobi Alegbe, Bradley T. Harris, Laura Fachal, Lucia Ramirez-Navarro, Marcus Tutert, Monika Krzak, Mennatallah Ghouraba, Michelle Strickland, Matiss Ozols, Celeste E. Cohen, Saniya Khullar, Eleonora Khabirova, Nikolaos I. Panousis, David Ochoa, Noor Wana, May Xueqi Hu, Jason Skelton, Jasmin Ostermayer, Kimberly Ai Xian Cheam, D. Leland Taylor, Yong Gu, Claire Dawson, Tina Thompson, Kenneth Arestang, Nilanga Nishad, Biljana Brezina, Charry Queen Caballes, Wendy Garri, Steven Leonard, Vivek Iyer, Miles Parkes, Chris Wallace, Rebecca E. McIntyre, Cristina Cotobal Martin, Gareth-Rhys Jones, Tim Raine, Carl A. Anderson

**Affiliations:** 1 Wellcome Sanger Institute, Hinxton, UK; 2 Open Targets, Hinxton, UK; 3 EMBL-EBI, Hinxton, UK; 4 GlaxoSmithKline, Stevenage, UK; 5 Addenbrooke’s Hospital, Cambridge, UK; 6 Department of Medicine, University of Cambridge, Cambridge, UK; 7 MRC Biostatistics Unit, University of Cambridge, Cambridge, UK; 8 University of Edinburgh Centre for Inflammation Research, Queens Medical Research institute, Edinburgh, UK

**Keywords:** Gene expression, Quantitative trait loci, Inflammatory bowel disease, Genome-wide association studies

## Abstract

Most genetic variants associated with complex diseases lie in non-coding regions^1^, complicating efforts to identify effector genes and relevant cell types. Here we map *cis*-expression quantitative trait loci (eQTLs) across 2.2 million single cells using intestinal biopsies and blood from 421 individuals, including 125 with inflammatory bowel disease (IBD). Cell-type-level eQTLs were more distal to transcription start sites, enriched in enhancers, less likely to regulate the nearest gene, and more than 3.5-fold more likely to colocalize with IBD loci detected in genome-wide association studies (GWASs) than eQTLs detected at tissue-level resolution. We nominate effector genes at more than half of known IBD loci, including *MAML2*, *PSEN2* and *ZMIZ1* in myeloid cells, implicating reduced Notch signalling in intestinal immune dysfunction. We also identify Wnt-regulated genes, including *MYC*, in epithelial stem and progenitor cells, suggesting that impaired renewal contributes to barrier breakdown. Our results provide a mechanistic map that links genetic risk to specific genes and cell types in IBD, and a generalized framework for interpretation of GWAS loci using single-cell eQTL mapping of disease-relevant tissues in complex diseases.

## Abstract (web summary)

Single-cell mapping of *cis*-expression quantitative trait loci in inflammatory bowel disease revealed distal, enhancer-enriched variants detected at the cell-type level more frequently co-localize with genome-wide association study loci than those identified at the tissue level.

## Main

Common complex diseases cause substantial morbidity and mortality worldwide, yet the biological mechanisms that underlie susceptibility remain incompletely understood. Genetic variation contributes considerably to disease susceptibility, and GWASs have identified hundreds of thousands of associations between genetic variants and complex diseases and traits^2^. However, more than 90% of GWAS signals map to non-coding regions of the genome^1^, complicating efforts to identify dysregulated genes, pathways and cell types.

Non-coding variants are thought to influence disease risk by altering expression of nearby genes. This has prompted large-scale efforts to map *cis*-eQTLs and integrate them with GWAS findings. Although these studies have advanced our understanding of disease biology^3–7^, most GWAS loci remain unannotated. This lack of functional resolution poses a major challenge for therapeutic development, as drug targets supported by human genetic evidence are substantially more likely to progress successfully through clinical trials^8–11^. One possible explanation for this annotation gap is that eQTL and GWAS signals often have different genomic contexts: eQTLs are more frequently found in promoters, whereas GWAS signals are enriched in enhancers^12^. However, this divergence may reflect methodological biases in current eQTL discovery efforts, which often rely on low-resolution bulk RNA sequencing of heterogeneous tissues or coarsely sorted cell populations^13–16^. These approaches may favour detection of regulatory effects that are shared across multiple cell types, which are more likely to reside in promoters than enhancers^17^.

Single-cell RNA sequencing (scRNA-seq) can capture gene expression variation at much higher cellular resolutions, enabling the identification of cell-type-specific eQTLs that may be inaccessible to bulk tissue studies^7,18–23^. However, it remains unclear to what extent eQTLs identified at different cellular resolutions improve effector gene nomination at GWAS loci.

We hypothesized that GWAS signals would be preferentially enriched among cell-type-specific single-cell eQTLs (sc-eQTLs) as contextual restriction of their regulatory effects may mean they face weaker selective pressures. To test this, we established the IBDverse project, generating large-scale scRNA-seq datasets from blood and intestinal biopsies of the terminal ileum and rectum—the sites that are most commonly affected in Crohn’s disease (CD) and ulcerative colitis (UC), respectively. These diseases represent the two main forms of IBD, a debilitating group of gastrointestinal disorders affecting more than 4.9 million people worldwide. Using nearly 2.2 million single-cell transcriptomes from more than 400 individuals, including 125 with IBD, we comprehensively mapped eQTL effects across a broad range of cellular resolutions, from individual cell types to tissue-level groupings. Our analyses enabled the nomination of candidate effector genes and cell types for more than half of the 321 known IBD loci, including 74 loci where an effector gene is nominated for the first time. Furthermore, we systematically quantified the contribution of eQTLs detected at different cellular resolutions to GWAS association signals, demonstrating that cell-type-level eQTLs are substantially enriched among IBD GWAS loci and preferentially reside in distinct genomic contexts compared to eQTLs detected across broader cell groupings. These findings highlight the power of single-cell resolution for interpreting complex disease genetics and provide a general framework for effector gene discovery across diverse diseases.

## A cellular atlas of intestine and blood

**Fig. 1:**
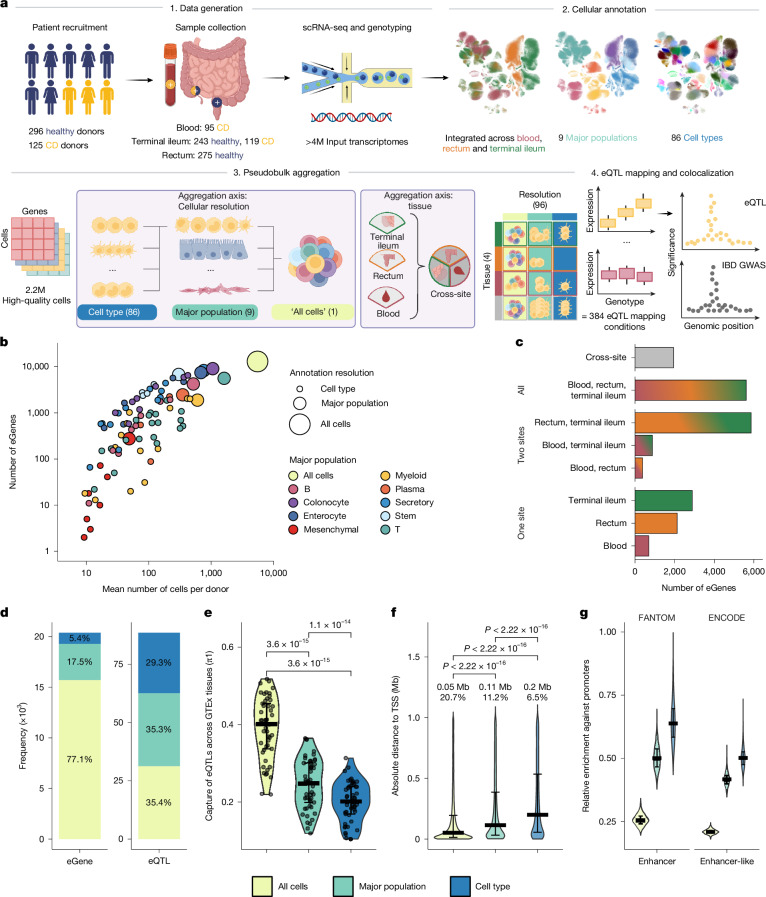
Distinct eQTLs are observed at different cellular resolutions. **a**, scRNA-seq data were generated from blood, rectal and terminal ileal biopsies from 421 healthy individuals and patients with CD. After joint quality control, integration and clustering, a multi-site single-cell atlas of IBD was constructed (Methods and Supplementary Fig. 1). Genome-wide germline genotypes were obtained from the same individuals. Within each anatomical site, eQTLs were mapped at three resolutions: cell type (highest resolution; *n* = 86); major population (coarse resolution; *n* = 9); and the entire sample (‘all cells’; lowest resolution; *n* = 1). Samples were also combined across sites (cross-site), generating 4 site-level annotations, 96 cell-level annotations, and up to 384 unique groupings. Disease effector genes were nominated by colocalizing eQTLs with IBD GWAS signals. Panel created in BioRender; Harris, B. https://BioRender.com/61lbk6w (2026). **b**, Relationship between the mean number of cells per donor and the number of eGenes detected per cell type in the cross-site analysis. **c**, Sharing of eGenes across anatomical sites. **d**, Identification of eGenes (left) and eQTLs (right) across resolutions. Genes and eQTLs are grouped by the minimum resolution at which they were detected, with percentages shown for each level. **e**, Capture of resolution-specific eQTLs in 49 GTEx tissues using the π1 statistic (higher values indicate greater sharing; Methods). Each point represents comparison with one GTEx tissue. *P* values were derived from paired Wilcoxon tests. **f**, Distance between each eQTL lead variant and the TSS of its eGene. Median distances and the percentage located less than 10 kb from the TSS are shown above the plots. Total number of eQTLs is 31,307 (‘all cells’), 31,261 (major population) and 25,980 (cell type). **g**, Relative enrichment of eQTLs from each resolution in FANTOM and ENCODE enhancer annotations versus promoters, estimated from 1,000 bootstraps against matched variants (Methods). All pairwise two-sided *t*-tests had *P* < 2.2 × 10^−16^. Horizontal bars in **e**–**g** indicate the 25th, 50th and 75th percentiles.

**Extended Data Fig. 1:**
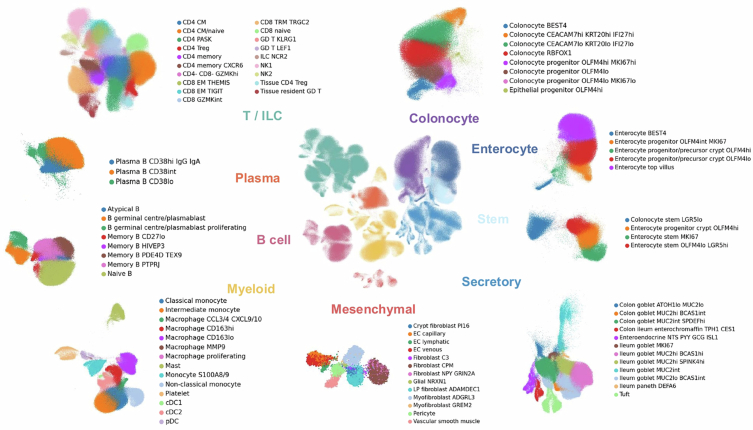
Cell types identified in the IBDverse dataset. Uniform Manifold Approximation and Projection (UMAP) of the 9 major populations and 86 cell types identified after quality control and clustering.

**Extended Data Fig. 2:**
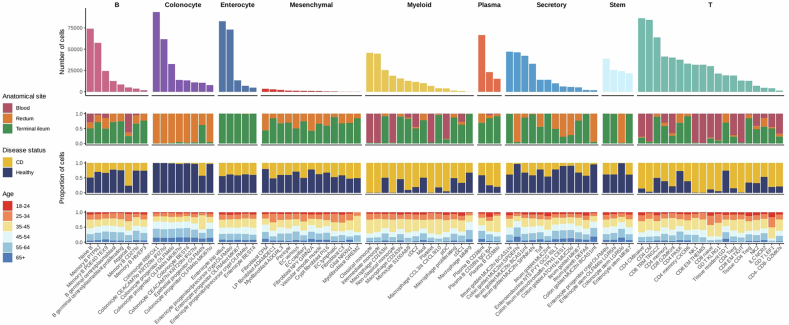
Cell type-level metadata. Total number (top) and contribution of different sites, disease status, and ages of sample to each cell-type in the 1,852,681 cells in the atlasing cohort.

To enable large-scale sc-eQTL mapping at sites relevant to IBD, we created IBDverse, comprising scRNA-seq data generated from 275 healthy rectal biopsies, 119 CD terminal ileal biopsies, 243 healthy terminal ileal biopsies and 95 blood samples from individuals with CD (Fig. 1a). After stringent quality control, we obtained more than 1.8 million high-quality cells, which we clustered into 86 transcriptionally distinct groups across 9 major cell populations (Supplementary Fig. 1 and Extended Data Fig. 1). Samples from all anatomical sites were analysed jointly to ensure consistent cell-type assignment and to capture both site-specific and shared cell states. Following training and annotation with CellTypist^24^ (Methods), our final sc-eQTL mapping cohort comprised nearly 2.2 million cells from 396 individuals with matched genome-wide genotype data (Extended Data Figs. 1c and 2).

## Cell-type resolution reveals distinct eQTLs

Leveraging the single-cell and multi-site nature of IBDverse, we performed *cis*-eQTL mapping across a broad range of cellular resolutions. Specifically, we mapped eQTLs at the level of 86 transcriptionally defined cell types, within 9 major cell populations, and across all cells combined from each sample (‘all cells’ resolution). This was done within each anatomical site separately (rectum, terminal ileum or blood), or by combining expression across sites (cross-site) (Fig. 1a), resulting in a theoretical maximum of 384 unique cellular annotations. Where eQTL effects were detected, we mapped conditionally independent signals and grouped lead variants based on linkage disequilibrium to enable direct comparison of eQTL effects across annotations and resolutions (Methods).

**Extended Data Fig. 3:**
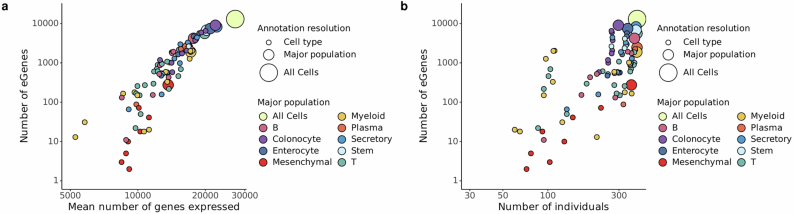
Factors influencing eGene detection. Association between the number of eGenes detected in the cross-site analysis and (a) the mean number of genes per pseudobulked sample or (b) the number of individuals.

We identified 88,548 eQTLs associated with the expression levels of 20,389 out of the 24,761 genes tested (82.2%) (genome-wide false discovery rate (FDR) < 0.05; Methods). Because the germline remains fixed from conception, this represents genetic variation that confers a regulatory effect on gene expression level. In total, 252 out of the possible 384 cellular annotations had a sufficient number of samples for testing and at least 1 eQTL was detected in 251 out of these 252 annotations (Methods), with the number of eGenes (genes with at least 1 significant eQTL) being positively correlated with the number of constituent cells, the number of donors, and the average number of genes expressed per cell (all *P* < 0.001; Fig. 1b and Extended Data Fig. 3). Most eGenes were shared across sites, most frequently between the rectum and terminal ileum (5,861, 28.7%), followed by all three sites (5,613, 27.5%) (Fig. 1c). However, many eGenes (1,945, 9.5%) were only detected when combining sites, displaying the value of aggregating common cell types across different tissues.

**Extended Data Fig. 4:**
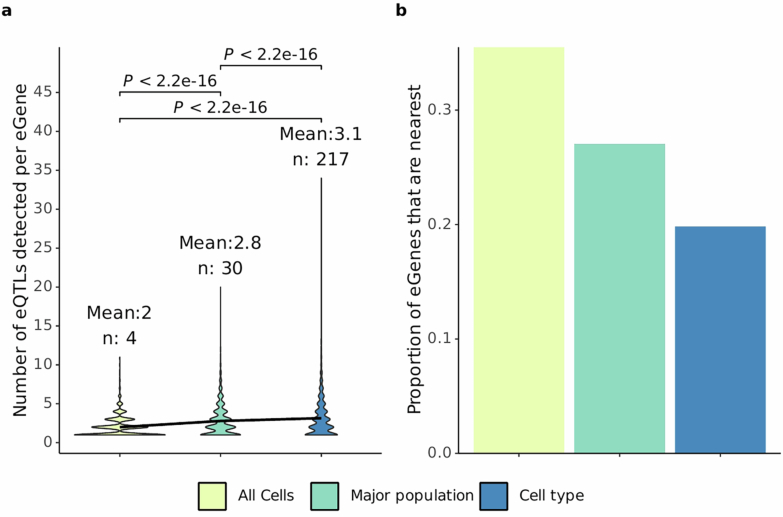
Number of eQTLs per eGene and likelihood of targeting the nearest gene across resolutions. (a) Comparison of the number of eQTLs (determined by linkage disequilibrium clumping, see Methods) that were found for each eGene at each resolution. Black line connects the means of each resolution, ‘n = ’ indicates the total number of annotations per resolution, p-values calculated by Wilcoxon test. (b) Proportion of eQTLs detected at each resolution for which the nearest gene is the eGene.

The majority of eGenes (15,713, 77.1%) were detected at the ‘all cells’ resolution, with additional eGenes identified at the major population (3,565, 17.5%) and cell-type levels (1,111, 5.4%) (Fig. 1d). By contrast, nearly one-third (25,980, 29.3%) of eQTLs were detected at only the cell-type level, with even more detected at the major population but not ‘all cells’ level (31,261, 35.3%). The number of eQTLs per eGene also declined at coarser resolutions—averaging 3.1 at the cell-type level, 2.8 at the major population level and 2.0 at the ‘all cells’ level (all comparisons *P* < 2.2 × 10^−16^; Extended Data Fig. 4a). Although many of our eQTLs are also captured by bulk eQTLs from GTEx^4^ or peripheral blood sc-eQTLs from Yazar et al.^18^ (Methods and Supplementary Note), our cell-type eQTLs were much less likely to be found previously than major population or ‘all cells’ eQTLs (median π1 with GTEx tissues = 0.2, 0.25 and 0.4, respectively, maximum *P* value = 1.1 × 10^−14^) (Fig. 1e). Together, these findings demonstrate a key distinction: although the ‘all cells’ resolution identifies more eGenes overall, cell-type-level mapping reveals distinct and diverse regulatory variants that are masked in bulk analyses.

Given these differences in detection, we next tested whether the genomic location of eQTLs also varied by resolution. eQTLs detected only at the cell-type level were located significantly further from the transcription start site (TSS) of their associated eGene (median distance = 200 kb) than those detected at the major population (median = 114 kb) or ‘all cells’ level (median = 52 kb; both comparisons *P* < 2.2 × 10^−16^; Fig. 1f). Correspondingly, the nearest gene was the eGene for 35.5% of eQTLs detected at the ‘all cells’ level, compared to 27% and 19.8% for those at the major population and cell-type levels, respectively (Extended Data Fig. 4b).

Functionally, eQTLs identified at only the cell-type level were more enriched in FANTOM and ENCODE enhancer annotations rather than promoters, compared with those at the ‘all cells’ or major population level (*P* < 2.2 × 10^−16^ for all comparisons) (Fig. 1g). Because GWAS signals also tend to be found in enhancers^12^, these findings suggest that gene expression changes driven by eQTL signals found only at a cell-type resolution are more likely to mediate phenotypic differences in complex traits and diseases.

**Extended Data Fig. 5:**
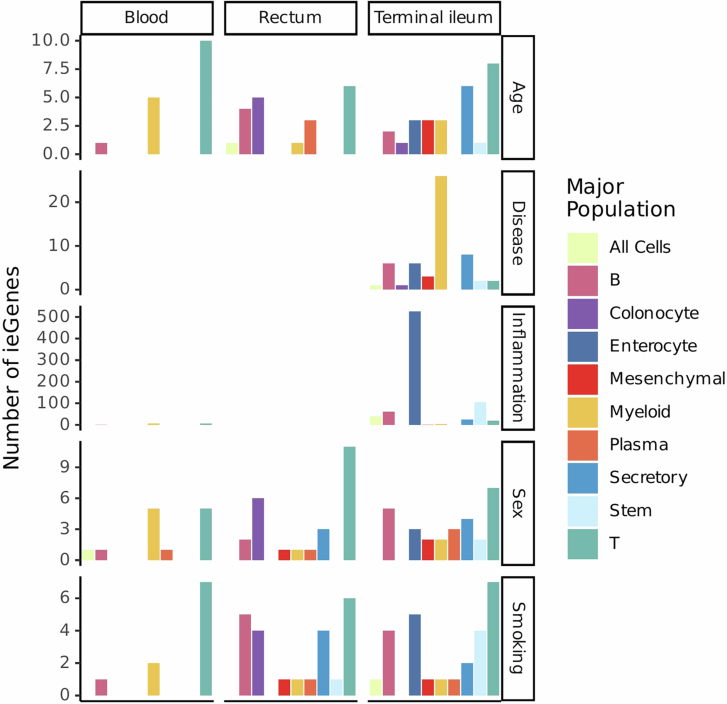
Number of ieGenes detected for each variable. Absolute number of eGenes detected by interaction (ieGenes) with sample level phenotypes, grouped by the interaction variable and the major population in which ieGenes were detected.

We next explored whether genetic effects on gene expression are modulated by individual-level traits. We performed interaction eQTL (ieQTL) mapping using genotype–expression models that included interaction terms for age, sex and smoking status as well as CD diagnosis and macroscopic degree of ileal inflammation at the time of sampling, where possible (Methods). This analysis could uncover effects that are exacerbated by, specific to, or diminished during disease onset or progression. We identified 1,051 ieQTLs regulating the expression of 977 genes (ieGenes), and 764 (78.2%) of these ieGenes were modulated by gut inflammation status (adjusted *P* value < 0.05; Methods). Of note, the majority of these inflammation-responsive ieGenes (501, 65.6%) were detected in enterocytes, suggesting that genetic effects on gene expression in this major population are highly sensitive to inflammatory context (Extended Data Fig. 5).

Although most ieGenes (941, 96.3%) were also eGenes, only 53 ieQTLs (5.0%) were in strong linkage disequilibrium with eQTLs (*r*^2^ > 0.5) for the same gene. Even at a much lower threshold of *r*^2^ > 0.1, only 149 (14.3%) of ieQTLs overlapped with eQTLs (Supplementary Fig. 2), indicating that these effects are largely distinct from those observed in conventional eQTL analyses. To better understand the pathways disrupted by the interaction between inflammation and genetics in enterocytes we performed gene set enrichment analysis, which returned four pathways (coefficient > 0; FDR < 0.05; Supplementary Information). Notably, the most enriched pathway was ‘response to metal ion binding’ in enterocytes (coefficient = 2.04). Leading edge genes encode many isoforms of metallothionein proteins that bind metal ions^25^, such as *MT2A*, *MT1H* and *MT3*, genes that are also associated with protective effects against inflammation across several tissues including the skin^26^, blood^27^, liver^28^, lung^29^ and intestine^30,31^. Together, these findings support the value of mapping genetic regulation under different disease-relevant conditions to uncover context-dependent mechanisms that would be missed in steady-state or healthy tissue analyses.

## Cell-type eQTLs better nominate disease effector genes

Next, we reasoned that cell-type-specific sc-eQTLs, being more distal to TSSs and enriched in enhancer elements, would be more likely to colocalize with GWAS signals than eQTLs identified at coarser resolutions. We therefore performed colocalization analyses between IBD GWAS signals and our full set of eQTL and ieQTL maps, using summary statistics for CD, UC and the more general phenotype of IBD^3^. This method tests the likelihood that two traits, in this case eQTL and GWAS associations, share a causal variant at a given genetic locus^32^. To benchmark our findings, we compared them to colocalizations that were previously reported for IBD loci in the Open Targets genetics portal, including those based on expression, splicing and other molecular QTLs^33^.

**Fig. 2:**
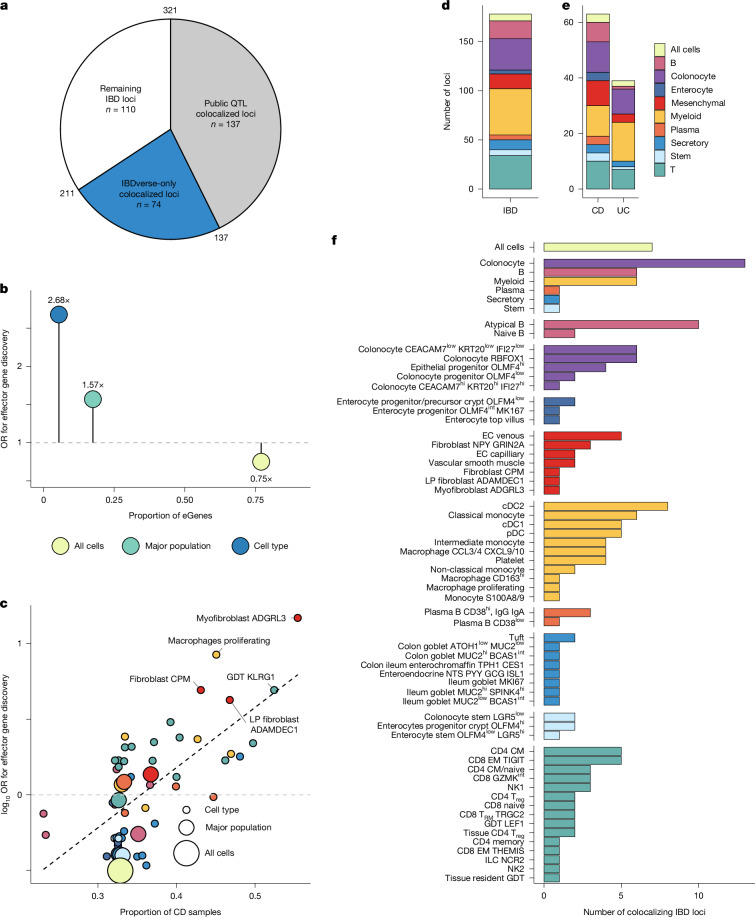
Detection and distribution of colocalization events with IBD, CD and UC GWAS. **a**, The number of IBD GWAS loci^34^ coloured by the proportion that previously colocalized (posterior probability of hypothesis 4 (PP_h4_) > 0.75) with existing bulk quantitative trait loci (QTLs) in Open Targets genetics (Methods) or do so for the first time with IBDverse sc-eQTLs. The total number of loci (321) is shown on top, along with the cumulative number of loci for which disease effector genes are nominated by each dataset. **b**, Comparison between the proportion of eGenes detected and the OR between disease effector gene and eGene detection. Values greater than one indicate an enrichment of disease effector genes among eGenes; values less than one indicate underrepresentation of disease effector genes among eGenes. **c**, Relationship between the proportion of CD samples for each cellular annotation of the terminal ileum and log_10_-transformed OR between disease effector gene and eGene detection. The horizontal line indicates an OR of 1 and therefore no enrichment, as in **b**. Univariate linear regression slope = 3.98, *P* = 4.68 × 10^−9^. Colour scheme as in **d**,**e**. **d**,**e**, The number of IBD (**d**) or subtype-specific (**e**) GWAS loci that colocalize with IBDverse eQTLs (PP_h4_ > 0.75), coloured by the major population (or ‘all cells’) where the variance in disease effector gene expression explained by the colocalizing variant was greatest. **f**, The number of loci where variance in disease effector gene expression explained by the colocalizing variant was greatest across cell types. Bars for each annotation are coloured by the major population they belong to. Colour scheme as in **d**,**e**. CRM, central resident memory; EC, endothelial cell; EM, effector memory; GD, gamma-delta; LP, lamina propria; NK1 and NK2, natural killer cell subsets 1 and 2; T_reg_, regulatory T cell; T_RM_, tissue resident memory T cell.

Among the 321 IBD loci reported to date^34^, we observed colocalization (posterior probability of a shared causal variant (PP_h4_) > 0.75) with either eQTLs or ieQTLs at 180 loci (56%), implicating 419 distinct effector genes (Extended Data Table 1 and Supplementary Information). This includes 74 loci where no effector gene had previously been nominated by colocalization in the Open Targets genetics portal (Fig. 2a) and 257 novel disease effector genes (101 at newly colocalized loci). Even compared with an sc-eQTL study of peripheral blood mononuclear cells from 1,925 healthy individuals^35^, our study nominates 173 novel effector genes for IBD, including 88 across 49 loci where one is not otherwise nominated (PP_h4_ > 0.8), leaving 169 (67.3%) out of the 251 effector genes that Cuomo et al.^35^ nominate to be specific to their dataset. Despite a considerably smaller sample size, 18 loci have effector genes nominated only in our gastrointestinal immune populations. The likelihood that we identify a novel disease effector gene compared with this dataset was negatively associated with the number of cells within each major population that was derived from the blood (slope = −0.194, *P* = 0.014) (Supplementary Fig. 3), indicating a considerable benefit to the inclusion of samples from the primary site of disease. Notably, at 2 of these 180 loci, colocalization was observed only with ieQTLs, illustrating that context-dependent regulatory effects can reveal additional candidate effector genes that are not detected under baseline conditions.

Our eQTLs and ieQTLs also colocalized with 104 out of the 137 (75.9%) GWAS loci that were already reported to colocalize with molecular QTLs in the Open Targets genetics portal (Supplementary Information). At 78 of these loci, at least 1 effector gene colocalized in both our data and the Open Targets analysis. Notably, 103 of the previously implicated genes had only been supported by colocalization in non-gut tissues, whereas our data revealed colocalization in gut tissue for the first time. Together, these findings significantly extend effector gene nomination at IBD loci and enable more targeted hypotheses about the cellular contexts in which genetic risk of IBD is exerted.

We next tested whether eGenes identified at the cell-type, major population or tissue level differed in their likelihood of colocalizing with IBD GWAS loci. GWAS signals were significantly more likely to colocalize with eQTLs identified at the cell-type level (odds ratio (OR) between eGene detection and disease effector gene nomination = 2.68), with more modest enrichment for major population-level eQTLs (OR = 1.57) and negative enrichment for those identified at the ‘all cells’ level (OR = 0.75) (Fig. 2b). This pattern was not explained by cell-type-specific expression of genes at these loci, as expression specificity did not differ between eGenes that colocalized at different resolutions (minimum Wilcoxon *P* value = 0.4; Supplementary Fig. 4). These findings support the hypothesis that disease-relevant regulatory variation is often active in restricted cellular contexts, and that mapping eQTLs at cell-type resolution increases power to detect the effector genes underlying GWAS associations.

To determine whether the inclusion of diseased samples aided disease effector gene discovery, we compared the proportion of CD samples that contributed to eQTL mapping in each cell type of the terminal ileum and the likelihood that an eGene detected in this cell type was a disease effector gene (Fig. 2c). Broadly, this shows that cell types enriched in CD samples are more likely to carry regulatory effects that colocalize with disease (univariate linear model slope = 3.98, *P* = 4.68 × 10^−9^). The greatest enrichment of disease effector genes was observed in *ADGRL3*^+^ myofibroblasts, proliferating macrophages and *KLRG1*^+^ γδ T cells, each of which have established roles in IBD susceptibility^36–40^. Together, this suggests that the inclusion of CD samples has a general positive effect on the discovery of disease effector genes.

To refine hypotheses for the annotations in which colocalized effects may be active, we identified, for each colocalized signal, the annotation in which the lead eQTL variant explained the greatest proportion of variance in gene expression (Methods), and refer to these as effector cell types. This approach mitigates power-related biases across eQTL maps that would affect interpretation if based solely on the cell type with the highest posterior probability of colocalization. By focusing on variance explained rather than detectability, we aimed to better prioritize cellular contexts that are likely to mediate disease risk. Across all IBD loci with colocalizing eQTLs, myeloid cell populations were most frequently the cell type in which the lead variant explained the largest proportion of gene expression variance (47 loci, 26.4%), followed by T cells and innate lymphoid cells (T/ILC cells) (34 loci, 19.1%) and colonocytes (32 loci, 18%) (Fig. 2d and Supplementary Information). Broadly, enrichment in these three major populations held when stratifying by disease subtype; at CD-specific loci, the most likely effector cell types were myeloid cells and colonocytes (11 loci each, 17.5%) and T/ILC cells (10 loci, 15.9%), whereas in UC, they were most often myeloid cells (14 loci, 36%) and colonocytes (9 loci, 23.1%) (Fig. 2e). The variance in gene expression explained by the colocalizing variant was correlated with overall expression level for only 103 effector genes (27.2%, *P* < 0.05), indicating that expression level can sometimes contribute, but is not the predominant driver of discovery.

## Dendritic cell Notch signalling in IBD

Among myeloid cell types, dendritic cells (type 2 conventional dendritic cells (cDC2s), type 1 conventional dendritic cells (cDC1s) and plasmacytoid dendritic cells (pDCs)), were three of the top four most frequently nominated effector cell types, with a total of 18 IBD loci showing the highest proportion of gene expression variance explained in these populations (Fig. 2f). In humans, conventional dendritic cells (cDCs) originate from CD34^+^ bone marrow progenitors and differentiate into transcriptionally and functionally distinct subsets, including cDC1 and cDC2. Although both contribute to antigen presentation, they differ in pathogen sensing, cytokine production and T cell priming, particularly in mucosal tissues. These functions are unique to cDCs and are essential for oral tolerance and T cell priming, placing them at a critical interface between innate and adaptive immunity in the gut. Their recurrent nomination as likely effector cell types at IBD loci suggests a central role for dendritic cell-intrinsic pathways in shaping disease risk.

**Fig. 3:**
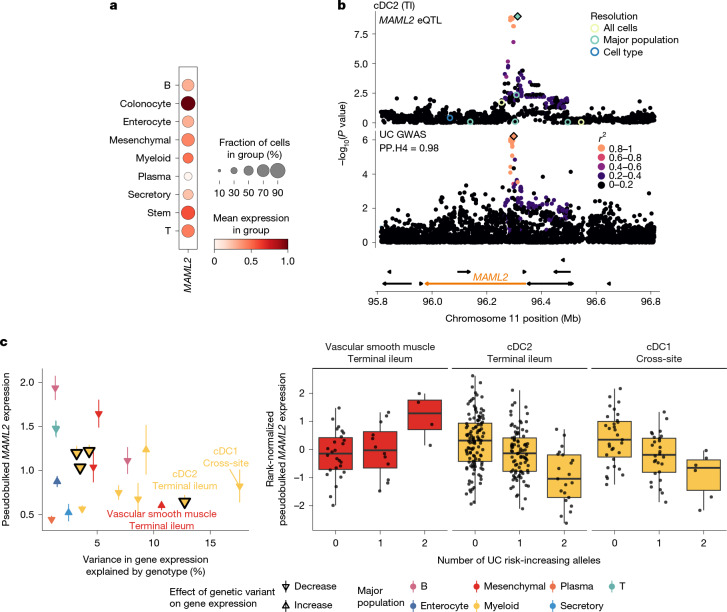
Novel colocalization between *MAML2* dysregulation and IBD highlights susceptibility-associated dysregulation of Notch signalling in conventional dendritic cells. **a**, Expression of *MAML2* (log(1 + counts per 10,000)) across all major populations. **b**, Significance (−log_10_(*P* value)) of variant associations with *MAML2* expression and UC susceptibility. Lead variants for each dataset are presented as a diamond. The lead variant for the eQTL is coloured by the minimum resolution at which the eQTL is detected. All other variants are coloured by their in-sample linkage disequilibrium (*r*^2^) with the index variant from each dataset. Lead variants for other eQTLs are outlined by the colour of the minimum resolution at which they were detected. TI, terminal ileum. **c**, Left, median expression of *MAML2* and the amount of variance in normalized *MAML2* expression explained by genotype at the colocalizing variant for each annotation with a nominal eQTL effect (*P* < 0.05) and median expression greater than zero. Annotations where colocalization is detected (PP_h4_ > 0.75) are outlined. Right, inverse normal transformed expression levels of *MAML2* in the three annotations where the greatest variance in gene expression is explained by this variant. Box plots indicate the 25th, 50th and 75th percentiles and whiskers extend to 1.5 times the interquartile range.

Out of the 13 IBD loci where the strongest colocalizing regulatory effects were observed in cDCs, disease effector genes at 2 are associated with Notch signalling. Whereas previously nominated effector genes by Open Targets genetics and Cuomo et al.^35^ include only 4 members of this pathway, we nominate 6 novel effector genes that encode Notch regulators, making it over-represented among our disease effector genes (*P* = 0.026). One of these is *MAML2*, which is widely expressed across major populations (Fig. 3a) and encodes a nuclear co-activator essential for canonical Notch pathway activation^41,42^. In our data, a *MAML2* eQTL colocalized with a UC GWAS signal in cDC2s from the terminal ileum (PP_h4_ = 0.98), and was the only gene supported at that locus (Fig. 3b). This represents a novel colocalization that is not reported in the Open Targets genetics portal. Although we identified 8 distinct eQTLs for *MAML2* across cell types and tissues (Supplementary Fig. 5), colocalization with UC was observed for a single eQTL effect that was in low linkage disequilibrium with the others (maximum *r*^2^ = 0.3), suggesting that the disease association arises from a distinct regulatory effect that is active in dendritic cells.

Although colocalization was detected in cDC2s, the lead variant also explained substantial variance in *MAML2* expression in cDC1s (Fig. 3c). We interpret this as probably reflecting reduced power to detect colocalization in cDC1s, which are both rarer in intestinal tissue and less well represented in our dataset (Extended Data Fig. 2). As eQTL effect sizes were not correlated with *MAML2* expression (Spearman’s *R* = –0.29; *P* = 0.21), the disease association is likely driven by a cell-type-specific regulatory effect, not by general gene expression levels. Together, these results support the conclusion that dysregulation of *MAML2* expression by this specific sc-eQTL in cDCs contributes to UC risk.

**Extended Data Fig. 6:**
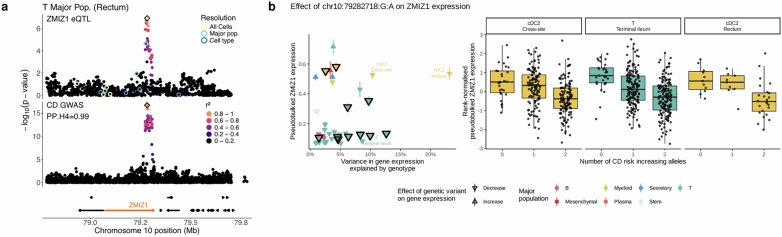
Identification and contextualisation of NOTCH signalling colocalization target *ZMIZ1.* (a) Regional association pattern between genetic dysregulation of *ZMIZ1* and CD susceptibility. Point fill and colour key follows that of 4b. (b) Comparison of the variance of disease effector gene expression explained by the colocalizing variant across conditions where a nominal (p < 0.05) eQTL effect was detected (left), and boxplots showing effect in the three annotations with the most variance explained (right). Boxplots indicate the 25th, 50th and 75th percentiles and whiskers extend to 1.5 times the interquartile range.

An eQTL for an additional Notch pathway gene, *ZMIZ1*, also colocalized with IBD GWAS signals in dendritic cells (Extended Data Fig. 6a). *ZMIZ1* encodes a transcriptional co-regulator of STAT and Notch pathways, and its deletion in mice phenocopies disruption of Notch ligand activity^43^. In our data, a variant associated with reduced *ZMIZ1* expression colocalized with CD risk (PP_h4_ = 0.99), with the strongest effect observed in cDC2s from the rectum (23% variance explained) (Extended Data Fig. 6b). Although we refer to these cells as cDC2s based on their transcriptional profile, we note that monocyte-derived dendritic cells (mo-DCs) are indistinguishable from cDC2s in our atlas. Together with findings at *MAML2*, this result suggests that disruption of Notch pathway activity across dendritic cell subsets may have a protective role in maintaining intestinal homeostasis potentially by promoting tolerance or limiting pro-inflammatory signalling at the mucosal surface.

## Epithelial Wnt signalling in IBD risk

**Extended Data Fig. 7:**
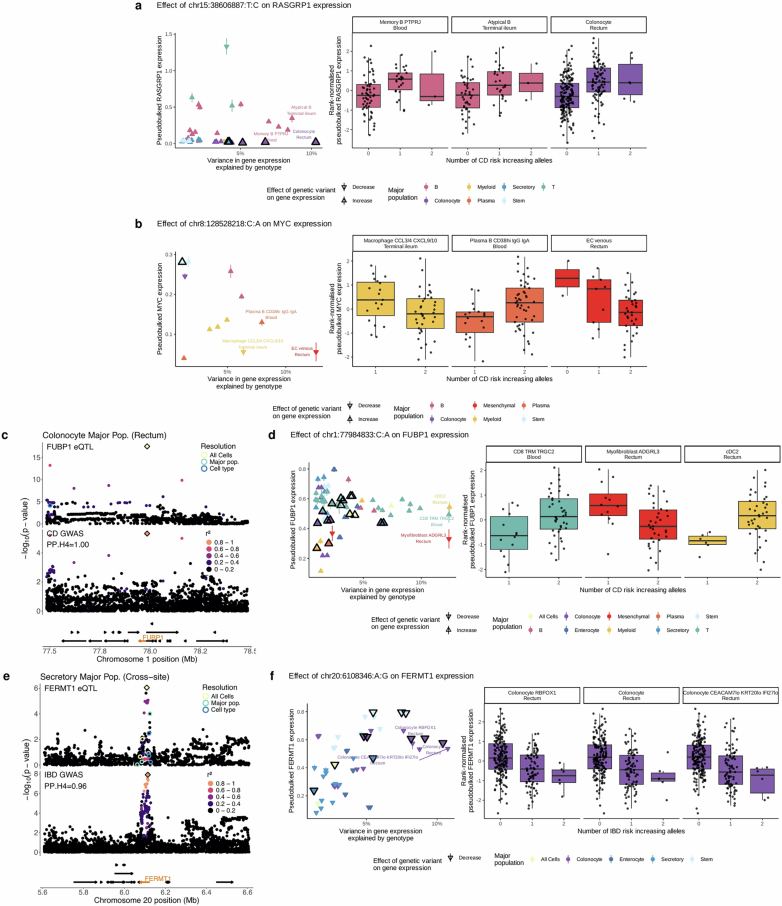
IBD colocalizations may impact epithelial barrier function and Wnt signalling. (a/b/d/f) Comparison of the variance of disease effector gene expression explained by the colocalizing variant across conditions where a nominal (p < 0.05) eQTL effect was detected for each gene (left), and boxplots showing effect in the three annotations with the most variance explained (right). (c/e) Regional association pattern between genetic dysregulation of *FUBP1* and CD susceptibility or *FERMT1* and IBD susceptibility, respectively. Point fill and colour key follows that of 4b. Boxplots indicate the 25th, 50th and 75th percentiles and whiskers extend to 1.5 times the interquartile range.

Next, we examined colocalizations that implicate epithelial cells, where barrier dysfunction represents an established mechanism of IBD susceptibility. Colonocyte populations were among the most frequently nominated effector cell types, particularly the *RBFOX1*-expressing and *CEACAM7*,* KRT20* and* IFI27-*expressing subsets (6 loci each), along with the broader colonocyte compartment at the major population level (13 loci) (Fig. 2f). At one such locus, we replicate a previously reported colocalization between IBD risk and an eQTL associated with reduced expression of *FERMT1*^44–46^ (PP_h4_ = 0.96; Extended Data Fig. 7e,f). Null variants in *FERMT1* are the most common defect in Kindler syndrome, a rare condition of epithelial fragility caused by lack of FERMT1 (also known as kindlin-1), which anchors the actin cytoskeleton to the plasma membrane, and is an important cause of monogenic IBD^47–49^. Our single-cell analysis refines this colocalization to epithelial populations, and *CEACAM7*,* KRT20* and* IFI27-*expressing colonocytes in particular (Supplementary Information).

**Fig. 4:**
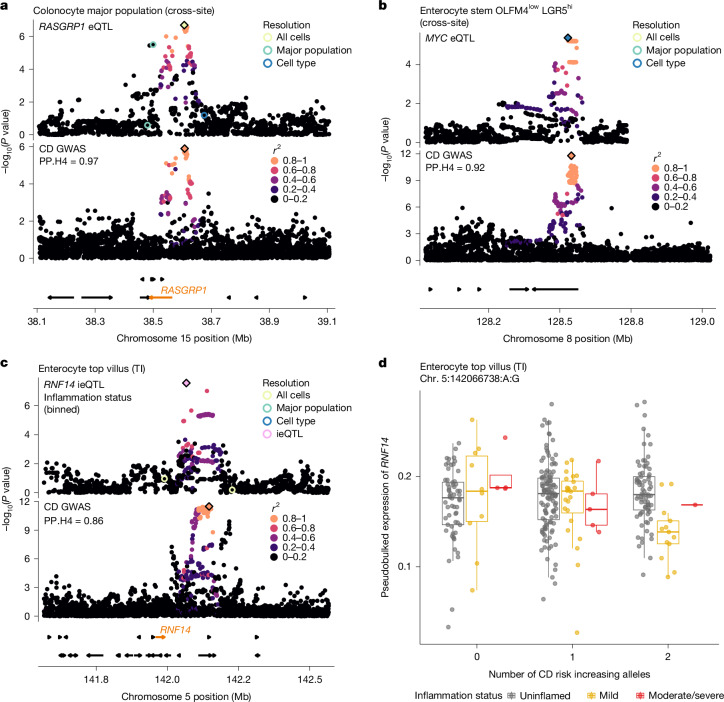
Wnt-associated genes *RASGRP1*, *MYC* and *RNF14 *colocalize with IBD susceptibility loci. **a**–**c**, Significance (–log_10_(*P* value)) of variant associations with *RASGRP1* (**a**), *MYC* (**b**) and *RNF14* (**c**) expression and CD susceptibility. Lead variants for each dataset are presented as a diamond. The lead variant for the eQTL is coloured by the minimum resolution at which the eQTL is detected. All other variants are coloured by their in-sample linkage disequilibrium (*r*^2^) with the index variant from each dataset. Lead variants for other eQTLs are outlined by the colour of the minimum resolution at which they were detected. **d**, The interaction between genotype at the colocalizing variant for *RNF14* expression in enterocytes from the top of the villus and disease severity. Box plots indicate the 25th, 50th and 75th percentiles and whiskers extend to 1.5 times the interquartile range.

Several novel colocalization events also implicated genes involved in epithelial differentiation and renewal. An eQTL for *RASGRP1* colocalized with CD risk (PP_h4_ = 0.97), and the risk allele was associated with increased *RASGRP1* expression in colonocytes (Fig. 4a and Extended Data Fig. 7a). *RASGRP1* encodes a Ras guanine nucleotide exchange factor that modulates MAPK signalling and epithelial proliferation^50,51^, and its depletion results in colitis phenotypes in mice^52^, suggesting a role in maintaining epithelial turnover. In addition, an eQTL for *LPIN3* colocalized with IBD risk (PP_h4_ = 0.89), with the strongest effect observed in *CEACAM7*,* KRT20* and* IFI27-*expressing colonocytes (Supplementary Information). LPIN3 is a phosphatidic acid phosphatase that is involved in lipid metabolism and also regulates Wnt signalling^53^, which is essential for maintaining epithelial stem cell renewal and mucosal integrity. Previously nominated effector genes by Open Targets genetics and Cuomo et al.^35^ include only 6 Wnt regulators, however we nominate an additional 14, making this pathway over-represented among our disease effector genes (*P* = 0.015). Disruption of this pathway can impair epithelial regeneration, promote barrier permeability, and drive intestinal inflammation^54,55^.

Further supporting this mechanism, an eQTL regulating increased *MYC* expression colocalized with CD risk in OLFM4 and LGR5-expressing intestinal stem cells (PP_h4_ = 0.92, Fig. 4b and Extended Data Fig. 7b). *MYC* is a canonical Wnt target and master regulator of epithelial proliferation and metabolism, and its dysregulation perturbs intestinal homeostasis^56–58^. Notably, MYC is a well-established oncogenic transcription factor that drives tumorigenesis through both somatic amplification and germline regulatory variation^59–62^. A role for *MYC* expression in gastrointestinal inflammation is further supported by additional colocalization between CD risk and an eQTL for *FUBP1* (PP_h4_ = 1.00, Extended Data Fig. 7c,d), a regulator of *MYC* and Wnt components such as *DVL1*^63,64^.

An inflammation-dependent ieQTL regulating *RNF14* colocalized with CD susceptibility (PP_h4_ = 0.86, Fig. 4c). *RNF14* encodes ring finger 14 protein, a E3 ubiquitin ligase that enhances Wnt signalling and promotes the expression of downstream targets, including *MYC*^65,66^. In enterocytes from inflamed samples, the CD risk allele was associated with reduced *RNF14* expression (Fig. 4d), supported by fine-mapping (Supplementary Note), and suggests that Wnt signalling and epithelial regeneration may be impaired in the presence of inflammation. However, because our power to detect inflammation-dependent eQTLs outside of enterocytes is limited, we cannot exclude the possibility of larger effects in other cell types.

Together, these findings nominate dysregulation of epithelial renewal and Wnt-dependent proliferation as a potential mechanism contributing to barrier dysfunction in IBD, complementary to established defects in adhesion. Failure to maintain or regenerate the epithelial barrier may be an important and under-recognized contributor to intestinal inflammation.

## Effector gene mapping for drug prioritization

**Extended Data Fig. 8:**
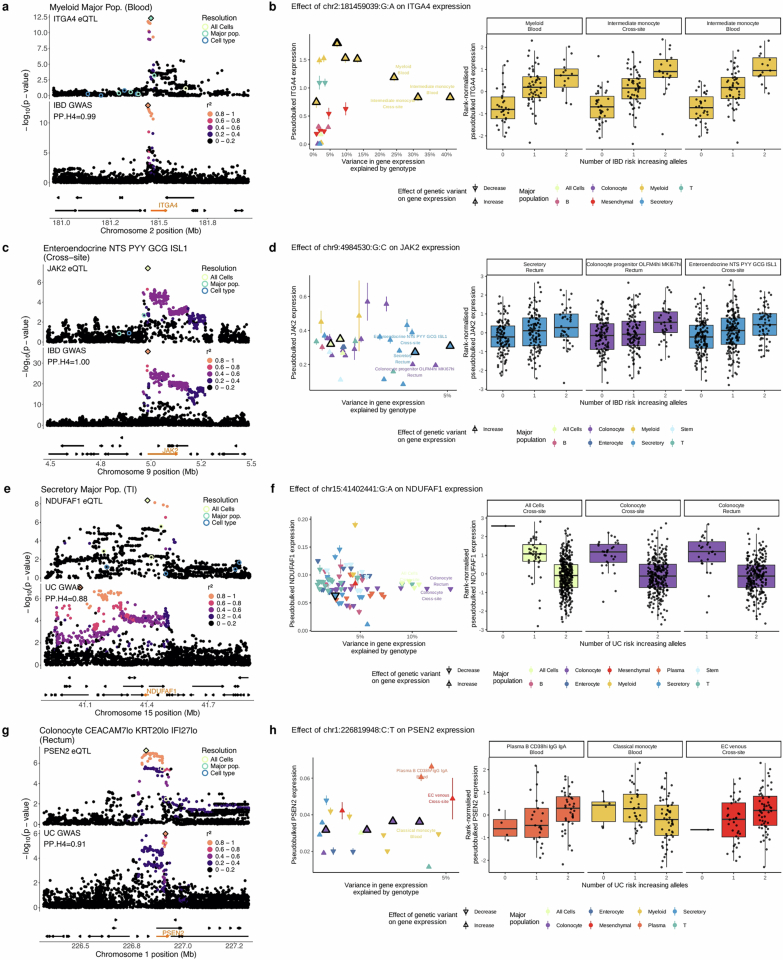
Colocalization of IBD with dysregulation of existing therapeutic targets *ITGA4*, *JAK2*, *NDUFAF1* and *PSEN2.* Regional association pattern between genetic dysregulation of *ITGA4* (a), *JAK2* (c), *NDUFAF1* (e) and *PSEN2* (g) and IBD types. Comparison of the variance of disease effector gene expression explained by the colocalizing variant across conditions where a nominal (p < 0.05) eQTL effect was detected for each gene (b/d/f/h, left), and boxplots showing effect in the three annotations with the most variance explained (right). Boxplots indicate the 25th, 50th and 75th percentiles and whiskers extend to 1.5 times the interquartile range.

To explore how our effector gene nominations might inform therapeutic strategies, we first explored whether any of the colocalizing genes were established or clinically relevant drug targets for IBD. We observed colocalization between IBD risk and eQTLs for *ITGA4* and *JAK2*, which encode direct molecular targets of vedolizumab and tofacitinib, respectively (Extended Data Fig. 8a–d). The strongest colocalizing regulatory effects for these genes indicate their expression is positively associated with IBD susceptibility, consistent with the hypothesis that their inhibition would be therapeutically effective. We also identified colocalizations with *ITGAL*, *TNFSF15* and *TNFRSF14*, genes that, although not directly targeted by existing therapies, participate in immune pathways that are modulated by current IBD drugs. Notably, the strongest regulatory effects for these genes were distributed across diverse tissues and cell types, including myeloid cells from the blood (*TNFSF15*), Plasma B CD38^hi^ IgG IgA from the rectum (*ITGAL*), and intermediate monocytes from the blood (*ITGA4*) (Supplementary Information). These findings are consistent with understanding that IBD treatments target a number of different immunological pathways, many of which are active across multiple cell types and tissues^67^.

**Fig. 5:**
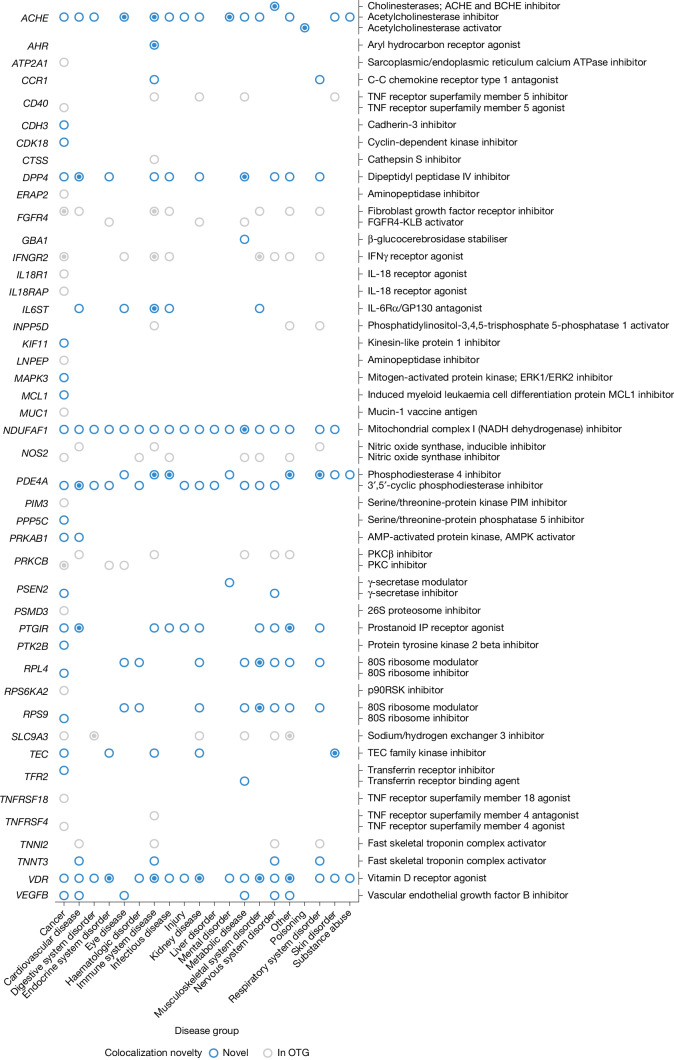
Identifying therapeutic repurposing opportunities by colocalization. Comparison between the disease effector genes identified by colocalization in IBDverse and the maximum stage of clinical trials therapeutic modifiers of each gene are described to be at in the CHEMBL database (Methods). Hollow circles correspond to a maximum trial phase of 0.5–3, and circles with central dot indicate that a drug is approved for use or in trial phase 4. Associations for genes that are previously colocalized in Open Targets genetics (OTG) are represented in grey, genes that colocalize only in IBDverse are dark blue. Drugs are grouped by their broad mechanism of action (*y* axis) and diseases are grouped into related families (*x* axis).

We next investigated whether our colocalized genes included targets of drugs approved or in clinical trials for other diseases, that were potentially suitable for repurposing. Using the ChEMBL database^68^, we identified several such candidates (Fig. 5). We observed colocalization between UC risk and an eQTL for *PRKCB* (PP_h4_ = 0.95; Supplementary Information), which encodes PKCβ, a druggable kinase with inhibitors approved for oncology. In our data, the IBD risk allele was associated with increased *PRKCB* expression in classical and intermediate monocytes, suggesting that increased activity of this kinase may drive pathogenic inflammatory responses. Notably, PRKCB inhibitors are already undergoing early-phase clinical trials for IBD, and our findings provide human genetic support for their mechanism of action in relevant immune cell populations.

We also explored whether our data could offer insights into potential on-target safety concerns. For example, we found colocalization between a UC risk locus and an eQTL for *NDUFAF1* (PP_h4_ = 0.88), which encodes a mitochondrial complex I assembly factor targeted by metformin, which is approved for treatment of diabetes (Extended Data Fig. 8e). The UC risk allele decreases *NDUFAF1* expression across many cell types, but the greatest explained variance in expression was in colonocytes (Extended Data Fig. 8f), which may explain the common metformin side effects of diarrhoea, nausea, weight loss and abdominal discomfort^69,70^. Another notable example was *PSEN2*, where we observed colocalization between an eQTL and UC (PP_h4_ = 0.91; Extended Data Fig. 8g,h). *PSEN2* encodes presenilin 2, a catalytic component of the γ-secretase complex that is required for Notch receptor cleavage and activation. Mutations in *PSEN2* cause autosomal dominant early-onset Alzheimer’s disease by destabilizing fidelity of cleavage by γ-secretase, leading to increased production of aggregation-prone Aβ42 peptides^71,72^. Although γ-secretase inhibitors were developed to reduce this pathogenic cleavage, their clinical use was curtailed by dose-limiting side effects such as weight loss and gastrointestinal bleeding^73,74^. Notably, mouse *Psen2*-knockout models also show susceptibility to colitis^75^, suggesting that *PSEN2* dysregulation may compromise epithelial homeostasis and barrier integrity. Thus, colocalization of eQTLs across diverse single-cell atlases could potentially be used to anticipate context-specific effects of targeted therapies.

## Discussion

IBDverse represents the largest single-cell atlas of IBD-relevant tissues to date, encompassing nearly 2.2 million high-quality single-cell transcriptomes from 421 individuals, including 125 with CD. This size and diversity was critical for mapping eQTLs and nominating probable effector genes and cell types that shape disease susceptibility. By including patients with CD with and without active inflammation, we captured regulatory effects in cell types that expand during disease but are rare in healthy individuals—most notably myeloid subsets^76–79^—which carried more IBD-colocalizing eQTLs than any other major cell population. We also identify 1,051 ieQTLs, including 2 modulated by inflammation, that colocalize with IBD GWAS at loci where an eQTL did not. These context-dependent regulatory effects could only be uncovered by sampling inflamed tissue from individuals with active disease. At the same time, many GWAS-colocalizing eQTLs were identified in cell types abundant in healthy tissue, including epithelial and stromal populations. This underscores the power of generating cell-type-resolved eQTL maps in disease-relevant tissues even in the absence of disease. Given that disease-affected tissues may not be accessible for some complex diseases, our findings reinforce the value of tissue atlases from non-diseased individuals for biological interpretation of GWAS loci, while also demonstrating the added insight gained by integrating samples from individuals with disease and relevant sub-phenotypic data.

A key insight from this study is the critical impact of cell-type resolution on eQTL discovery. Although most eGenes were detected at the ‘all cells’ level (77.1%), many distinct regulatory variants (29.3%) were identified at the cell-type level. Consistent with previous work^12^, eQTLs found at low resolutions were more proximal to TSSs and were relatively enriched in promoters. However, cell-type level eQTLs were distal and more frequently located in enhancer regions—genomic features characteristic of GWAS loci. They were also less likely to regulate the nearest gene, suggesting more distal or context-specific modes of action. Previous studies have shown that cell-type-specific eQTLs tend to be more distal from the TSS^23,80,81^. Here we extend this observation by analysing eQTLs across hierarchical levels of resolution within the same tissue (‘all cells’→Major population→cell type), showing that distance to TSS systematically increases with cellular resolution. In line with these properties, GWAS signals were substantially more likely to colocalize with cell-type-level eQTLs than those detected at coarser resolutions. Our findings therefore imply that the genetic architecture of complex diseases is shaped by regulatory effects active in restricted cellular contexts—effects that are often missed in bulk analyses.

These properties are consistent with an evolutionary model in which regulatory variants that broadly affect the expression of disease-relevant genes are more likely to be deleterious and subject to negative selection. As a result, such variants would remain at very low frequencies in the population, limiting their detection in both GWAS and eQTL studies. By contrast, variants whose regulatory effects are restricted to specific cellular contexts may evade such selective pressures, allowing them to persist at higher frequencies and be more readily detected. This model provides a potential evolutionary explanation for the observed enrichment of GWAS loci among eQTLs mapped at the cell-type level.

Our findings inform future eQTL mapping for GWAS effector genes. Although cell-type resolution is clearly advantageous, detection power remains restricted by per-annotation cell counts. Despite analysing 2.2 million cells, 57% of our cell types (for example, all stromal populations) yielded fewer than 100 cells per donor, limiting discovery in rare or disease-specific contexts that are often missed by bulk mapping. Consequently, although we nominated effector genes for more than 50% of known IBD loci, larger single-cell diseased cohorts will improve discovery and more precisely map disease-driving cellular dysregulation. Furthermore, adopting multi-variant colocalization methods (for example, coloc-SuSiE^82^) to relax our single-causal-variant assumption is likely to uncover additional independent signals and novel effector genes.

Larger and more deeply powered single-cell eQTL studies will also enable more precise interpretation of regulatory mechanisms at individual loci, particularly where risk variants affect pleiotropic pathways across multiple cell types. Several of the loci that we identified converge on the Notch signalling pathway, but do so through distinct mechanisms and in different cellular compartments. *MAML2* and *ZMIZ1* encode regulators of Notch-dependent gene transcription, and at both loci, reduced expression in dendritic cells that colocalized with increased IBD risk. By contrast, at the *PSEN2* locus, the IBD risk allele was associated with increased expression across epithelial and endothelial cells, which is likely to enhance Notch activity. This duality may help explain the mixed outcomes of γ-secretase inhibition in clinical trials, where beneficial anti-inflammatory effects could be counterbalanced by epithelial or vascular side effects.

A complementary mechanism suggested by our data involves disrupted epithelial renewal and stem cell maintenance. Although barrier dysfunction is a well-established feature of IBD, prior models have largely focused on defects in epithelial adhesion. Our results point to an additional axis of risk involving impaired regeneration, with IBD risk alleles colocalizing with eQTLs affecting genes such as *RASGRP1*, *MYC*, *LPIN3*, *RPS14* and *FUBP1* that regulate Wnt signalling, epithelial proliferation and mucosal repair. Many of these colocalized signals were assigned to colonocytes and motivate the hypothesis that disruption of epithelial renewal and stem cell maintenance in the absorptive epithelial cells of the rectum may be particularly relevant to IBD pathogenesis—consistent with work highlighting that Wnt signalling is reduced in patients with CD, independent of inflammation^83^. Regulators of both Notch and Wnt signalling also harbour rare variants that predispose to monogenic forms of IBD, such as *FERMT1*, *XIAP*, *ADAM17*, *IL2RA*, *ITCH* and *TGFBR2*^84–89^. By more than doubling the number of Notch and Wnt pathway IBD GWAS effector genes, we strengthen evidence that these pathways have a role in IBD, underscoring the value of cell-type-level resolution for dissecting the roles of pleiotropic pathways in complex disease. Further experimental investigation is now required to test these hypotheses and establish causality.

This study demonstrates the power of single-cell eQTL mapping in disease-relevant tissues to resolve the cellular and regulatory architecture of complex diseases and traits. By systematically comparing genetic effects across cellular resolutions, we show that disease-associated variants are disproportionately enriched among eQTLs detected at the cell-type level, and often act through context-restricted regulatory mechanisms. Although true evidence for the causality of our colocalizations can only be obtained from experimental, genetic knockout of nominated genes in the relevant cellular contexts, these findings provide a framework for interpreting non-coding GWAS loci with greater precision, constructing novel hypotheses about disease pathogenesis, and highlight the importance of sampling both healthy and inflamed tissues. As single-cell technologies continue to scale, the increased ability to map genetic effects at cell-type resolution will enable more complete, mechanistically informed and therapeutically relevant interpretations of complex disease association signals.

## Methods

### Patient recruitment and single-cell sequencing

This study was approved by the National Health Service (NHS) Research Ethics Committee (Cambridge South, REC ID 17/EE/0338). Written informed consent was given by all participants.

296 individuals without IBD and 125 individuals with CD were recruited at Addenbrooke’s Hospital, Cambridge, UK. Patients with CD were required to have a history of ileal disease involvement. During routine endoscopy, gastrointestinal biopsies were collected from the rectum and terminal ileum, and blood samples were drawn after the procedure. Terminal ileal biopsies were obtained from 243 individuals without IBD and 119 individuals with CD. Ileal biopsies from patients with CD included both inflamed and uninflamed tissue. Rectal biopsies were collected from 275 individuals without IBD. Where possible, rectal and ileal samples were collected from the same healthy individuals, and blood and ileal samples were collected from the same patients. For gastrointestinal biopsies, we have developed a gentle single-cell dissociation protocol that minimizes transcriptional changes and cell stress or death during cell isolation. Early samples were processed using version 1 of the protocol, whereas most of the samples were processed using version 2^90,91^. Whole blood was collected for scRNA-seq from 95 individuals with CD. Peripheral blood mononuclear blood cells (PBMCs) were isolated from the whole blood using the EasySep Direct Human PBMC Isolation Kit (StemCell Technologies).

Because IBD tissue displays increased cellular heterogeneity due to immune infiltration, we targeted 6,000 and 3,000 cells for CD and healthy biopsies, respectively. This is to ensure adequate representation of diverse cell populations and to maintain statistical power for expression quantification and eQTL mapping across annotations. scRNA-seq was performed using 3′ 10X Genomics Chromium kits (v3.0 and v3.1) according to the manufacturer’s instructions. Libraries were sequenced to a target depth of 50,000 reads per cell. CellRanger (v7.2.0) was used to align reads to the GRCh38 human genome reference with Ensembl v93 transcript definitions (GRCh38-3.0.0 reference file provided by 10X Genomics), and to generate cell-by-gene count matrices. The greater number of cells targeted for patients with CD is confirmed in the terminal ileum samples from the ‘atlasing’ dataset (see ‘Single-cell RNA sequencing quality control and cell clustering’) (Wilcoxon *P* value < 2.2 × 10^−16^), with no discernable influence on sequencing depth, as indicated by median number of counts per sample (*P* = 0.27) (Supplementary Fig. 6).

### Single-cell RNA-sequencing quality control and cell clustering

Cell count matrices were initially subset to retain droplets likely to contain cells and corrected for ambient RNA using CellBender v2.1^92^, as previously described^76^. Scrublet v0.2.1^93^ was then used to identify and remove potential multiplets. All subsequent quality control, normalization, and clustering steps were performed with Scanpy v1.9.6^94^, including normalizing to library sizes of 10,000 total counts using the function sc.pp.normalise_per_cell and log transformation using sc.pp.log1p to produce the ln[cp10k + 1] count matrix.

A lineage-sensitive, relative quality control strategy was applied to account for technical and biological variation across tissues and lineages (Supplementary Fig. 1). First, the input dataset (4,099,804 cells) was aggregated and subjected to an initial round of quality control. Cells were removed if they had fewer than 250 genes expressed, fewer than 500 total counts, or more than 20% or 50% of reads originating from the mitochondrial genome, for blood-derived and non-blood-derived cells, respectively. This left a total of 2,725,568 cells. Genes were then removed if they were not expressed in at least five cells. A total of 5,000 highly variable genes were then classified within each sample and merged using sc.pp.highly_variable_genes and flavour = seurat. Ribosomal, mitochondrial and immunoglobulin genes were then excluded from the highly variable set.

These data were then subject to an initial round of integration and clustering. Data were integrated using the scVI v0.16.3^95^ function scvi.model.SCVI on raw count data of highly variable genes. This was trained on 2 layers with 30 latent variables and gene likelihood modelled as a negative binomial distribution. A total of 50 nearest neighbours was calculated per cell using the function sc.pp.neighbors to generate an adjacency matrix, which was used for calculation of both an initial uniform manifold approximation and projection (UMAP) embedding using function sc.tl.umap with both a minimum distance and spread of 0.5, and Leiden clusters at a resolution of 0.03 using sc.tl.leiden. Initial clusters were then grouped into cell lineages, based on the expression of canonical marker genes (Supplementary Fig. 7), including: B cells, epithelial cells, mast cells, mesenchymal cells, myeloid cells, platelets and T/ILC cells. Markers for these annotations are described in ‘Annotation of cell clusters’.

Within each cell lineage (B cells, epithelial cells, mesenchymal cells, myeloid cells (not including platelets or mast cells) and T/ILC cells), we then further subset cells based on relative thresholds for quality control parameters. Cells with number of unique genes expressed, total counts and percentage of genes originating from the mitochondrial genome that lay outside the median ± 2.5 median absolute deviations were removed. For the epithelial lineage, the median deviation was far greater than that for non-epithelial, and so a window of 2 median absolute deviations was utilized. Additionally, epithelial cells from samples with lower overall depth (indicated by median number of genes expressed per cell <2,000) were removed from the epithelial population only, and any infiltration of blood-derived cells were also removed. Highly variable genes were then re-selected from the full initial set within each lineage independently, and re-integrated using scVI as before, computing 20 nearest neighbours for non-epithelial cells and 50 nearest neighbours for epithelial cells.

Cell types were then identified by clustering within each lineage independently. As described previously^76^, the resolution at which final clusters were derived was determined in a data-driven manner. Cells from each lineage were clustered at resolutions of 0.1 to 1.5 (interval of 0.1) using the sc.tl.leiden function. To determine the reproducibility of the clusters detected at each resolution, two-thirds of the data were then used to train a single layer dense neural network model using keras v2.4.3, and the per-cluster matthews correlation coefficient (MCC) of the fit was assessed in the remaining one-third of data. After clusters that were either comprising >40% cells from a single sample or showed outlying quality control parameters were removed, we selected the resolution at which all remaining clusters had a MCC > 0.75. Additionally, reproducible (MCC > 0.75) clusters demarcated by the expression of genes indicative of poor quality cells, such as *MALAT1*^96^, and cross-lineage marker genes were also removed. The resolutions utilized for the final cell clusters were: epithelial cells, 0.9; mesenchymal cells, 1.0; myeloid cells, 0.7; B cells, 0.8; and T/ILC cells, 1.1.

Finally, the additionally filtered and clustered per-lineage clusters were then recombined with the mast and platelet cells. This population, known as the ‘atlasing’ dataset, comprised 1,852,681 cells and was re-integrated as described above. With scope to auto-annotate cells with reliable transcriptional profiles that may have been removed by the strict, within-lineage quality control, a CellTypist v1.6.2^24^ model was trained on the atlasing dataset. This was assessed by quantifying the proportion of cells that were correctly assigned to the same annotations in the initial, manual annotation. Overall, this was achieved at a rate of 97% (median 98%), with only 4 clusters achieving an accuracy <90% and only one <80% (tissue plasma B CD38^hi^ IgG IgA, 87.8%; tissue plasma B CD38^low^, 79%; blood atypical B cells, 88.7%; tissue memory B *HIVEP3*^*+*^, 80%). This model was then used to auto-annotate the full set of cells that passed the first round of quality control, and filtered for those with confidence <0.5, resulting in a final dataset that after intersection with genotyped individuals, results in 2,196,874 cells.

### Annotation of cell clusters

As described above, cells from all tissues were initially clustered into cell lineages after the first round of quality control and integration. These were grouped and annotated on the basis of canonical marker genes (Supplementary Fig. 8); Epithelial cells were defined by high expression of *EPCAM*, *CDH1* and* KRT19*; mesenchymal by expression of *COL1A1*, *COL1A2*, *COL6A2* and *VWF*; B cells by expression of *CD79A*, *MS4A1* and *CD79B*; plasma B cells by expression of *MZB1* and* JCHAIN*; T and T/ILC cells by expression of *CD3D*, *CD3E*, *CD3G*, *CCR7*, *IL7R*, *TRAC* and *NCAM1*; myeloid cells by expression of *ITGAM*,* CD14*,* CSF1R* and* TYROBP*; mast cells by expression of *TPSAB1*, *TPSB2* and *CPA3*; and platelets by expression of *RUNX1*, *GATA2*, *HDC* and *SLC24A3* (but low expressors of mast cell genes). For within-lineage clustering, B cells and plasma B cells were combined into a single lineage, with mast cells and platelets excluded.

Following clustering within lineage, cell types were first annotated by major population using a series of canonical markers, detailed in subsections below. In the case of T/ILC and mesenchymal populations, this matched their clustering lineage, however; epithelial cells were further subdivided into enterocyte, colonocyte, stem cells and secretory cells; the B cell lineage was re-divided into B cells and plasma B cells; and the myeloid cell lineage were combined with mast and platelet cells. To interpret individual cell clusters, marker genes were calculated within major populations or across targeted subsets of clusters using scanpy’s sc.tl.rank_genes_groups function (method = ‘wilcoxon’). The significant results (FDR < 0.05), along with a discriminative subset that were filtered using scanpy’s sc.tl.filter_rank_genes_groups (min_in_group_fraction=0.5, max_out_group_fraction=0.3, min_fold_change=1.5), were used to annotate and label clusters using expert knowledge. Enrichment of specific populations in a single tissue, or combination of tissues, was determined by the overall proportion of cells from each site (Extended Data Fig. 2).

#### Epithelial cell lineages

Genes used to demarcate epithelial lineages are presented in Supplementary Fig. 8a. The stem cell major population was defined by expression of *LGR5*, *OLFM4*, *ASCL2*, *SMOC2* and *RGMB*. One of these populations was specific to the rectum, while two were specific to the terminal ileum, and thus are described as ‘rectal’ and ‘intestinal’, respectively. Within the intestinal subsets, one showed greater expression of *MKI67*, and the other had greater expression of *OLFM4* and *LGR5*, which were thus used to discriminate these populations.

Absorptive epithelial cells were also largely terminal ileum- or rectum-specific (Extended Data Fig. 2). The majority show clear separation in dimensionality reduced space (Fig. 1b), and were grouped into the enterocyte (high expression of *RBP2*,* ANPEP* and* FABP2*) and colonocyte (high expression of *CA2*,* SLC26A2* and* FABP1*) categories, respectively. The one exception was a population composed of cells from both predominantly diseased terminal ileum and rectal origin, with high expression of *OLFM4*, thus referred to as ‘epithelial progenitor OLFM4^hi^’, grouped into the colonocyte major population due to their embedding in dimensionality reduced space (Fig. 1b). Both enterocyte and colonocyte populations were characterized by the relative expression of marker genes, with discriminative ones being added to their label, including *OLFM4* and* MKI67* (indicative of proliferation), *BEST4*, *CEACAM7*,* KRT20*,* IFI27*,* ALDOB* and *RBFOX1*.

Secretory cells were broadly more shared across the terminal ileum and rectum, but with some site enriched clusters (Extended Data Fig. 2); goblet cells were identified by expression of *MUC2*, *CLCA1* and *FCGBP*; tuft cells by expression of *POU2F3*, *SH2D6*,* LRMP* and *TRPM5*; Paneth cells by expression of *DEFA5* and* DEFA6*; enteroendocrine cells by expression of *NTS*,* PYY*,* GCG*,* ISL1*; and enterochromaffin cells by expression of *TPS1* and* CES1*.

#### Mesenchymal cell lineages

Markers used to demarcate mesenchymal cell subsets are displayed in Supplementary Fig. 8b. Fibroblast clusters were identified by their expression of *ADAMDEC1*,* PDGFRA*,* BMP4*,* LUM*,* COL1A2*,* COL3A1* and *COL1A1*, subsets of which were then delineated by their expression of *PI16*, *C3*,* CPM*,* NPY*,* GRIN2A* and* ADAMDEC1* (lamina propria fibroblast), and tissue specificity. Glial cells were identified by expression of *NRXN1*; smooth muscle cells by expression of *ACTA2*; pericytes by expression of *PDGFRB*, *RGS5* and *NDUFA4L2*; endothelial cells by expression of *PECAM1*, *VWF*, *PLVAP*, *ACKR1* and *CD36*. Endothelial cells were further divided into inferred residency by expression of high *TLL1, ACKR1* and *PECAM1* (venous), modest *TLL1* and *PECAM1* (lymphatic) and high *CD36* greatest *PECAM1* and *PLVAP* (capillary). Myofibroblasts were identified by expression of *ACTA2* and *MYH11*, which were then delineated by relative expression of *GREM2* and *ADGRL3*.

#### B cell lineages

Markers calculated within plasma B cells were not very discriminatory, however these clusters show a gradient of *CD38* expression, which is therefore outlined in the label (Supplementary Fig. 8c). Each of these include a majority of IgA-expressing cells, with one cluster containing a minority of high IgG-expressing cells. For the non-plasma B cells; atypical B cells were identified by their expression of *FCRL2*, *ITGAX* and *TBX21*; naive B cells by expression of *IGHD* and *FCER2*; germinal centre/plasmablast B cells by expression of *CD19* and* TCL1A* (for which we also identified a *MKI67*-expressing, proliferative subset); memory B cells by expression of *BANK1* and* FCRL2*. The several clusters of memory B cells were also distinguished by the relative expression of marker genes; *CD27*, *HIVEP3*, *PTPRJ*, *PDE4D* and *TEX9*.

#### Myeloid cell lineages

Across the myeloid cell lineage, we identified several distinct clusters of monocytes, macrophage and dendritic cells (Supplementary Fig. 8d). Monocytes were defined by high expression of *S100A8* and *S100A9* and were broadly divisible by anatomical location. Blood monocytes were distinguished by expression of *CD14* and *FCGR3A* (encoding CD16) and tissue monocytes by expression of *SOD2*, *CXCL9* and *CXCL10*.

Macrophages were predominantly identified in the terminal ileum and rectum and were identified by high expression of *ITGA4*,* CSF1R* and *MAF*. Macrophage subpopulations could thereafter be distinguished by relative expression of *C1QA/B/C*, *CD163*,* MRC1*,* SELENOP* and *HLA-DRA*.

Three separate dendritic cell subsets were identified: cDC1s were identifiable from expression of *CLEC9A* and *XCR1*, cDC2s were identified by expression of *CLEC10A*, and pDCs identified by *IL3RA*.

#### T cell and ILC lineages

Within the T cell and ILC lineage, T cells were defined by expression of *CD3D*,* CD3E*,* CD3G*,* TRAC*, *TRBC1* and* CD96*, refined by *CD4*,* CD8A*, *CD8B*,* CD3* and *GZMK* expression (Supplementary Fig. 8e). Naive subsets were identified by high expression of *SELL*; γδ (GD) by expression of *TRGC2* and *TRDC* (for which we also defined gut- and blood-enriched subsets referred to as ‘GD T KLRG1’ and ‘Tissue resident GD T’; see Extended Data Fig. 2); T_reg_ cells by expression of *FOXP3*and* CTLA4*; Effector memory subsets divided by expression of *TIGIT* and *THEMIS*; tissue resident memory with high expression of *TRGC2*. Two T cell clusters that did not express *CD8* or *CD4* were also identified, one of which strongly expressed *TRDC*, and the other that strongly expressed *GZMK*. NK1 cells were identified by expression of *CX3CR1* and *FCGR3A*, and NK2 cells by *XCL1*, *XCL2* and *CD44*. ILCs were identified by expression of *IL1R1*,* ALDOC*,* LST1* and *RORC*.

### Pseudobulking

Pseudobulk expression data were obtained by calculating the mean ln[cp10k + 1] expression across cells of a given annotation, as per previous sc-eQTL mapping benchmarks^97^. This was performed at ‘all cells’, major population and cell-type resolutions, both within and across anatomical sites (Fig. 1a). Expression data were limited to samples with 5 or more cells in the given annotation and only taken forward for further analysis if there were 30 or more individuals contributing to the annotation. In addition, the genes present in each annotation matrix were limited to those with a total count greater than 0 in at least 20% of pseudobulked samples, or 40% of pseudobulked samples for ieQTL mapping. Pseudobulked expression matrices were then inverse normal transformed at the gene level prior to eQTL mapping.

### DNA collection and quality control

On the day of the endoscopy, blood (or gastrointestinal tissue, where blood was unavailable) was collected from individuals for DNA array genotyping. DNA was extracted from blood or tissue samples and genotyped with the Applied Biosystems UK Biobank Axiom Array in five batches of samples by YourGene Health. Genotypes were called jointly across all five batches of samples to mitigate potential batch effects. Genotype data were filtered to remove low missingness, duplicate samples, variants not in Hardy–Weinberg equilibrium, individuals with non-European ancestry (as predicted by KING^98^) and first-degree relatives. Variants were lifted over from human genome build 37 to human genome build 38 with LiftOver prior to imputation being performed using the Michigan Imputation Server with the TOPMed reference panel^99,100^. Imputed variants with imputation quality lower than 0.3 were removed.

### *cis*-eQTL mapping

eQTL mapping was carried out using TensorQTL^101^ permutation and nominal modes with default parameters. *cis*-eQTLs were mapped for variants with minor allele frequency > 0.05 that were within a 2 Mb window (1 Mb in either direction) of the TSS of a given gene. The following model was used for mapping *cis*-eQTLs:

$${\mathrm{Ex}}_{G,C} \sim \mathrm{GT}+{\mathrm{PC}}_{\mathrm{GT}}+{\mathrm{PC}}_{{\mathrm{Ex}}_{C}}$$

where Ex is gene expression, *G* denotes a gene and *C* is an annotation. GT denotes the genotype alternative allele dosage of the individual (typed or imputed) and PC refers to principal components of genotype and annotation expression. To detect conditionally independent signals, a forwards regression framework was applied, as described previously^13^.

For interaction *cis*-eQTL mapping, a stricter minor allele frequency filter of 0.1 was applied to each variable level and an expanded model was used:

$${\mathrm{Ex}}_{G,C} \sim \mathrm{GT}+I+\mathrm{GT}:I+{\mathrm{PC}}_{\mathrm{GT}}+{\mathrm{PC}}_{{\mathrm{Ex}}_{C}}$$

where *I* is the interaction variable and GT*:I* indicates the coefficient for the interaction between genotype and the interaction variable, reported in results. We tested for interactions with sex, age (in bins of 5 years), disease status (CD or healthy), smoking status, and inflammation status (TI-SES-CD score, as defined previously^76^). The reported sex of each sample was verified by Y chromosome carriage and pseudobulk expression of *XIST* versus all genes on the Y chromosome.

Five genetic principal components were included to control for population structure between individuals. The number of expression principal components to include was determined by repeating the analysis several times, increasing numbers of principal components from 2 to 100 in intervals of 2. The optimal model was selected based on the number of expression principal components that returned the greatest number of eGenes (genome-wide FDR < 0.05), with ties broken by the model with fewest covariates (see ‘Multiple hypothesis testing correction’). For the interaction analysis, any expression principal components which were correlated with the interaction variable were removed from the model (Pearson’s *R* > 0.25).

### Multiple hypothesis testing correction

Correction of *P* values to account for multiple hypothesis testing in eQTL mapping was done within each cellular annotation independently. This varied slightly for primary, conditional and interaction effects and is detailed below.

#### Multiple testing correction of primary signals

1. Within each gene, multiple variants are being tested for their association with expression. To account for this, expression is permuted 1,000 times, and associations with each variant are recalculated. This procedure allows estimation of a null distribution of associations, and calculation of beta-approximated empirical *P* values (beta-*P*). The variant with the smallest beta-*P* value is then selected for each gene.
2. Multiple genes are being tested for eQTL associations within each annotation. A *q*-value correction^102^ is then applied to the variants with the smallest beta-*P* value per gene, allowing estimation of a genome-wide FDR. We then apply a threshold of FDR < 0.05 to account for both the number of variants being tested per gene and the number of genes, while accommodating for the varied underlying null distributions.

#### Multiple testing correction of conditionally independent effects

For genes with a significant eQTL association in a cellular annotation (genome-wide FDR < 0.05), subsequent signals are then tested in a forward regression framework by iteratively incorporating variants into the tested model. Firstly, the minimum beta-*P* value with genome-wide FDR < 0.05 is defined across all genes (min-beta-*P*). During each round of conditional analysis, associations between variants and gene expression level are re-tested and permuted, and the variant with the smallest beta-*P* value is selected for each gene. If this variant has a beta-*P* < min-beta-*P* from the primary analysis, it is declared a conditionally independent signal and included into the model being tested for that gene in the next round. Because the beta-*P* must always be less than min-beta-*P*, conditional signals are iteratively mapped per gene until they would fail to pass the genome-wide FDR < 0.05 from the primary analysis.

#### Multiple testing correction of interaction effects

Following ieQTL testing, the number of effectively independent variants being tested is estimated for each gene using eigenMT^103^. Nominal *P* values for ieQTL effects are then adjusted using the Bonferroni method to account for the number of effectively independent tests being performed (emt-p). The variant with the minimum emt-p value is then selected for each gene, and a Benjamini-Hochberg adjustment is then applied to account for the number of genes being tested (adjusted *P*). Reported ieQTL effects all have adjusted *P* < 0.05.

### Aggregation of QTL effects

Lead variants for the same genetic association may differ across conditions or QTL types (primary, conditional or interaction) due to minor changes in significance. To group lead variants across all tests and define comparable QTL signals, linkage disequilibrium clumping was performed using the ‘–clump’ flag in plink v1.90b6.27^104^ and a threshold of *r*^2^ > 0.5 for variants within 1 Mb with no additional significance filter. This was performed using lead variants from conditionally independent eQTLs (genome-wide FDR < 0.05), ieQTLs (adjusted *P* < 0.05) and lead variants after intersection with GWAS summary statistics to help map colocalization events to eQTLs.

### Inferring capture of our eQTLs in other studies

Harmonized nominal summary statistics for eQTLs from every GTEx v8 bulk RNA sequenced tissue(GTEx Consortium 2020) and sc-eQTLs for peripheral blood mononuclear cells of Yazar et al.^18^ were downloaded from eQTL catalogue (https://www.ebi.ac.uk/eqtl/, study IDs QTS000015 and QTS000038, respectively). For eQTLs detected in each of our annotations, we then calculated the enrichment of these associations among low *P* values, π1 (ref. ^102^), in each external dataset using qvalue v2.34.0^105^. This was also performed between eQTLs detected at each resolution in our dataset with eQTLs from GTEx tissues. For comparison with Yazar et al.^18^, cell types were grouped into major populations based on nomenclature (B - intermediate, memory, naive; Myeloid - CD14_Mono, CD16_Mono, Platelet, cDC2, pDC; T - CD4_CTL, CD4_Naive, CD4_TCM, CD4_TEM, CD8_Naive, CD8_TCM, CD8_TEM, MAIT, NK, NK_CD56bright, NK_Proliferating, Treg, dnT, gdT; unknown - HSPC, Haematopoietic stem; Plasma - Plasmablast). A detailed summary of these comparisons is shown in the Supplementary Note.

### Colocalization

Colocalization was performed in a pairwise fashion between sc-eQTL summary statistics and GWAS summary statistics using coloc v5.2.2 and the coloc.abf function^32^. GWAS summary statistics from de Lange et al.^3^ were downloaded from GWAS Catalog (https://www.ebi.ac.uk/gwas/home) (see ‘Data availability’ and ‘Code availability’). Analyses were conducted in 2 Mb *cis* windows centred around the lead single-nucleotide polymorphism (SNP) of each eGene with a permuted FDR-corrected *P* value < 0.05 and using GWAS loci which were significant at the genome-wide suggestive threshold (*P* value < 5 × 10^−5^). Analysis was conducted using default prior probabilities of p1 = p2 = 10^−4^ and p12 = 10^−5^ where p1 and p2 are the prior probabilities of true genetic associations in each input dataset and p12 is the probability of true associations in both datasets. For colocalization between GWAS and ieQTLs, approximate Bayes factors were computed from the effect sizes and standard errors of the GT:*I* term, and the prior standard deviation on the true effect size was estimated as 0.3 based on the distribution of GT:*I* effect sizes across all nominal results on chromosome 21. A posterior probability of colocalization (PP_h4_) greater than 0.75 was considered to be sufficient evidence for colocalization of genetic signals. Colocalizations which fell within the HLA on chromosome 6 (build 38, positions 28510120–33480577) were discarded as likely false positives due to the complicated patterns of linkage disequilibrium in this region. Colocalization events were assigned to the closest existing IBD locus, if the lead colocalized GWAS variant was within 500 kb of a known IBD locus as defined by the interval comprising ±500 kb containing: (1) the most likely causal variant from published fine-mapping analysis^3,106^; or alternatively, if the locus has now been previously fine mapped, (2) from the GWAS lead variant as defined in refs. ^3,34^.

We used the R package otargen (v1.1.5) to download all colocalizations between genes with molecular QTLs and IBD, CD and UC GWAS loci from the Open Targets genetics portal (accessed 22 January 2025). We retained any colocalizations with PP_h4_ > 0.75 and assigned to IBD loci as previously described for our colocalizations. For comparison of disease effector genes nominated in this work with that of Cuomo et al.^35^, as their colocalization is limited to IBD and with a threshold of PP_h4_ > 0.8, we applied the same filtration to our own associations.

To check if disease effector genes identified at high resolutions were more specifically expressed, genes were grouped into the lowest resolution at which a colocalization was detected on their expression. The maximum specificity of gene expression at the cell-type level was then calculated using CELLEX^107^, and compared across resolutions using a Wilcoxon test.

Detection of colocalization events between two phenotypes is highly dependent on the significance of those effects. This is therefore underpinned by the statistical power in those tests, driven by the total number of observations. As this varies substantially for sc-eQTL tests (Fig. 1b and Supplementary Fig. 3), inherent power to determine eQTL and colocalization events also varies. To circumvent the influence of varied statistical power in the nomination of cell types within which colocalization events are active, for each colocalization, we quantified the proportion of variance in disease effector gene expression (*r*^2^) that was explained by the lead eQTL variant after intersection with the GWAS variants across each condition. This was calculated by linear regression of disease effector gene expression in the inverse normal transformed pseudobulk data on genotype.

To assess the evidence for enrichment of Notch and Wnt pathway regulators in our disease effector genes, we performed a gene ontology over-representation test of ‘Notch signalling pathway’ (GO:0007219) and ‘Wnt signalling pathway’ (GO:0016055) in our disease effector genes against a background of all eGenes using clusterProfiler v4.10.1^108^. This was performed using default parameters except minGSSize=3, pvalueCutoff = 1, qvalueCutoff = 1. These gene sets were also used to quantify the number of disease effector genes previously and presently nominated in these pathways.

### Enrichment of eQTLs in genomic annotations

To calculate the enrichment of lead variants from eQTLs in ENCODE and FANTOM genomic annotations, we followed previous approaches^12^, available on GitHub https://github.com/hakha-most/gwas_eqtl. In brief, eQTLs were first grouped by the minimum resolution at which they were detected, and their presence in functional elements was calculated. To calculate bootstrapped confidence intervals, we then sampled with replacement from independent linkage disequilibrium blocks (calculated previously^109^) that contained eQTLs detected at each resolution one thousand times. Enrichment values in different annotations were then first normalized to that of random variants, before values in enhancers being normalized to those in promoters, in order to identify relative enrichment in these annotations specifically. Distal and proximal enhancer-like annotations from ENCODE were grouped together. *P* values were calculated by two-tailed *t*-tests comparing the bootstrapped distributions, with the 25th, 50th and 75th percentiles displayed in the enrichment plot.

### Therapeutic evidence analysis

We downloaded data on existing therapeutics and their clinical approval stage from ChEMBL and Open Targets^68,110^. Therapeutics were grouped into broad disease terms using the Ontology Lookup Service^111^ and filtered for those which directly targeted genes which we had colocalized with UC, CD or IBD (PP_h4_ > 0.75, within 0.5 Mb of existing IBD locus) but had not yet been trialed as IBD treatments according to ChEMBL.

### Reporting summary

Further information on research design is available in the Nature Portfolio Reporting Summary linked to this article.

## Online content

Any methods, additional references, Nature Portfolio reporting summaries, source data, extended data, supplementary information, acknowledgements, peer review information; details of author contributions and competing interests; and statements of data and code availability are available at 10.1038/s41586-026-10627-z.

## Supplementary information

Supplementary Information Supplementary Methods, Supplementary Figs. 1–11 and Supplementary Notes.

Reporting Summary

Supplementary Table 1 Gene set enrichment analysis results for ieGenes detected with each interaction covariate in each cell type.

Supplementary Table 2 Summary of genes nominated by colocalization in IBDverse at each IBD/CD/UC GWAS locus (PP_h4_ > 0.75), compared with the genes nominated by Open Targets genetics.

Supplementary Table 3 Summary of the variance in disease effector gene expression that is explained by the colocalizing QTL variant across cell types.

Peer Review File

## Figures and Tables

**Extended Data Table 1:**
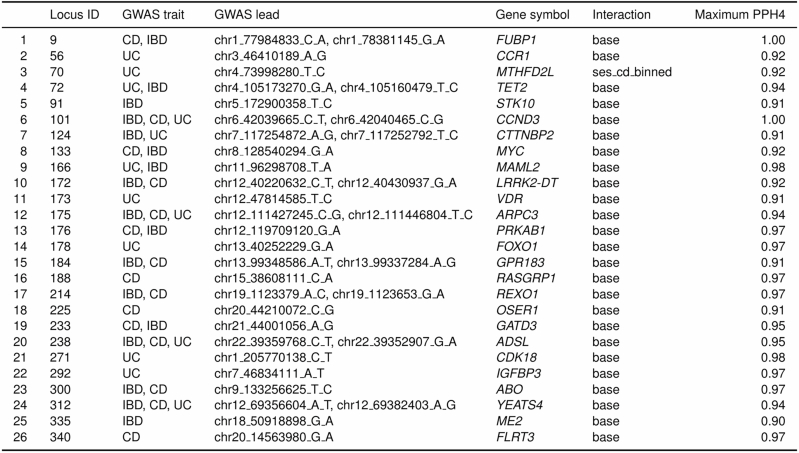
Novel disease effector genes of high confidence

IBD loci for which we identify a high confidence colocalization (PPH4 > 0.9) between dysregulation of a single gene with IBD, CD or UC susceptibility, and no disease effector gene has been nominated at this locus by colocalization previously in the Open Targets Genetics database. Extended summary statistics for all loci are available in Supplementary Material S2. GWAS lead = lead variant for GWAS signal after intersection with variants tested for eQTL. Maximum PPH4 = maximum posterior probability of colocalization observed for given eQTL and the GWAS signal in any condition. The primary GWAS signal in locus 300 is driven by rs8176719 (chr9:133257521:T:TC - p.Thr87AspfsTer107–ABO type 0*) so we cannot fully disregard that the colocalization we observe is mediated by the coding variant.

## Data Availability

We have centralized all interactive data exploration and file access at our project website: https://www.ibdverse.info/. We deposited public processed data (expression data from the atlasing and eQTL mapping cohorts, summary statistics from all eQTL, ieQTL and colocalization analyses, the CellTypist prediction model, and metadata) in the BioStudies database (http://www.ebi.ac.uk/biostudies) under accession numbers S-BSST2922 and E-MTAB-16986. scRNA-seq reads and genotype data are available in the European Genome-phenome Archive (EGA) under accession numbers EGAD00001015692 and EGAD00001016069, respectively, which are accessible following approval by the Sanger Data Access Committee.

## Code availability

Code used to perform all analyses is available at https://github.com/andersonlab/IBDVerse-sc-eQTL-code  (ref. ^112^). GWAS data is available from GWAS Catalog using accession numbers GCST004132 (CD), GCST004133 (UC) and GCST004131 (IBD).
