# Supplementary material for: Cell-type-resolved genetic variation shapes inflammatory bowel disease risk
Source: Nature. 2026 Jun 3;656(8126):129–39. doi: 10.1038/s41586-026-10627-z (PMC13441992; doi:10.1038/s41586-026-10627-z)
Supplement: Supplementary file 1 [file 41586_2026_10627_MOESM1_ESM.pdf]

nature portfolio

https://doi.org/10.1038/s41586-026-10627-z

Supplementary information

Cell-type-resolved genetic variation shapes inflammatory bowel disease risk

In the format provided by the authors and unedited

Supplementary Information

Cell-type-resolved genetic variation shapes inflammatory bowel disease risk

Alegbe, Harris et al.
Correspondence to Tim Raine (tim.raine@nhs.net) and
Carl Anderson (ca3@sanger.ac.uk)

Contents

1 Supplementary Methods 3
1.1 Fine-mapping eQTL and CD associations at the *RNF14* locus 3

2 Supplementary figures 4

3 Supplementary Note 11
3.1 Inferring the capture of our eQTLs in other studies 11
3.1.1 Capture of our eQTLs in GTEx 11
3.1.2 Capture of our eQTLs in Yazar *et al.,* (2022) 12
3.2 Fine mapping the *RNF14* locus 14

1 Supplementary Methods

1.1 Fine-mapping eQTL and CD associations at the *RNF14* locus

To better characterise genetic associations at the *RNF14* locus, we fine mapped and compared putative causal variants for various phenotypes in various subsets of samples (detailed in the Supplementary Note 3.2). The CD association was fine-mapped with SuSiE using default parameters of coloc-SuSiE[82] and linkage disequilibrium estimates from our cohort. Non-interaction, univariate, cis-eQTL effects were tested for variants within 1Mb of the TSS of *RNF14* in Enterocyte top villus cells for the 58 individuals with either 'mild' or 'severe' inflammation separately to the 262 uninflamed individuals. Cis-eQTL associations were then fine mapped using the Probabilistic Identification of Causal SNPs (PICS) algorithm[107] and linkage disequilibrium estimates derived from our cohort.

2 Supplementary figures

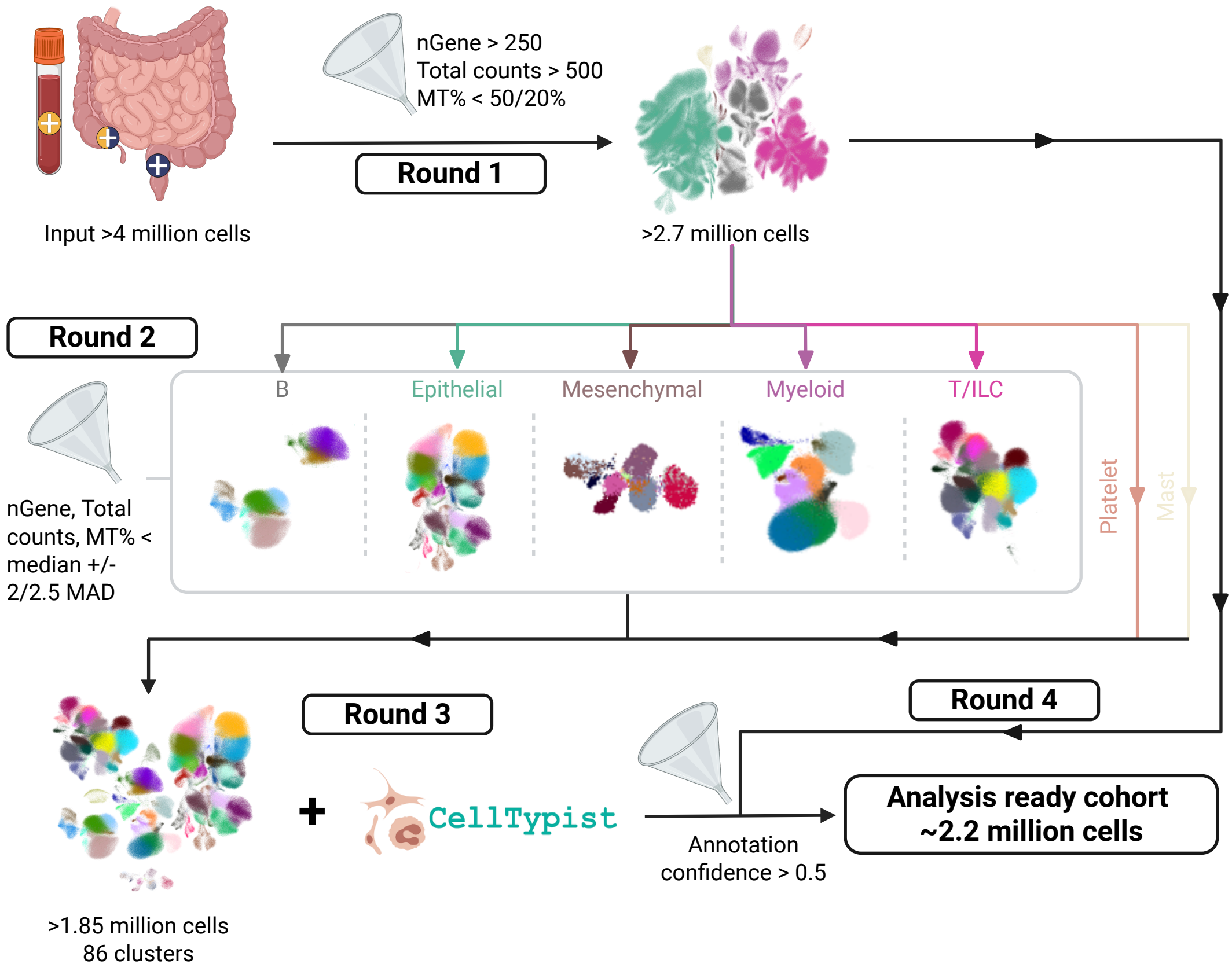

Supplementary Figure 1: **Overview of quality control, integration and clustering approach for scRNAseq data.** Analysis was performed across 4 rounds, a detailed summary of which is available in Methods. Cells were first filtered by basic quality control, integrated and clustered at a coarse resolution to identify broad lineages (see Supplementary Figure S7). These were then divided by lineage, and all except Platelet and Mast cells were subject to a second round of quality control using relative thresholds, before re-integration and clustering across a range of resolutions. The maximum resolution at which transcriptionally distinct populations of each lineage could be reproducibly obtained was then detected. Lineages were then recombined in a final round of integration, producing the total 86 cell-types. See Supplementary Figure S15 for outline of genes used to annotate individual cell-types. This was then used to derive a CellTypist model, which was used to annotate cells that passed quality control from the first round, producing the analysis-ready set. nGene = The number of genes expressed per cell, Total counts = total number of counts per cell, MT% = the percentage of counts mapping to genes in the mitochondrial genome, MAD = the number of median absolute deviations a cell's quality control parameter is from the median for that given parameter.

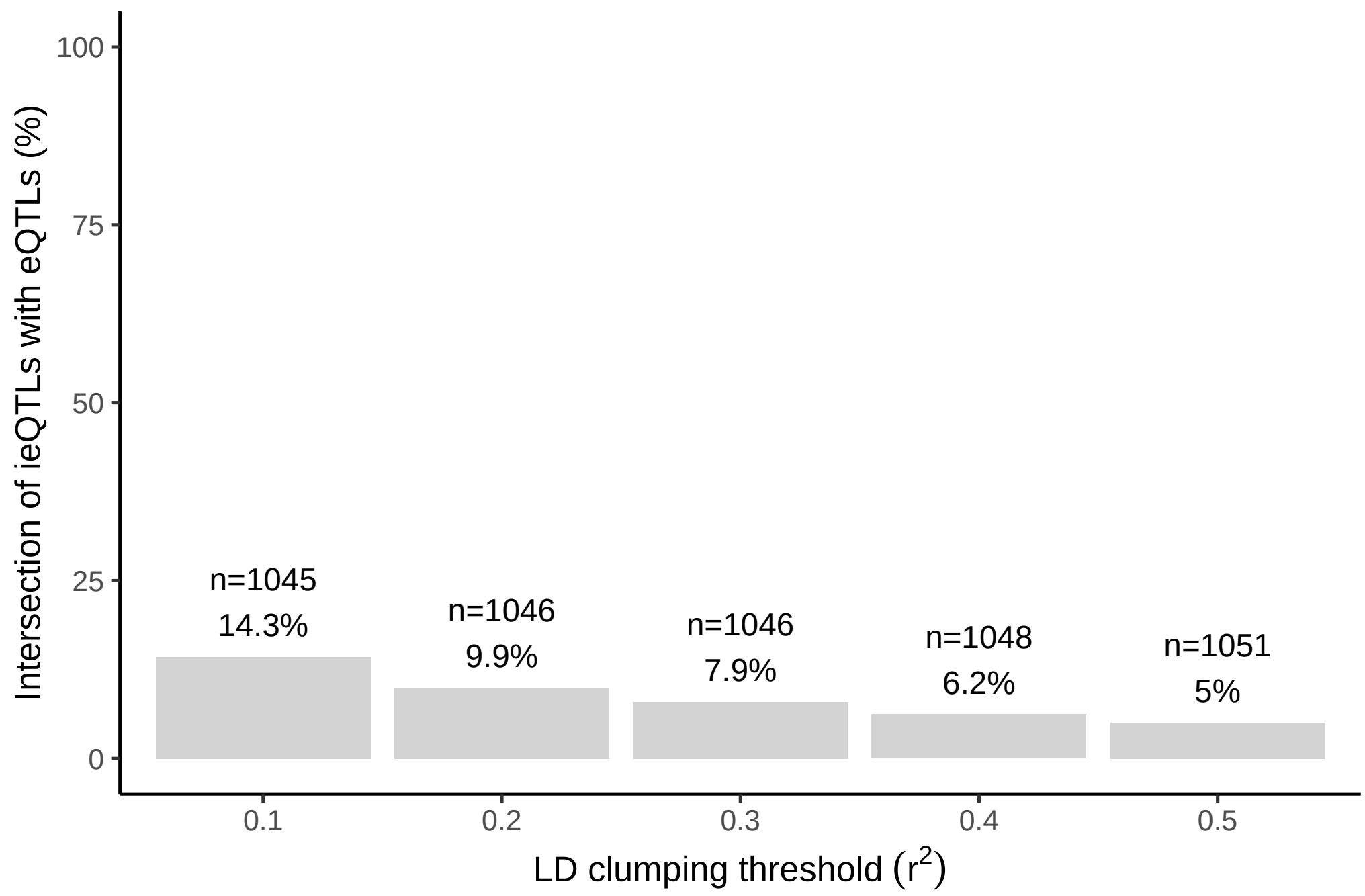

Supplementary Figure 2: **ieQTLs are largely distinct from eQTLs.** The total number of ieQTLs detected and the percentage which overlap eQTLs at different r^2 thresholds used for LD clumping (see Methods).

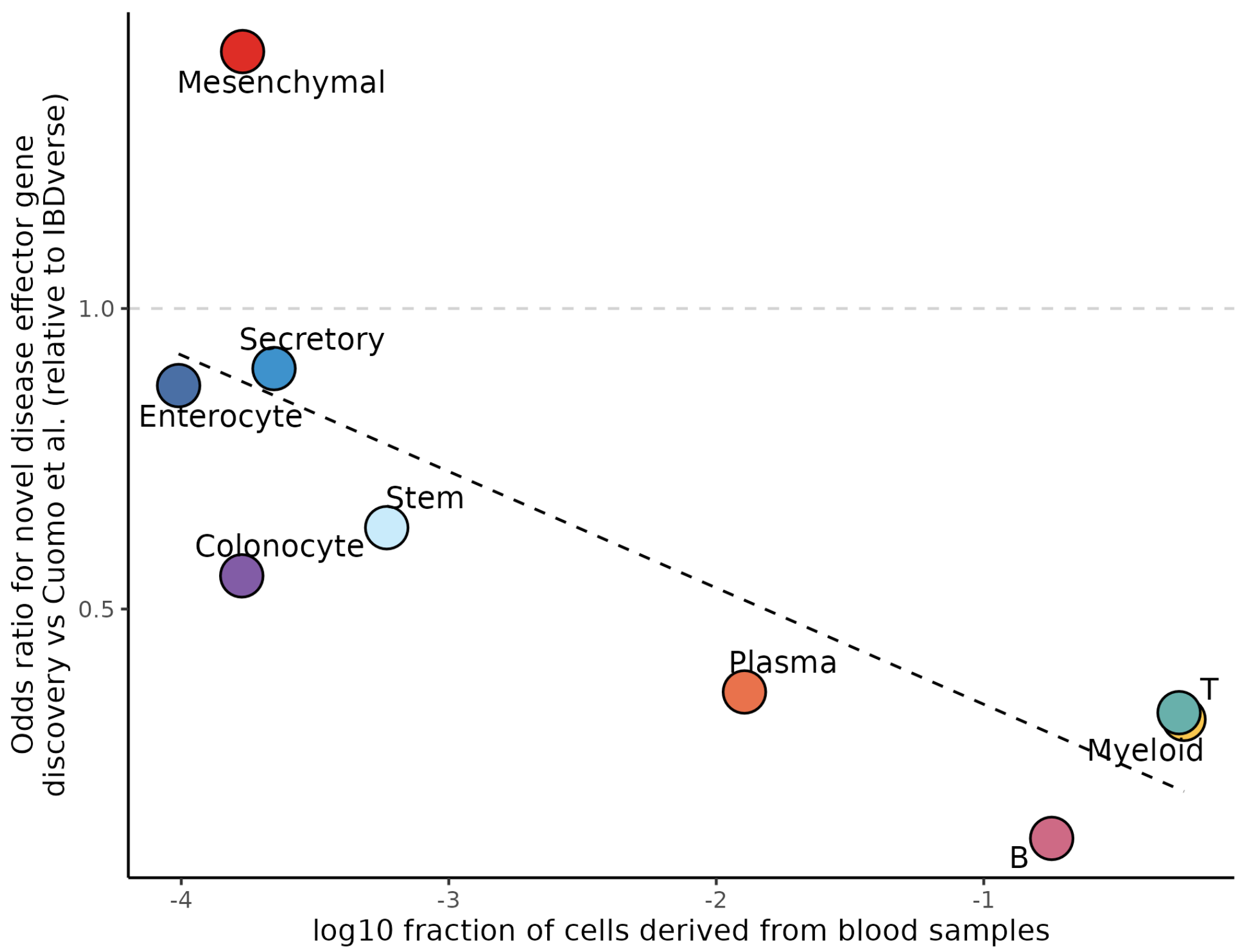

Supplementary Figure 3: **Novelty of IBDverse effector genes vs Cuomo et al (2025).** Relationship between the logarithmised proportion of cells in each major population that were derived from the blood (x-axis), and the likelihood that disease effector genes discovered in those major populations are novel compared to Cuomo et al., 2025 - presented as an odds ratio (OR) between novel disease effector gene discovery and effector gene discovery. The horiztonal line indicates OR=1 and therefore no enrichment. Linear regression slope = -0.194, p = 0.014.

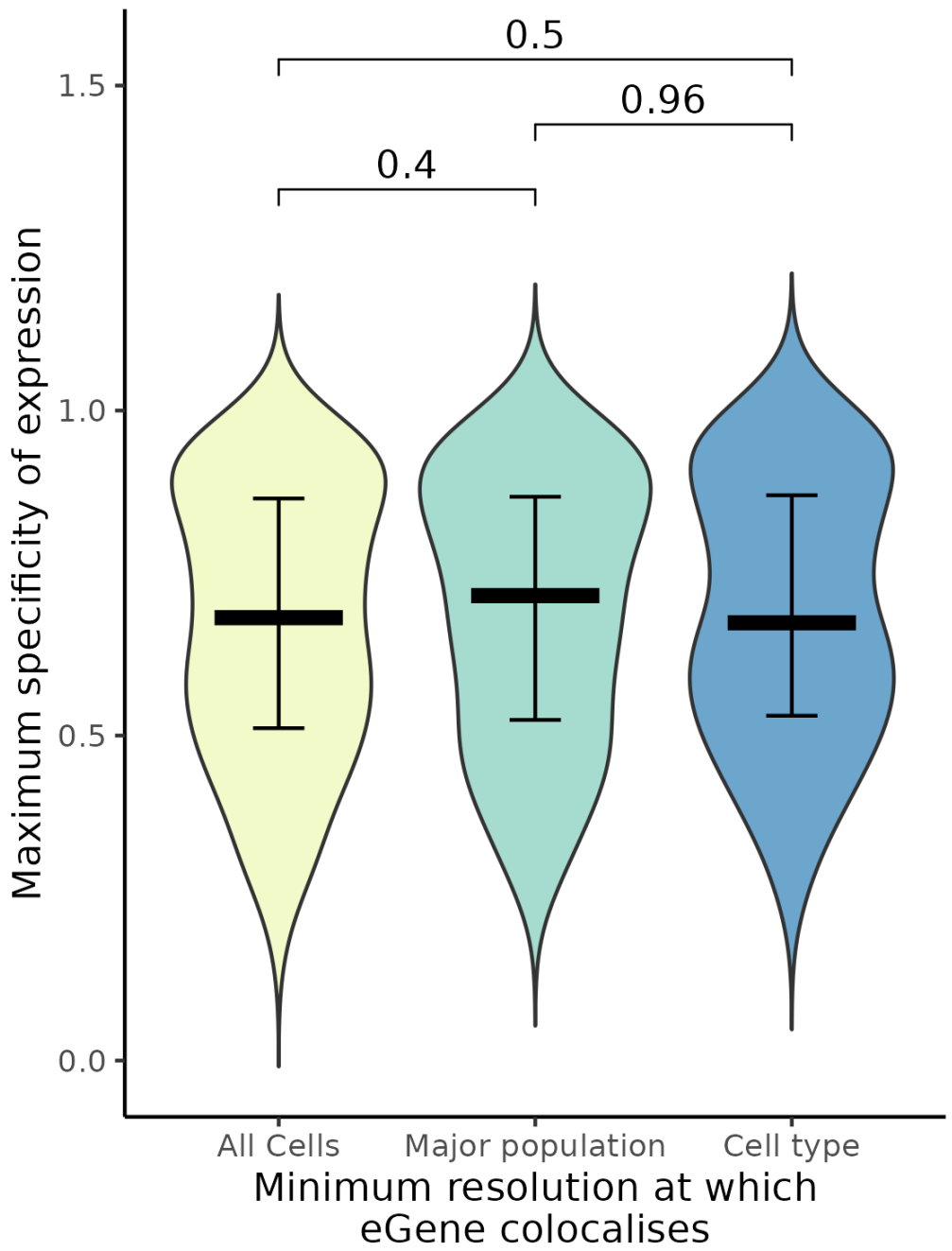

Supplementary Figure 4: **Disease effector genes identified at high resolutions are not more specifically expressed than those at lower resolutions.** Disease effector genes were grouped by the minimum resolution at which their colocalisation was detected (x-axis), as in Figure 2d. The specificity of effector gene expression in each cell type was then calculated using CELLEX[104], and the maximum specificity of effector gene expression is presented (y-axis). Horiztonal lines indicate the 25th, 50th and 75th percentile. P-values are calculated by unpaired Wilcoxon.

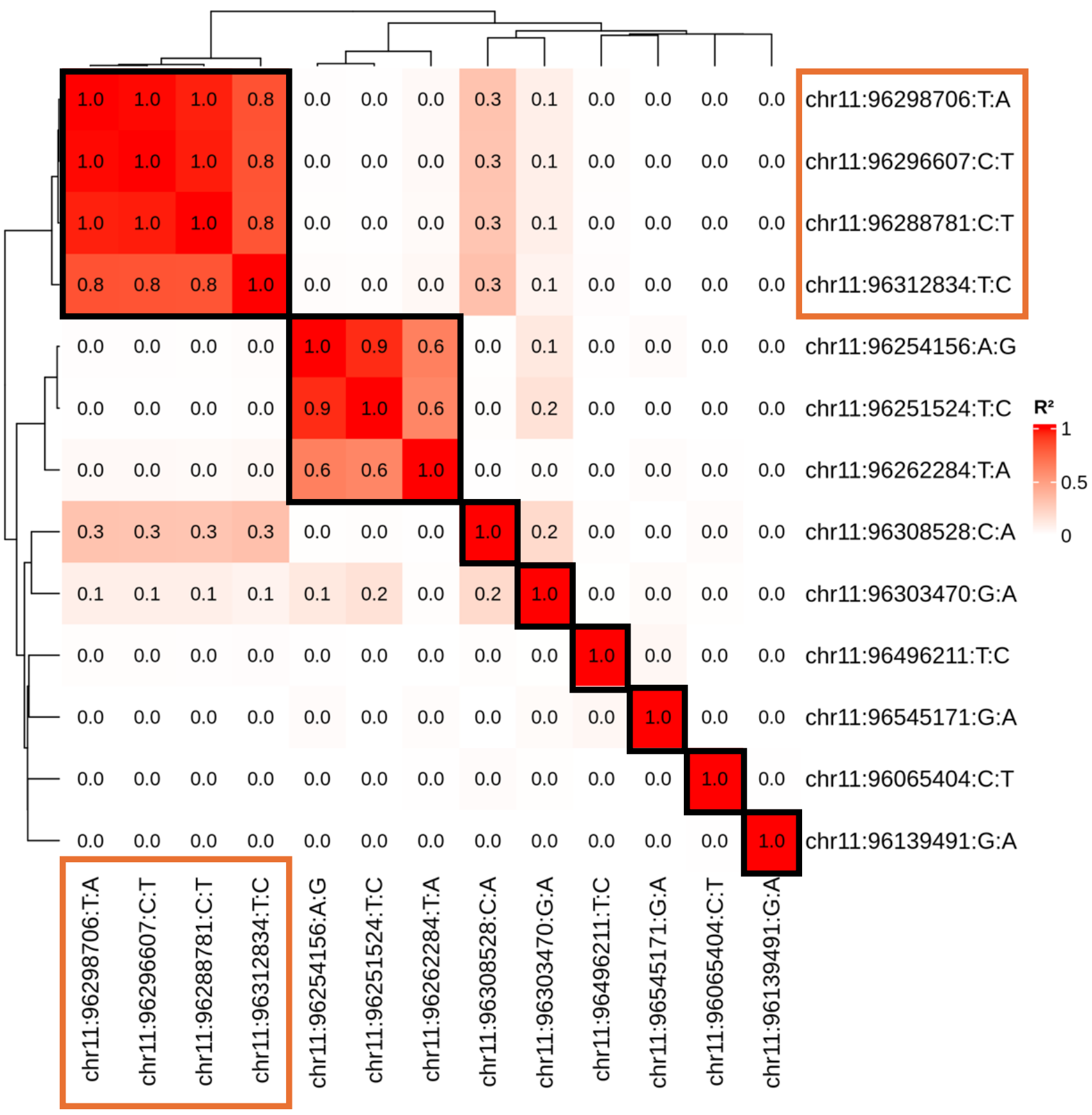

Supplementary Figure 5: **Independence of eQTLs for *MAML2* dysregulation.** Heatmap depicting the pairwise linkage disequilibrium (r^2) between lead variants from each condition with a significant eQTL. Clumped effects are indicated by black boxes around pairise tests. Lead variants that colocalise with IBD are outlined by an orange box.

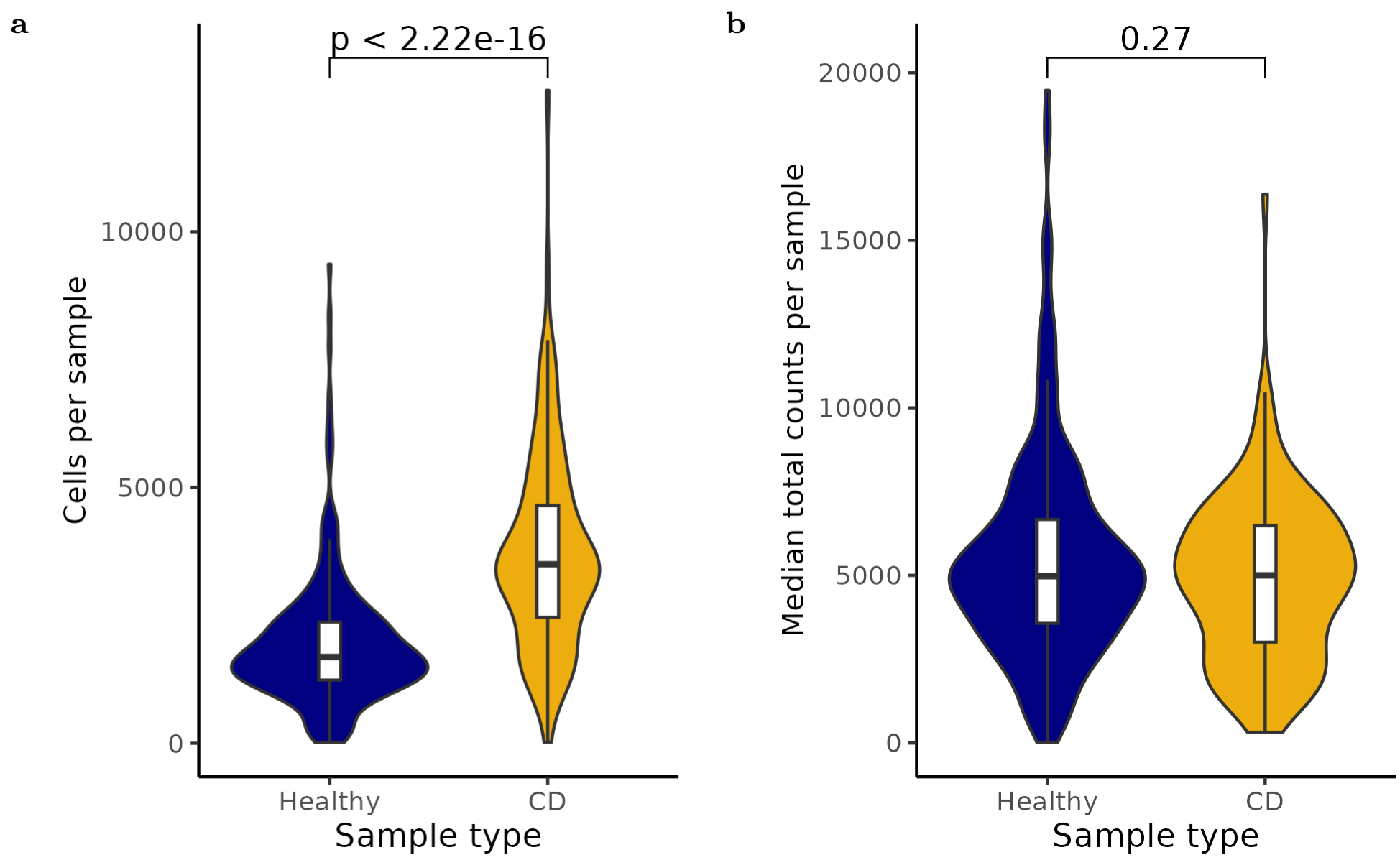

Supplementary Figure 6: **The increased number of cells sequenced for CD samples does not confound total expression counts** The number of cells (a) and median expression counts (b) of healthy and CD TI samples in the 'atlasing' dataset. P-values calculated by unpaired wilcoxon test. Boxplots indicate the 25th, 50th and 75th percentiles and whiskers extend to 1.5 times the interquartile range.

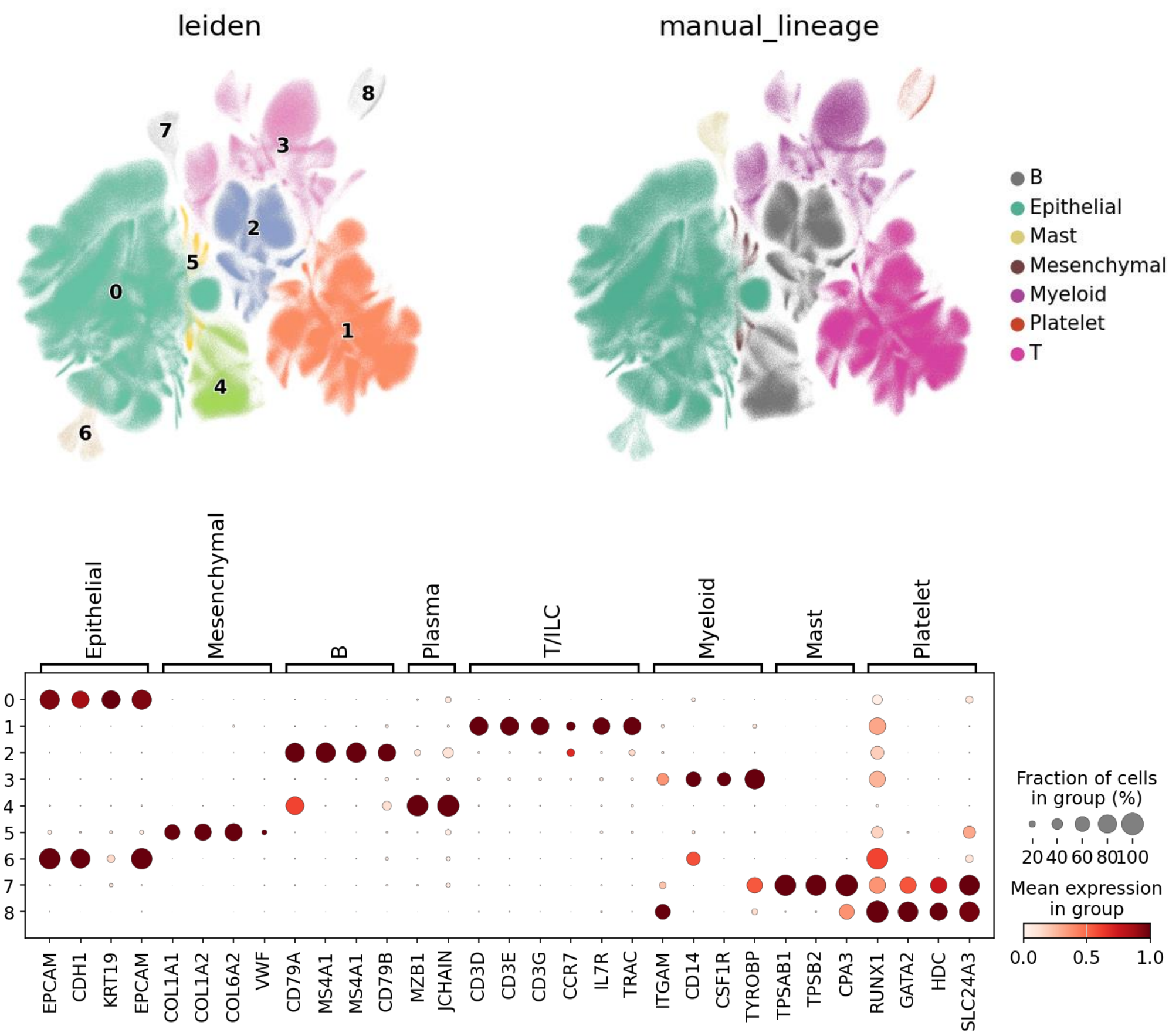

Supplementary Figure 7: **Identification of major lineages following round 1 of quality control and integration.** Leiden clusters identified by clustering at resolution of 0.03 (upper left) following the first round of quality control and integration (see Supplementary Figure S1). Expression of canonical marker genes across leiden clusters, markers derived from literature (bottom). Annotation of the major lineages after grouping (upper right).

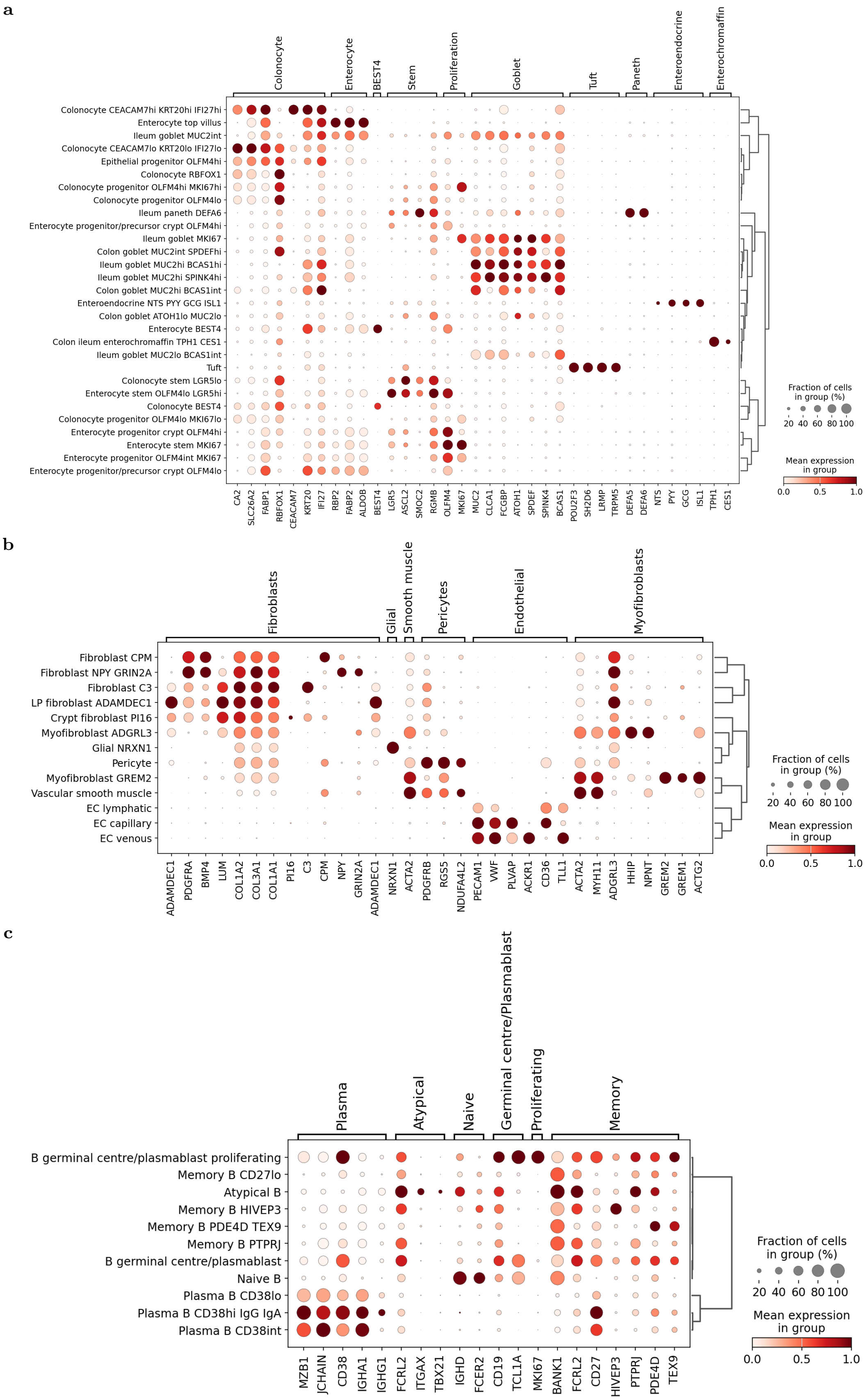

Supplementary Figure 8

d

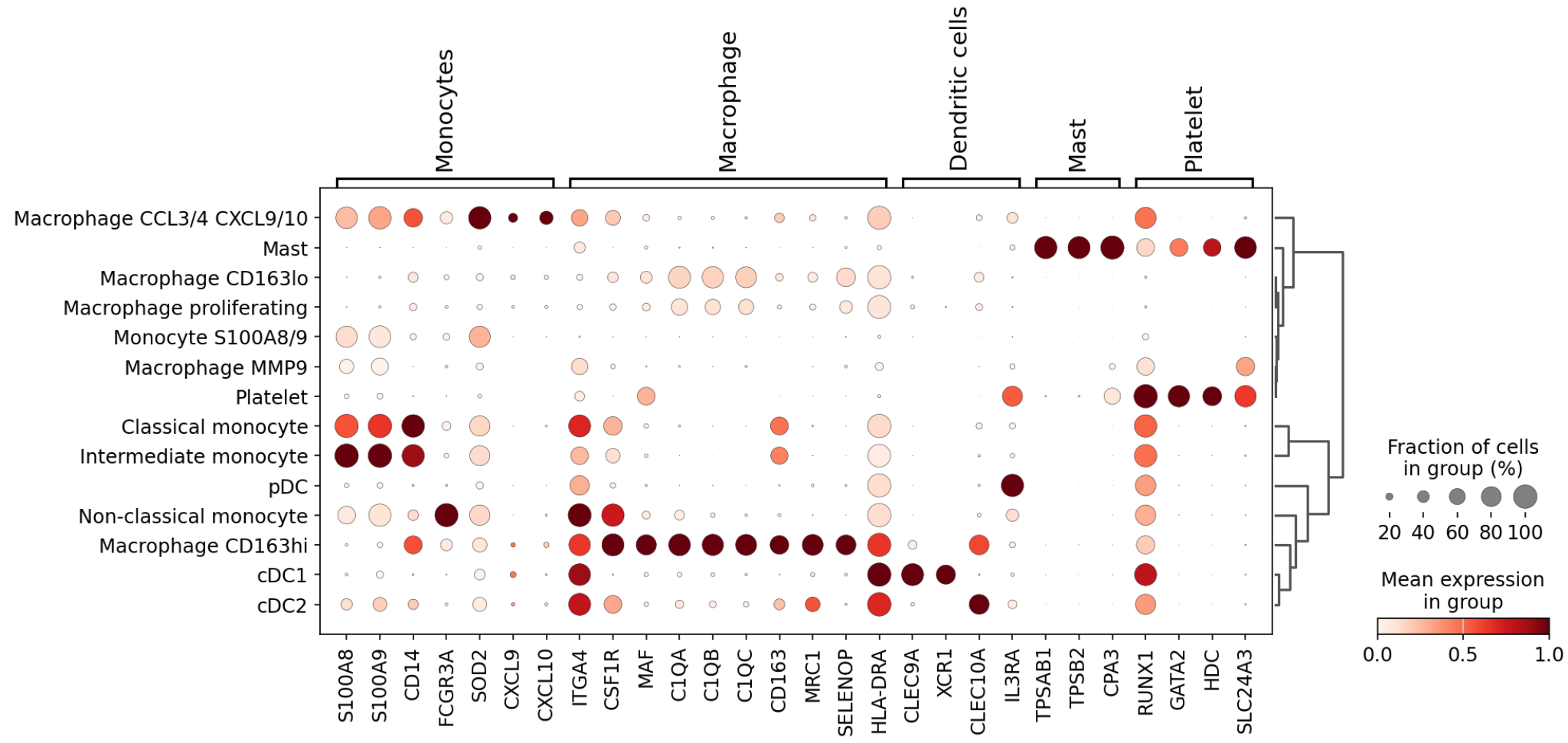

e

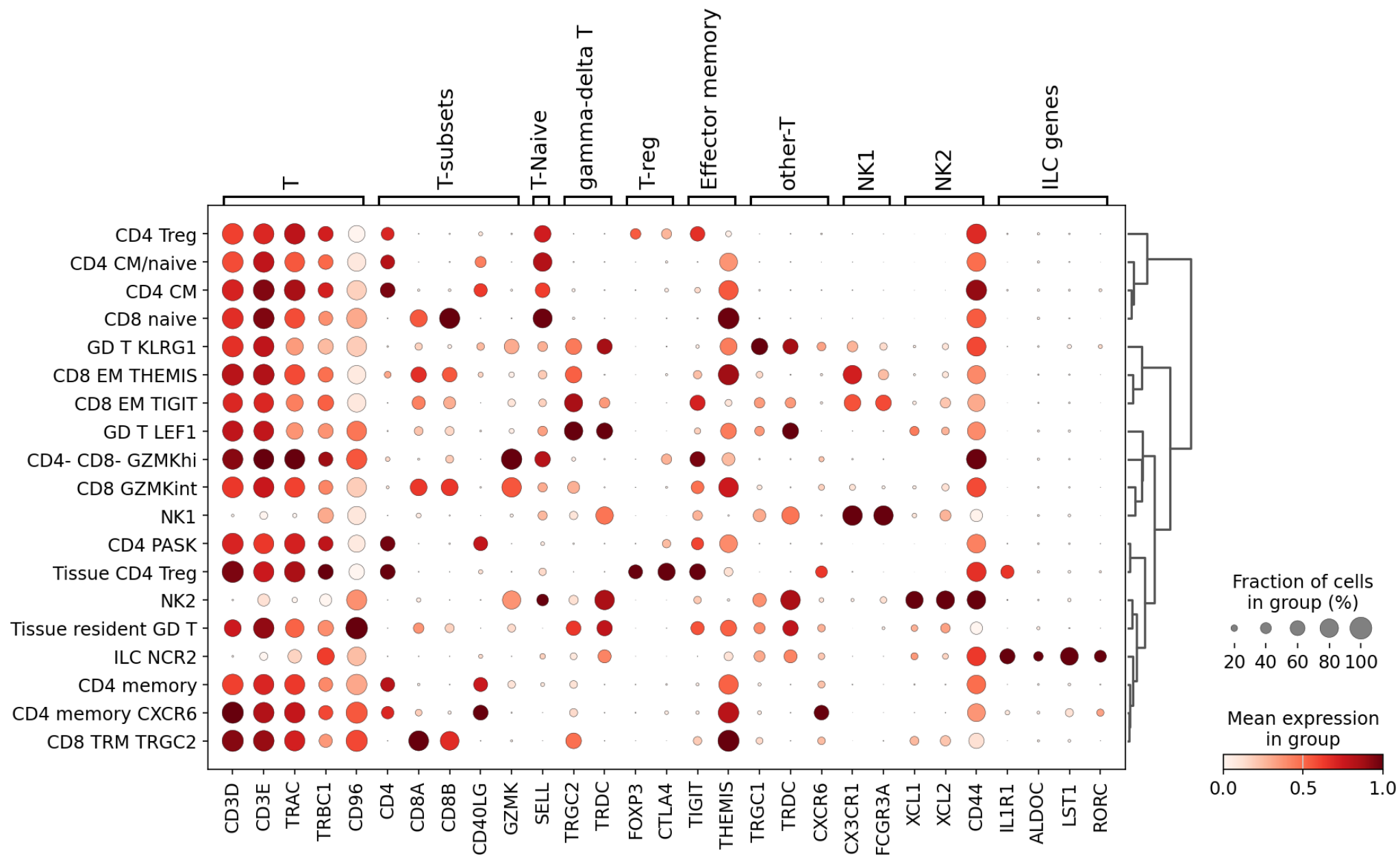

Supplementary Figure 8: **Marker gene expression for the cellular annotations.** a-e) Dotplots for the expression of demarcating and discriminatory genes used to annotate clusters from each lineage, defined as in Supplementary Figure S7.

3 Supplementary Note

3.1 Inferring the capture of our eQTLs in other studies

To test how well our regulatory associations are captured by other studies, we computed the $\pi1$ statistic - an estimate of the fraction of associations from one set of summary statistics also found in a second by enrichment of low p-values (Storey and Tibshirani 2003) - between our eQTLs and each GTEx v8 tissue and sc-eQTLs from peripheral blood cell types of Yazar et al., (2022). We envisaged assessing how well our associations are captured by external datasets in two ways; i) Testing the maximum $\pi1$ for each of our cell types across external datasets, and ii) Testing whether pairwise $\pi1$ values followed expectation, assuming more biologically similar datasets would show a greater similarity than biologically dissimilar comparisons. Boxplots indicate the 25th, 50th and 75th percentiles and whiskers extend to 1.5 times the interquartile range.

3.1.1 Capture of our eQTLs in GTEx

We first calculated, for each of our annotations, the maximum $\pi1$ observed with any GTEx tissue (max-$\pi1$, Supplementary Fig S9a), and found most annotations to show high max-$\pi1$ with at least one tissue (median max-$\pi1$=0.77). There were 5 annotations with max-$\pi1 < 0.25$; Tissue Memory B CD27Lo from the rectum (max-$\pi1$=0.21), Colonocyte from the TI (max-$\pi1$=0.08), Fibroblast CPM+ from the TI (max-$\pi1$=0.15), proliferating Tissue Macrophages from across sites (max-$\pi1$=0.03) and CD163+ tissue macrophages from the rectum (max-$\pi1$=0.17). However, only a maximum of 15 eQTLs were identified within each of these annotations, and so these values are likely unreliable. This evidence therefore broadly supports many of our eQTLs to be well supported by GTEx eQTLs.

The resolution of our cellular annotations which is most analogous to bulk RNAseq is the 'All Cells' resolution. To test whether variation in $\pi1$ values followed biological expectation, we therefore compared the $\pi1$ values for eQTLs detected in the 'All Cells' grouping of each site with each GTEx tissue (Supplementary Fig S9b). The GTEx tissue where we observed the greatest $\pi1$ value for our blood 'All Cells' eQTLs was the blood collection of GTEx ($\pi1$=0.79). In the TI, the greatest $\pi1$ value for 'All Cells' eQTLs was also the blood of GTEx ($\pi1$=0.62), closely followed by the thyroid ($\pi1$=0.61) and the transverse colon ($\pi1$=0.59). The greatest $\pi1$ for blood could be due to our collection of TI samples from CD patients, which show an enrichment of immune populations (Krzak et al. 2024), while relatively high $\pi1$ values with the thyroid could be due to its large proportion of epithelial cells, and transverse colon due to its anatomical proximity and biological similarity.

Interestingly, the small intestine eQTLs of GTEx, which is also collected from the terminal ileum, showed a more modest $\pi1$ value for our TI 'All Cells' eQTLs ($\pi1$=0.51). This may be indicative of both sample and cohort level differences between our study and GTEx - we collect both healthy and diseased samples from live individuals while GTEx is based on postmortem samples from healthy individuals only, and we optimise digestion steps to preserve epithelial cells (Krzak et al. 2024), while GTEx preferentially collect from 'Peyer's patches' - lymphoid nodules likely devoid of epithelial cells. The high $\pi1$ value with the transverse colon, which is not subset for Peyer's patches, supports this relatively low $\pi1$ with the small intestine of GTEx to be indicative of sampling differences.

For the rectum 'All Cells' eQTLs, the greatest $\pi1$ value was observed with the thyroid collection of GTEx ($\pi1$=0.604), closely followed by the transverse colon ($\pi1$=0.603). Similar to the TI, the $\pi1$ value of the most anatomically similar collection from GTEx, the sigmoid colon, was relatively low ($\pi1$=0.51). However, GTEx reports the sigmoid colon collection to include muscularis only, discarding mucosa and therefore the vast majority of epithelial and immune populations. This sampling difference likely results in the transverse colon being the most anatomically similar site, with which it shows a relatively high $\pi1$ value. Together, this therefore indicates many of our associations are well supported by eQTLs in at least one GTEx tissue, and for this to broadly follow the biological similarity of the pairwise comparisons at the 'All Cells' level.

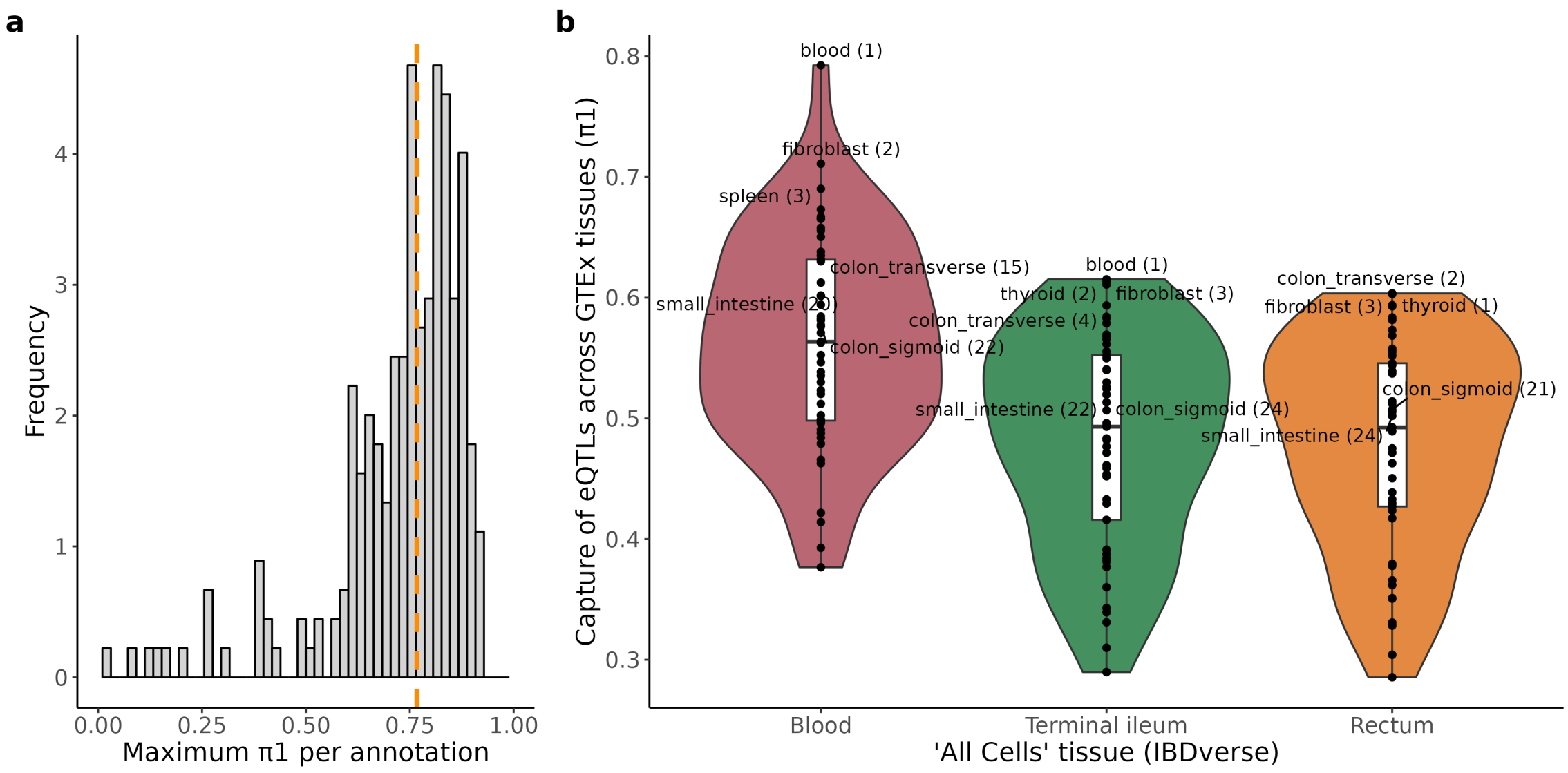

Supplementary Figure 9: **Capture of our eQTLs in GTEx v8.** a) The maximum $\pi1$ statistic (Storey and Tibshirani 2003)) for eQTLs detected in each of our cellular annotations across GTEx tissues. The vertical orange line indicates the median maximum $\pi1$ observed (0.77). (b) Capture of eQTLs detected in the 'All Cells' grouping of each anatomical site of our dataset with each GTEx tissue. The tissues with the top three highest $\pi1$ statistics and/or intestinal datasets are labelled, along with the rank for their $\pi1$ statistic in brackets.

3.1.2 Capture of our eQTLs in Yazar *et al.,* (2022)

Similar to our comparison with GTEx, after computing $\pi1$ statistics between all our annotations with sc-eQTLs from PBMCs of Yazar et al., we identified the cell type with the maximum $\pi1$ for each of our annotations, Supplementary Fig S10a. As expected, the maximum $\pi1$ of eQTLs in immune major populations were generally greater than non-immune major populations (median max-$\pi1$ range=0.71-0.93 compared with 0.62-0.7 respectively). This was particularly strong for eQTLs detected in T cells (median max-$\pi1$=0.93), and greatest for those from *CD8*$^+$ *TIGIT*$^+$ effector memory T cells from the blood ($\pi1$=0.95). The lowest max-$\pi1$ was found for proliferating Macrophages eQTLs ($\pi1$=0.009) and *CCL3/CCL4 CXCL9/10* Macrophages eQTLs ($\pi1$=0.01), though based on very few associations (< 15 each).

To further test whether the capture of our eQTLs in this external dataset was consistent with the biological similarity of comparisons, we grouped Yazar et al., cell types into broad major populations analogous to our own based on nomenclature (Methods) and tested how often the major populations matched for our immune cell-types and the cell-type that best captured our eQTLs, Supplementary Figure 17b. Overall, major populations matched 76.3% of time, significantly better than might be expected by chance (Cohen's kappa test=0.6, p=2.1e-8). In addition, major populations were particularly well-matched for eQTLs detected in our T-cell populations (F1 score=0.84), which form the majority of both our own immune and Yazar et al., (2022) cell-types (Supplementary Fig S10c). Together, this supports a high level of support for eQTLs detected within our immune cell types, and for this to also be consistent with the inferred biological similarity of comparisons.

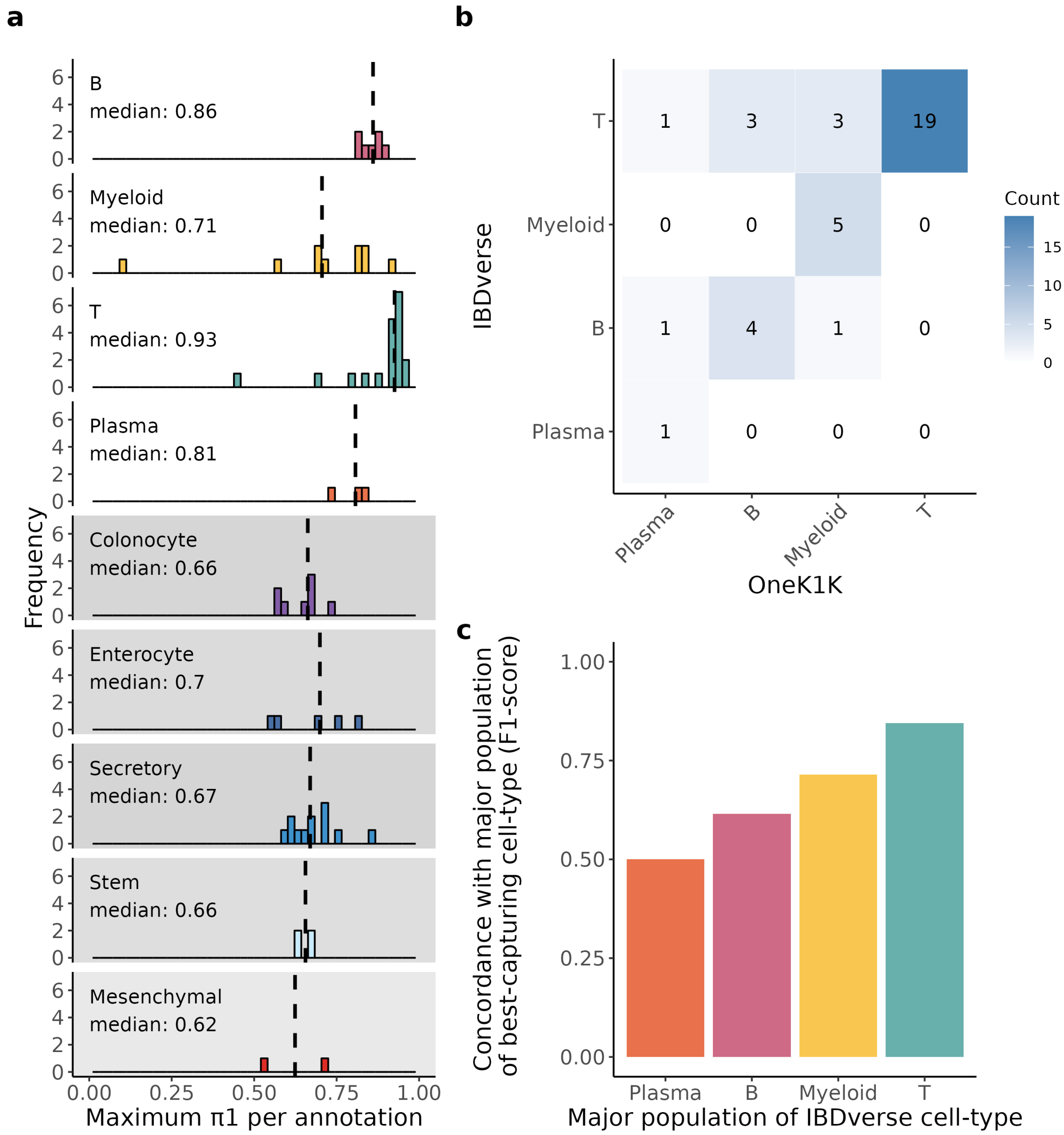

Supplementary Figure 10: **Capture of our eQTLs in Yazar *et al.*, (2022).** a) The maximum $\pi1$ for eQTLs of each of our cell types with cell types from Yazar et al., (2022), grouped by major population. The median $\pi1$ is shown by the vertical line. The white and grey backgrounds indicate immune and non-immune populations respectively. (b) Confusion matrix for matching of major population annotations of our cell types and the cell type from Yazar et al., (2022) with the greatest $\pi1$ statistic. Cohen's kappa test=0.6, p=2.1e-8. (c) F1-score for the accuracy of major population matching from (b).

3.2 Fine mapping the *RNF14* locus

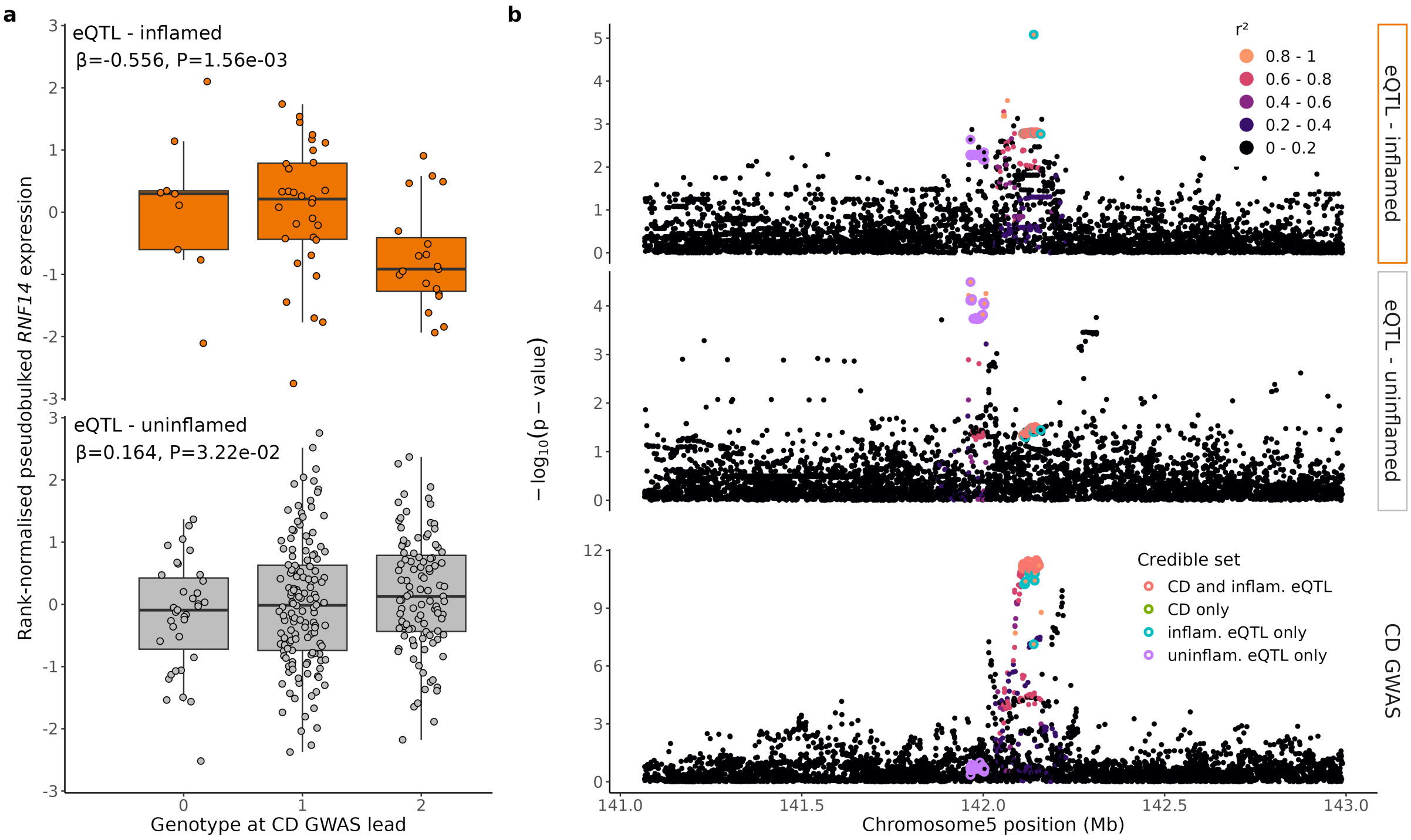

Supplementary Figure 11: **The colocalising eQTL for *RNF14* in Enterocytes is specific to inflamed samples**. (a) The regulatory association of chr5:142146492:C:A, the variant with the greatest posterior probability for CD susceptibility in the region (posterior inclusion probability=0.033, Methods), in Enterocyte top villus cells from inflamed individuals (n=58, above) and uninflamed individuals (n=262, below). Boxplots indicate the 25th, 50th and 75th percentiles and whiskers extend to 1.5 times the interquartile range. (b) The regional association of variants for the main eQTL effect of *RNF14* in Enterocyte top villus cells from inflamed samples (top), uninflamed individuals (middle) and CD susceptibility (bottom). Points are coloured by their linkage disequilibrium with the lead variant from each dataset (r^2) and outlined by their inclusion in the 95% credible set for each phenotype.

To validate the shared genetic association of an inflammation-dependent ieQTL on *RNF14* in Enterocyte top villus cells and CD susceptibility, we first performed fine-mapping of the CD association in the region (see Methods) and identified a single 95% credible set (CS) of variants (n=53). As the original ieQTL was detected by interaction of genotype with inflammation status in Enterocyte top villus cells, we then tested for main (G) eQTL effects of these variants with *RNF14* expression level in this population, testing within inflamed samples (SES-CD score $>$ 3, n=58) and uninflamed samples (SES-CD $<$ 3, n=262) separately.

In inflamed samples, the variant from the CD CS with the largest PIP, chr5:142146492:C:A, was the variant with the smallest nominal p-value for the eQTL, with the negative effect on gene-expression remaining significant after FDR correction across these 53 variants (CD GWAS PIP=0.033, β-eQTL=-0.556, eQTL nominal p=1.56e-3, eQTL FDR=1.70e-03) (Supplementary Figure S11a). In contrast, in uninflamed samples, this variant was associated with a marginally significant positive effect on gene expression (β-eQTL=0.164, eQTL nominal p=3.22e-2).

To better characterise the *RNF14* eQTL signals in these subsets, we then performed conventional cis-eQTL mapping, testing for a main effect (G) of all variants within 1Mb of the transcription start site of *RNF14* (MAF>0.05), and subjected these to fine-mapping (Supplementary Figure S11b). Unfortunately, a 95% credible set of variants could not be identified for either eQTL effect using SuSiE, likely due to the reduction in statistical power by focusing on a smaller number of samples. Instead, we therefore computed an approximate credible set of variants for the eQTL associations using the Probabilistic Identification of Causal SNPs (PICS) algorithm (Farh et al., 2014, Nature), which incorporates the haplotype structure to estimate the posterior probability that variants are within the credible set based on the in-sample LD with the lead variant.

PICS fine mapping identified a non-overlapping set of 64 and 68 variants for eQTLs from inflamed and uninflamed samples respectively. 52 of the 53 CD CS variants were included in the approximate credible set from

the inflamed eQTL, but none were included in the eQTL from uninflamed samples. While we acknowledge these approximate credible sets are not as statistically robust as those computed with SuSiE, the substantial overlap of the CD CS with the eQTL CS from inflamed samples is consistent with the regulatory signal being shared with that of CD susceptibility. In addition, the exclusivity of these variants further supports this regulatory effect to be specific to inflamed samples only, in line with our findings from the original ieQTL.
