# Supplementary material for: Cell-type-resolved genetic variation shapes inflammatory bowel disease risk
Source: Nature. 2026 Jun 3;656(8126):129–39. doi: 10.1038/s41586-026-10627-z (PMC13441992; doi:10.1038/s41586-026-10627-z)
Supplement: Supplementary file 2 [file 41586_2026_10627_MOESM2_ESM.pdf]

nature portfolio

Corresponding author(s): Carl A Anderson

Last updated by author(s): Jul 3, 2025

Reporting Summary

Nature Portfolio wishes to improve the reproducibility of the work that we publish. This form provides structure for consistency and transparency in reporting. For further information on Nature Portfolio policies, see our Editorial Policies and the Editorial Policy Checklist.

Statistics

For all statistical analyses, confirm that the following items are present in the figure legend, table legend, main text, or Methods section.

n/a	Confirmed	
☐	☒	The exact sample size (*n*) for each experimental group/condition, given as a discrete number and unit of measurement
☐	☒	A statement on whether measurements were taken from distinct samples or whether the same sample was measured repeatedly
☐	☒	The statistical test(s) used AND whether they are one- or two-sided<br>*Only common tests should be described solely by name; describe more complex techniques in the Methods section.*
☐	☒	A description of all covariates tested
☐	☒	A description of any assumptions or corrections, such as tests of normality and adjustment for multiple comparisons
☐	☒	A full description of the statistical parameters including central tendency (e.g. means) or other basic estimates (e.g. regression coefficient) AND variation (e.g. standard deviation) or associated estimates of uncertainty (e.g. confidence intervals)
☐	☒	For null hypothesis testing, the test statistic (e.g. *F*, *t*, *r*) with confidence intervals, effect sizes, degrees of freedom and *P* value noted<br>*Give P values as exact values whenever suitable.*
☐	☒	For Bayesian analysis, information on the choice of priors and Markov chain Monte Carlo settings
☒	☐	For hierarchical and complex designs, identification of the appropriate level for tests and full reporting of outcomes
☐	☒	Estimates of effect sizes (e.g. Cohen's *d*, Pearson's *r*), indicating how they were calculated

Our web collection on statistics for biologists contains articles on many of the points above.

Software and code

Policy information about availability of computer code

Data collection	CellRanger v7.2.0 was used to demultiplex reads, align reads to GRCh38 with Ensembl version 93 transcript definitions (GRCh38-3.0.0 reference file distributed by 10X Genomics), and generate cell by gene count matrices. CellBender v2.1 was then applied to identify droplets containing cells and adjust the raw counts matrix for background ambient transcript contamination.<br><br>Genotype data was sequenced on an Applied Biosystems UK Biobank Axiom Array in five batches of samples by YourGene Health. Variants were called by YourGeneHealth in a joint analysis across all batches. Imputation was performed with the Michigan Imputation Server v1 using the TOPMed reference panel.
Data analysis	Packages used within data generation pipelines: Data handling in python: Pandas v2.2.2, management of single-cell RNAseq data: scanpy(v1.9.6, Identification of droplets: CellBender v2.1, Estimation of the number of droplets: DropletUtils v1.9.16, Removal of multiplets: scrublet v0.2.1, Filtering, normalisation, highly variable gene selection, calculation of principal components: scanpy v1.9.6, Removal of batch effects: scVI v1.1.2 and torch v1.1.2, Calculation of clusters: Leiden clustering algorithm v0.8.3, Prediction of cluster identity with single layer dense neural network: keras v2.4.3, Identification of gene markers: scanpy v1.9.6, Calculation of UMAP projection: scanpy v1.9.6, Auto-annotation of cells: Celltypist v1.6.2, eQTL analysis: TensorQTL v1.0.9, Colocalisation analysis: coloc v5.2.2 (Github: “compare_datasets” branch for interaction colocs), Gene set enrichment analysis: FGSEA v1.2.8, Identification of specifically expressed genes: CELLEX v1.2.1, Genotype quality control: plink v1.90b6.27, Genotype ancestry prediction KING v2.3.2, Computation of linkage disequilibrium between leads: plink v1.90b6.27.

For manuscripts utilizing custom algorithms or software that are central to the research but not yet described in published literature, software must be made available to editors and reviewers. We strongly encourage code deposition in a community repository (e.g. GitHub). See the Nature Portfolio guidelines for submitting code & software for further information.

Data

Policy information about availability of data

All manuscripts must include a data availability statement. This statement should provide the following information, where applicable:

- Accession codes, unique identifiers, or web links for publicly available datasets
- A description of any restrictions on data availability
- For clinical datasets or third party data, please ensure that the statement adheres to our policy

The raw scRNA-seq reads and genotype data have been deposited in the European Genome-phenome Archive under the accession numbers that will be made available upon publication. Processed expression data (both from the atlasing and eQTL mapping cohort), the CellTypist prediction model, imputed genotype data and summary statistics from all eQTL, ieQTL and colocalisation analysis are available at https://www.ibdverse.info/.

Research involving human participants, their data, or biological material

Policy information about studies with human participants or human data. See also policy information about sex, gender (identity/presentation), and sexual orientation and race, ethnicity and racism.

Reporting on sex and gender	Sex was self-reported by all participants via a questionnaire and confirmed via genotype data (presence of heterozygous X alleles indicating female) as well as gene expression data (expression of XIST indicating female, expression of Y chromosome genes indicating male).<br>Interaction eQTL analyses with sex as the interaction variable (coded as 0 or 1) were performed on samples after sex confirmation.
Reporting on race, ethnicity, or other socially relevant groupings	Ethnicity was self-reported by all participants via a questionnaire. Ethnicity assignments were then validated by comparing self-reported data with continental population ancestry, as determined through principal component analysis of genotype data using. Individuals of non-European ancestry were excluded from the study.
Population characteristics	A total of 732 samples were collected from 296 healthy individuals and 125 Crohn's disease patients, comprising 201 males and 220 females. The CD patients were classified based on the endoscopic severity of the disease, using the TI-SES-CD score applied to the terminal ileal segment. All participants were of European ancestry and aged between 18 and 70 years.
Recruitment	Individuals undergoing routine endoscopic assessment were recruited at Addenbrooke's hospital, Cambridge, UK. All control participants were undergoing endoscopic assessment or surveillance for healthy and non-cancer related reasons (e.g., history of iron deficiency anaemia, family history of colorectal cancer). Healthy participants did not have macroscopic evidence of intestinal inflammation, a personal history of cancer, and were not in receipt of corticosteroids or any other immune modulating therapy.
Ethics oversight	This study was approved by the National Health Service (NHS) Research Ethics Committee (Cambridge South, REC ID 17/EE/0338). Written informed consent was given by all participants.

Note that full information on the approval of the study protocol must also be provided in the manuscript.

Field-specific reporting

Please select the one below that is the best fit for your research. If you are not sure, read the appropriate sections before making your selection.

☒ Life sciences ☐ Behavioural & social sciences ☐ Ecological, evolutionary & environmental sciences

For a reference copy of the document with all sections, see nature.com/documents/nr-reporting-summary-flat.pdf

Life sciences study design

All studies must disclose on these points even when the disclosure is negative.

Sample size	Our study analyzed over 4 million cells derived from 362 terminal ileal biopsies, 275 rectal biopsies and 95 blood samples. Sample collection in our study was based on donor availability during the designated sampling period, and no statistical methods were used to pre-determine the sample size.
Data exclusions	Patients: To minimize confounding factors, we excluded patients who were using probiotics or antibiotics. Additionally, individuals of non-European ancestry and those outside the age range of 18 to 70 years were not included in the study.<br>Data: Data were filtered using stringent quality control metrics, which involved the removal of cells which were identified as doublets or low-quality as well as genotypes which were outliers in terms of Hardy-Weinberg equilibrium or missingness (see Methods for details).
Replication	This work was conducted with scope for discovery of novel genetic associations. Unfortunately, there currently is not an equivalent cohort of genotyped scRNAseq data from IBD patient blood, healthy / patient TI or healthy rectum data. Replication of eQTLs was therefore not possible. All reported findings were significant at multiple testing correcting thresholds below 0.05, with eQTL test significance determined by 1000 permutations within genes and additional corrections for the number of tested genes.

Replication of colocalisations was performed by comparison with the Open Targets Genetics database of colocalisations.

Randomization

There was no random allocation of samples or participants to groups in this study as comparisons were made between individuals based on their genotype data. eQTL testing was performed on all samples that contributed sufficient numbers of cells to each annotation, which is dependent on the number of cells that passed quality control for each sample, the number of samples collected per tissue and the rarity of each cell-type. In our models, we incorporated 5 genotype PCs to account for population structure, in addition to a number of expression PCs that account for phenotypical differences such as age and sex.

Blinding

Samples were anonymized using unique ID numbers prior to data analysis. The target number of cells to be sequenced from diseased and healthy samples was different (6000 and 3000 respectively), and so complete blinding was not possible during the sample processing stage however the derivation of cells from healthy or diseased samples was not considered when performing cell or cluster quality control. Downstream analyses were performed blinded with no knowledge of the individuals who donated the samples.

Reporting for specific materials, systems and methods

We require information from authors about some types of materials, experimental systems and methods used in many studies. Here, indicate whether each material, system or method listed is relevant to your study. If you are not sure if a list item applies to your research, read the appropriate section before selecting a response.

Materials & experimental systems

n/a	Involved in the study	
☒	☐	Antibodies
☒	☐	Eukaryotic cell lines
☒	☐	Palaeontology and archaeology
☒	☐	Animals and other organisms
☒	☐	Clinical data
☒	☐	Dual use research of concern
☒	☐	Plants

Methods

n/a	Involved in the study	
☒	☐	ChIP-seq
☒	☐	Flow cytometry
☒	☐	MRI-based neuroimaging

Plants

Seed stocks

Report on the source of all seed stocks or other plant material used. If applicable, state the seed stock centre and catalogue number. If plant specimens were collected from the field, describe the collection location, date and sampling procedures.

Novel plant genotypes

Describe the methods by which all novel plant genotypes were produced. This includes those generated by transgenic approaches, gene editing, chemical/radiation-based mutagenesis and hybridization. For transgenic lines, describe the transformation method, the number of independent lines analyzed and the generation upon which experiments were performed. For gene-edited lines, describe the editor used, the endogenous sequence targeted for editing, the targeting guide RNA sequence (if applicable) and how the editor was applied.

Authentication

Describe any authentication procedures for each seed stock used or novel genotype generated. Describe any experiments used to assess the effect of a mutation and, where applicable, how potential secondary effects (e.g. second site T-DNA insertions, mosiacism, off-target gene editing) were examined.
