# Supplementary material for: Cell-type-resolved genetic variation shapes inflammatory bowel disease risk
Source: Nature. 2026 Jun 3;656(8126):129–39. doi: 10.1038/s41586-026-10627-z (PMC13441992; doi:10.1038/s41586-026-10627-z)
Supplement: Supplementary file 3 [file 41586_2026_10627_MOESM6_ESM.pdf]

nature portfolio

Peer Review File

Cell type-resolved genetic variation shapes inflammatory bowel disease risk

Corresponding Author: Dr Carl Anderson

This file contains all reviewer reports in order by version, followed by all author rebuttals in order by version.

Version 0:

Reviewer comments:

Referee #1

(Remarks to the Author)
This manuscript tests the idea that bulk sequencing analysis can miss certain traits whose effects are at the level of the cell type. Their starting framework was that genome wide analysis (GWAS) has revealed that single nucleotide polymorphisms (SNPs) associated with IBD risk are more likely to reside within gene enhancers while regulatory effects like eQTLs shared across multiple cell types are more likely to reside in promoters. The authors use single cell RNA sequencing data combined with genotyping from each patient to determine whether there are additional eQTLs at the cell type level and what the location was on the chromosome. They built the IBDverse, a collection of about 2 million cells from about 400 patients (about one-third having IBD), including blood cells along with cells isolated from ileal and rectal mucosal biopsies and showed that additional genes exist at the cell type level that have eQTLs farther away from the promoter than those previously detected. About 150 of these eQTLs mapped to the same region as known IBD risk genes (about half of the 300 IBD risk loci). Additional analysis revealed that inflammation during IBD affected how these eQTLs impacted the effector gene (eGenes) expression. The conclusion being that many IBD risk loci at enhancers are eQTLs whose effects can only be detected by single cell analysis.

The approach is valid, the work is significant, and the quality of the data is high. Although small effect genes / products can have major influence on biology, my first major point would be that it is still unclear the impact some of these small effects at the cellular level would have on the biology of the disease, and how much weight these observations carry overall in biological significance. Especially when considering that mRNA levels are not always good indicators of protein levels and activity. A major technical point that directly ties to the first concern is that despite the high number of cells used in the study, the statistical power of single cell data is so limited by the few transcript copies per gene that make their way into the library for each cell that this sparsity (some genes might only have 10 or 5 or even 1 transcript copy per cell) makes it difficult to appreciate how truly robust these analysis are, especially considering it still seems questionable which chromosome with the SNP served as the template for those transcripts and so on. It seems the study relied on statistical power from the number of cells, which allowed for the transcripts of that subpopulation of cell types to be analyzed in a pseudobulk manner, in my mind calling into question the single cell aspect of the study. Thus, it is also still a question of biological significance or technical that eQTLs are enriched in single cell analysis. I think more elaboration on how the statistical framework dealt with these biological and technical factors is needed to help bolster the claims, along with some type of validation and experimental study to show how these eQTL influence or produce a phenotype. The work is somewhat novel, but lacks experimental validation, and relies too heavily on one set of methodology, and the text is difficult to read in the parts of the manuscript that tied the eQTLs to molecular genetic behavior. The study leans heavily on existing literature to establish links between candidate genes and IBD without offering new biological insights.

Comments/Questions:

1) Several papers published have used genetic and single cell level analysis, but not in IBD. The following are references of previous work in the field; PMID: 35389779, 40112817, 35328055. Another lab group appears to have also used similar technology using bulk RNAseq and analysis on an IBD cohort with similar sample size and results PMID: 33602935. This current manuscript used a large number of single cell, providing more than just incremental advances.

2) The presentation was a bit challenging, and I had a difficult time reading through some of the text in the Results section and I didn’t fully understand what was implied in certain instances. Given the descriptive nature of the study, I also thought

there were several assertions made about the results regarding the impact on molecular genetic phenomena that were not fully supported by the data or clearly conveyed in the text. For example, it is stated: Line 168 “These findings demonstrate that genetic variation contributes to inter-individual differences in cellular responses to inflammation, and that interaction eQTL mapping using scRNA-seq data can identify context-dependent regulatory mechanisms relevant to disease.”

3) More work needs to be done to merge the molecular genetic concepts with the more abstract and statistically based associations of eQTL and GWAS signals have on gene expression in the main text. For example, at Line 148 “Because GWAS loci are also enriched in enhancers rather than promoters, these findings suggest that eQTLs detected at higher transcriptional resolution may more closely align with the regulatory architecture of complex trait associations.”

4) What about chromatin structure or accessibility at these locations the authors identified? Or transcription factor site binding? There were also several words used like “colocalized” that had an unclear connotation. Sometimes it seemed to be used in a physical sense of chromosomal location and other times in a more abstract, association-like context when tied to a phenotype.

5) Use of the word effector cell types lines 242 is not clear how they can be labeled as such based on association data, then also mentions effector genes in line 245....so the word effector is a bit vague

6) How can this statement about expression regulation be made based on association data? We identified 84,376 eQTLs regulating the expression of 20,389 of the 24,761 genes tested 118 (82.2%)

7) The description of the patient recruitment numbers is not clear, nor is which biopsies, blood, etc came from which patients. The Introduction mentions UC, part of the Results mention UC, but there is no mention of UC recruitment or sample collection. Were the rectal biopsies collected from different patients than the ileal biopsies? If so, is this accounted for in the analysis? The descriptions of the IBD phenotypes/metadata are not sufficiently detailed.

8) Why was there twice the number of cells encapsulated for scRNA-seq for IBD patients than for controls? How was the difference in sequencing depth across these samples accounted for? Additionally, given these differing targets, it would be important to report on the number of cells and the median number of genes per cell before and after quality control across both IBD and non-IBD samples

9) Line 525 reports “50,0000 reads per cell,” which appears to be a typographical error and should be corrected.

10) Line 607: The gene MS4A1 is mentioned repeatedly in Line 607, which may be redundant and should be revised for clarity.

11) References 25 and 26 are cited to support a claim that genes involved in “response to metal ion binding” in enterocytes are protective against intestinal inflammation. However, both studies cited appear to be based only on metallothionein (MT) and in non-intestinal tissues, making their relevance to this claim questionable.

12) In Line 168, the phrase “inter-individual differences” is used, yet the data presented do not seem to address variability across individuals. The terminology should be revised unless such variability is explicitly analyzed.

13) The section spanning Lines 193–203 presents findings without any accompanying figures or data, which weakens the support for the claims made.

Referee #2

(Remarks to the Author)
I co-reviewed this manuscript with one of the reviewers who provided the listed reports.

Referee #3

(Remarks to the Author)
Alegbe et al. assembled a single-cell atlas comprising 1.8 million cells from blood, terminal ileum, and rectal tissues from 296 healthy donors and 125 patients with Crohn's disease to enable psuedo-bulk cis-eQTL mapping at cell-type resolution. They identified eQTLs for 20,389 genes, and demonstrated that increasing cellular granularity reveals lead eQTL variants are located more proximal to the TSS and in functional enhancers. Through ‘interaction eQTL’ (ieQTL) mapping, they demonstrate genetic effects attenuated by gut inflammation status. In some example loci, they highlighted heterogeneous effect sizes across cell populations and speculated their functional role in IBD in a cell-state specific manner. Using colocalization analysis, they show cell-type level associations are more likely to colocalize with GWAS loci, nominating effector genes at more than half of all reported IBD loci by their criteria. Finally, they explore whether nominated genes and

the appropriate cellular context can inform therapeutic targets through potential drug repurposing.

I think that the data and summary statistics they generated would become valuable resources for the functional understanding of gene regulation in the gut, but more focused analysis and discussion might be warranted for suggesting novel hypotheses for causal gene discovery in the IBD genetics. Counting the overlapping and colocalizing loci in any of the cell types in the gut might not be sufficient to claim their causal role in pathogenesis.

Major comments:

1. The primary findings they mentioned (e.g., the lead variant is distant from TSS in cell-type-specific eQTL) have been continuously shown in the other studies even with bulk RNA-seq (such as, M. Gutierrez-Arcelus et al. PLoS Genetics 2015, Brown et al. PLoS Genetics 2013, GTEx 2020, etc.). Can the authors acknowledge and discuss these previous findings and further elaborate on the novel concepts they were able to identify through the usage of single-cell tissue data?
2. There also exist single-cell eQTL studies investigating immune cell types (Yazar et al. 2022, Perez et al. 2022 etc.). To what extent do the immune cells from this tissue-specific data lead to novel discovery of IBD GWAS genes that had not been discovered in such peripheral blood studies?
3. Relatedly, how many of target genes through the colocalization were not identified in previous studies in bulk RNA-seq or single-cell RNA-seq. Can the authors find any features that led to increased discovery using the tissue- and disease-related dataset?
4. A recent study in colon tissue from IBD patients revealed a subset of eQTL that were not found in non-IBD tissue, and larger effect sizes for eQTL in disease tissue (Nishiyama et al. bioRxiv 2024). For terminal ileum in which they both collected samples from healthy and IBD individuals, have the authors observed heterogenous effect sizes between disease vs. control that could be due to differences in cell state or cell type compositions? The authors should highlight the value of having both sets of samples.
5. Can the authors clarify the method used for ieQTL analysis and colocalization? There were two terms involving the interaction covariate: I and GT:I. The ieQTL discovery was on the coefficient for I, GT:I, or both? Also, how was the colocalization done with the ieQTL signal? If it is based on the significance of GT:I term, is that valid analysis to test shared causal variant probability between eQTL and GWAS?
6. While the authors sometimes provided biological interpretation of their findings based on previous literature, relying solely on evidence from any colocalization in any cell types to support a model in which an eQTL mediates disease risk may not be sufficient. For example, linkage disequilibrium can lead to spurious colocalizations. In addition, as we analyze more cell types, we expect to identify more different sets of causal variants, and some may be more likely to colocalize with GWAS, even if it appears to be random and not in a cell type with additional evidence supporting its role in disease. To ultimately prove the causality of the target gene and variant from this eQTL study, experimental support (e.g., gene KO or variant editing) for selected loci would be necessary.
7. The assumption that a single causal variant underlies the observed association with disease at GWAS loci may not always hold as previously shown. Integrating fine-mapping, such as with the SuSiE framework, can relax the single causal variant assumption by simultaneously estimating distinct signals and quantifying the evidence that each causal SNP is responsible for the observed colocalization. Such analysis could potentially increase colocalization signals with their higher resolution eQTLs (All Cells/Major Population) and provide stronger evidence for their observed colocalizations.
8. In the FUBP1 example, the authors mentioned the opposing directional effect on gene expression that is associated with CD risk loci. This type of analysis can be tricky, since variant(s) in LD, which are causal in one cell type, can affect the sign of the causal variant in the other cell type. Did the authors confirm that they were able to fine-map this locus in all the cell types, and they find the same variant as causal in all the cell types, but still they observe opposing effect directions?
9. In lines 183-185, the authors state, “To benchmark our findings, we compared them to colocalisations previously reported for IBD loci in the Open Targets Genetics portal, including those based on expression, splicing, and other molecular QTLs.” Use of Open Target to compare against their causal gene links can be tricky, since the Open Target includes multiple sources of information for V2G links, which include eQTL but also other sources like pQTL and in-silico predictions. When validating their disease genes, it would be more valuable if they compare their links separately against each individual source to more clearly interpret the additional value of this study.
10. The arguments about drug safety and repurposing seemed largely conjectural and lacking thorough evidence. For example, they mention ITGA4, JAK2, & IL23R eQTLs as direct molecular targets of current drugs, but don’t describe whether the eQTL effect aligns with the molecular mechanism like they do for their other examples. They loosely mention some other eQTLs that are in immune pathways modulated by IBD drugs saying the pathways are “active across multiple cell types and tissues.” In addition, the arguments on diarrhea which they thought as phenotypically related with IBD as drug side effects of metformin and γ-secretase inhibitors are too speculative, relying solely on a single-gene eQTL result while such effects must involve multiple genes and pathways.

Minor comments:

1. Figures
Figure 2A: It’s not readily clear how the varying resolutions produce 384 possible annotations and could be made more apparent.
Figure 3A: The number of loci from Open Target Genetics portal is inconsistent between the figure (n=137) and the text (n=138).
Figure 3E: This figure panel is not presented in order, and only introduced later after Figure 4. It may be more clear to restructure the results accordingly.
Figure 4B: I would recommend changing the colors here, because both the light grey and yellow are hard to read on the axes, but as mentioned earlier, it would be more informative to color by LD here, as well as Figure 5A-C.

Figure 6: The spacing along the x-axis needs to be increased, some of the text is overlapping and is illegible.
Figure S7: It would facilitate easier interpretation of the results if the authors provided a definition for "maximum specificity of expression" in the figure legend instead of just citing CELLEX.
Figure S13: I don't know that this figure is showing anything particularly informative. Both the axes and legend are too small to read, and even when zooming in the resolution is too low to read.
2. The authors should add to the methods section on ieQTL mapping whether biological sex as a variable is self-reported or genetically-inferred?
3. In lines 160-162, the authors state, "Although most ieGenes (941; 96.3%) were also eGenes, only 441 ieQTLs (4.2%) were in strong LD with eQTLs ($R2 > 0.5$), indicating these effects are largely distinct from those observed in conventional eQTL analyses." At a more relaxed LD threshold ($R2 > 0.1$) what proportion of ieQTL effects are distinct from eQTLs ? Are these analyses all on lead variants?
4. In lines 215-217, the authors state, "To refine effector cell-type hypotheses, we identified, for each colocalised signal, the cell type in which the lead variant explained the greatest proportion of variance in gene expression." Is this referring to the lead eQTL variant or the lead GWAS variant at that locus?

(Remarks on code availability)
A script used for the colocalization was inaccessible to the reviewer. The exact script for QTL mapping was not available, as the repository was for general QTL mapping but not specifically for this study.

Referee #4

(Remarks to the Author)
I co-reviewed this manuscript with one of the reviewers who provided the listed reports.

Referee #5

(Remarks to the Author)
There is significant interest in understanding the molecular mechanisms that underlie genetic associations with disease. While expression quantitative trait loci (eQTLs) derived from bulk RNA-seq have been widely used for this purpose, they have not consistently delivered on the promise of elucidating causal mechanisms. One proposed explanation for this shortcoming is that bulk RNA-seq may mask cell type-specific expression variability that is relevant to disease. In this impressive study, Alegbe, Harris et al. investigate variants linked to inflammatory bowel disease (IBD) and their overlap with cell type-specific eQTLs identified from a large collection of single-cell RNA-seq data derived from blood and intestinal biopsies. This work is both important and timely, particularly as the field considers how best to use functional genomics to interpret complex trait genetics. The authors carefully map eQTLs across diverse cell types and demonstrate significant overlap with IBD GWAS loci, an observation with important implications for explaining the often-limited overlap between eQTL and GWAS signals. Building on this set of effector gene candidates, the authors highlight the roles of Notch and Wnt signaling in IBD pathogenesis and nominate several potential drug targets. Overall, the study is rigorously executed and provides findings of broad relevance to both the IBD research community and the larger field of complex trait genetics. In addition to the scientific insights, the dataset represents a valuable single-cell resource that will be broadly useful. I have not identified any flaws that would preclude publication, and assuming appropriate responses to reviewer comments, I would likely recommend the manuscript for publication.

Major
- In the two sections of the results implicating Notch and Wnt signaling in IBD, the strength of evidence for prioritizing these pathways over others was unclear. In the Notch section, two of thirteen cDC effector genes (or loci?) are noted to be involved in Notch signaling, but is this more than expected by chance? Was any enrichment analysis performed? Similarly, the Wnt signaling section refers to implicated genetic variation, but it is unclear how the connection to Wnt was statistically established. I did not find a comparable statistical argument supporting Wnt's involvement in the main text. If the authors are making pathway-level claims, it is essential that these be supported with appropriate enrichment or statistical tests relative to a null model.

- The observation that over 75% of GWAS colocalizations overlap with those from Open Targets is encouraging, but further validation of identified eQTLs would strengthen confidence that these eQTLs are not dataset-specific artifacts. Are there existing datasets that can be used to assess the overlap of these cell type-specific eQTLs with prior observations? While this dataset is unique, it may still be possible to show concordance for a subset of eQTLs. For instance, bulk RNA-seq-based eQTLs from GTEx could serve as a point of comparison for the 'all cells' analysis that aggregates data across the terminal ileum tissue. The blood eQTLs could perhaps be validated with other large-scale blood single-cell resources (Yazar et al.). While I recognize that similarly powered resources may not exist for validating interaction eQTLs, additional benchmarking of standard eQTLs would help support the robustness of the findings.

- The manuscript would benefit from a stronger discussion of how these findings relate to other large-scale single-cell eQTL datasets that assess GWAS overlap. For example, Yazar et al. examined the intersection of immune traits with single-cell eQTLs in blood. Do the current findings corroborate or extend what has been described in such studies? In addition, are there prior reports that support a role for Notch or Wnt signaling in IBD genetics? Connecting the results to prior literature more directly would help readers interpret the novelty and robustness of these conclusions.

- The role and interpretation of interaction eQTLs could be clarified. These analyses are potentially very insightful, but were not explained in enough depth. For example, regarding the association with RNF14, the authors suggest that the risk allele reduces RNF14 expression in inflamed enterocytes. However, as I understand it, the measured ileal inflammation is not specific to enterocytes, so this interpretation may be inaccurate. Overall, the biological interpretation of interaction effects should be more precisely described.

- The main text should include more detail on the statistical thresholds used for eQTL discovery. For example, line 117 reports 84,376 eQTLs, but it is unclear what significance cutoff was applied. Providing this information is essential for evaluating the strength of the reported associations. I did not find clarification on this point in the Methods section either. What p-value threshold was used, and how was multiple hypothesis testing addressed?

Minor
- In several figure panels, the text is too small and the colors too soft, making them difficult to read. Notable examples include the figure legend text and gene names in Fig. 4 (especially panel b), Fig. 5, and the bottom column labels in Fig. 6. This issue appears in other figures as well and should be addressed for overall readability.
- Fig. 2e: The two values above the violin plot should be clearly labeled with units (e.g., "%" and "MB") to avoid confusion.
- Fig. 3a: Although the main text provides context, the numbers outside the pie chart are unclear. Please clarify their meaning in the figure legend or annotate the panel directly.
- Fig. 3b: The figure legend does not clearly explain what the odds ratio on the y-axis represents. A more detailed description would improve interpretability.
- Line 600 (Methods): The phrase "quality control and QC" is redundant. Please remove either "quality control" or "QC."
- The link for the colocalization code was not working: https://github.com/andersonlab/snakemake_colocalisation

(Remarks on code availability)
I did not run the code but I skimmed through it. Most of the code was available. There was one repository that wasn't available: https://github.com/andersonlab/snakemake_colocalisation

Version 1:

Reviewer comments:

Referee #1

(Remarks to the Author)
The authors have done an outstanding job addressing the concerns of the reviewers and have clarified most of the concerns the reviewers had. Appreciated the length and depth they went to explain things in their rebuttal letter. Still think the study leaves open many unanswered mechanistic questions that need experimental validation, but the authors have argued this is beyond the scope of the study. Major concern with this is that there are already over 200 loci that have been identified for IBD, and except for a few, we still don't know the mechanisms or how they might contribute to disease- thus we have been left with many associations and suggestions on their impact. Nonetheless, the bioanalytical methodology they presented is rigorous and well done, and agree that resolving the eQTLs at higher resolution is a major step forward in functional genomics.

Referee #2

(Remarks to the Author)
I co-reviewed this manuscript with one of the reviewers who provided the listed reports.

Referee #3

(Remarks to the Author)
We appreciate the authors' response to our comments and additional analyses which clarified the novelty of this study. We are satisfied with most of the points that were raised in the first review round, but have several remaining points that we think need to be addressed for the publication of the manuscript, which we have summarized below.

Major:
(Previous comment #2): It was great to see that the authors were able to nominate effector genes through colocalization with their eQTL that had not been identified by Cuomo et al. However, we thought that it would be also important to mention what percentage of colocalizations by Cuomo et al. were not identified in the authors' study using colonic tissues, for a fair comparison. For the genes that were only identified by their study, it would be of interest to assess what contributed to the differences between this work and Cuomo et al. For example, whether eQTL from the cell types specific to the colonic immune cells / colonic tissues were more likely to have contributed to the additional discovery, since this study is statistically less powered than Cuomo et al.?
(Previous comment #5): Thanks for the clarification on the methods. We appreciate that the authors conducted a simulation

study of the power to detect colocalization. However, we are still unsure if the colocalization using the interaction term is valid or not. GT:I term can become significant not only in the cases when the true causal variant of GWAS causes gene expression changes in a subset of cells but also in the cases when the variant has significantly heterogenous effect sizes without any apparent subset of cells which drive the association of both expression and GWAS. The latter may not serve as a causal mechanism of GWAS, but rather be a coincidence of a variant with heterogenous effect sizes with expression and a GWAS variant. In the real examples of colocalization between GT:I and GWAS, can the authors show that there exist cell states that demonstrate a causal variant with a significant main effect (G) for the gene expression and also with high PIP with GWAS?
(Previous comment #8): The authors explain in their rebuttal that fine-mapping of their eQTLs would not be appropriate given the vast differences in statistical power across cell types represented in their dataset, and instead attempted to address this comment with an LD clumping strategy. While LD clumping is useful for approximating independent signals, it does not facilitate identification of the likely causal variant and importantly, it cannot correct for LD-induced sign flips. We would also like to argue that by performing colocalization analyses in other sections, the authors have implicitly already performed fine-mapping analysis. If myofibroblasts do not share exactly the same causal variant as rectal cDC2 and T cells, but instead have causal variants that are in LD, the eQTL effect truly represents a mixture of these signals, which is particularly important when the effect is marginal, as is the case for the “chr1:77461459:A:G” clump index the authors demonstrate. Therefore, we believe that fine-mapping even with caveats is necessary to support the claim of opposite directional effects between cell types, which would represent a very interesting finding of this study worth highlighting.
(Previous comment #9): We appreciate the authors’ rebuttal that the purpose of their study was building a resource for hypothesis generation. Given that big data resources are being published so frequently, it is important for the authors to make a clear case for the utility of theirs with respect to what is currently available. In regard to the benchmarking of this study using data from the Open Targets Genetic Portal, there is still more that can be done to appropriately place this resource in the broader context of IBD genetics research. In lines 200-204 the authors state “Among the 321 IBD loci reported to date, we observed colocalisation with either eQTLs or ieQTLs at 180 loci, implicating 412 distinct effector genes. This includes 74 loci where no effector gene had been previously nominated by colocalisation in the Open Target Genetics portal and 257 novel disease effector genes.” From the text it is unclear whether the 257 novel disease effector genes are only at the 74 loci, or if they include novel effector genes at previously resolved loci? Later, on lines 213-214 the authors state “Our eQTLs and ieQTLs also colocalised with 104 of the 137 GWAS loci already reported to colocalise with molecular QTLs in the Open Target Genetics portal. At 78 of these loci, at least one effector gene colocalised in both our data and the Open Targets analysis.” Can the authors provide an explanation for the 33 loci which they do not replicate in this study? Were those previously reported colocalisations not with eQTL and rather other molecular QTLs? Can the authors provide an explanation for the 26 loci where they nominate different effector genes than previously reported? Were those previous reports from eQTL studies, or other molecular QTLs, and in what tissues?

Minor:
There are both main and supplementary figures (e.g. Figure 4A-B or Figure 5A-C) where some panels have boxes surrounding the figures, and these should be removed as it is not stylistically consistent.
In Figure 3A the text overlapping the pie chart should be moved to the side with lines directing it to the relevant fraction because currently some of the letters are not visible.
In Figure 3B the “0.75x” text should be above the “All Cells” point on the plot similar to the text above the points for “Major population” and “Cell type”.
In Figure 4B the “Fraction of cells in group (%)” text is overlapping and it’s not necessary to label each break point. This is also true in Supplementary Figure 15.
The legend for Figure 5 needs to include mention of “lead variant presented as diamond” which they have in Figure 4 legend, but it should be in both.
The colors chosen for “novel” and “in OTG” are so close to each other that I’d recommend the authors choose something more distinct. They could remove the y-axis label “mechanism of action” from Figure 6 because it kind of gets lost anyway, and just mention it in the figure legend.

(Remarks on code availability)
For the core scripts for eQTL mapping https://github.com/andersonlab/IBDVerse-sc-eQTL-code, the README file is missing and I was not able to find scripts to pseudo-bulk single-cell data and run eQTL analyses. The authors should make these scripts clearly available.

Referee #4

(Remarks to the Author)
I co-reviewed this manuscript with one of the reviewers who provided the listed reports.

Referee #5

(Remarks to the Author)
The authors have addressed my previous concerns.

(Remarks on code availability)
I briefly looked through the provided github and while I didn't run the code it appeared to have sufficient detail to do so.

Version 2:

Reviewer comments:

Referee #3

(Remarks to the Author)
I sincerely appreciate authors' thorough analyses and response to my previous comments. The manuscript is now much more compelling, along with clear explanations on the limitations and with many open questions to the field. I am happy to see more discussions on similarities and dissimilarities with previous studies and across cell types. In addition to releasing this dataset and summary statistics, this paper would become a very useful resource to the field to investigate these open questions on eQTL and gene regulation in general.

(Remarks on code availability)
https://github.com/andersonlab/pi1_pairs is not yet accessible to public.

Referee #4

(Remarks to the Author)
I co-reviewed this manuscript with one of the reviewers who provided the listed reports.

Open Access This Peer Review File is licensed under a Creative Commons Attribution 4.0 International License, which permits use, sharing, adaptation, distribution and reproduction in any medium or format, as long as you give appropriate credit to the original author(s) and the source, provide a link to the Creative Commons license, and indicate if changes were made.
In cases where reviewers are anonymous, credit should be given to 'Anonymous Referee' and the source.
The images or other third party material in this Peer Review File are included in the article's Creative Commons license, unless indicated otherwise in a credit line to the material. If material is not included in the article's Creative Commons license and your intended use is not permitted by statutory regulation or exceeds the permitted use, you will need to obtain permission directly from the copyright holder.
To view a copy of this license, visit https://creativecommons.org/licenses/by/4.0/

Below, we have included our point-by-point response to reviewer comments (in **bold**), with our responses below. Where relevant, we have included reference to lines in the revised manuscript as (LX), where 'X' indicates line number, and our responses are formatted in italics. We have also numbered the comments so that they can be easily referenced across our responses, and provided a list of sources cited in this response at the end of the document.

Referee #1 (Remarks to the Author):

This manuscript tests the idea that bulk sequencing analysis can miss certain traits whose effects are at the level of the cell type. Their starting framework was that genome wide analysis (GWAS) has revealed that single nucleotide polymorphisms (SNPs) associated with IBD risk are more likely to reside within gene enhancers while regulatory effects like eQTLs shared across multiple cell types are more likely to reside in promoters. The authors use single cell RNA sequencing data combined with genotyping from each patient to determine whether there are additional eQTLs at the cell type level and what the location was on the chromosome. They built the IBDverse, a collection of about 2 million cells from about 400 patients (about one-third having IBD), including blood cells along with cells isolated from ileal and rectal mucosal biopsies and showed that additional genes exist at the cell type level that have eQTLs farther away from the promoter than those previously detected. About 150 of these eQTLs mapped to the same region as known IBD risk genes (about half of the 300 IBD risk loci). Additional analysis revealed that inflammation during IBD affected how these eQTLs impacted the effector gene (eGenes) expression. The conclusion being that many IBD risk loci at enhancers are eQTLs whose effects can only be detected by single cell analysis.

The approach is valid, the work is significant, and the quality of the data is high. Although small effect genes / products can have major influence on biology, my first major point would be that it is still unclear the impact some of these small effects at the cellular level would have on the biology of the disease, and how much weight these observations carry overall in biological significance. Especially when considering that mRNA levels are not always good indicators of protein levels and activity. A major technical point that directly ties to the first concern is that despite the high number of cells used in the study, the statistical power of single cell data is so limited by the few transcript copies per gene that make their way into the library for each cell that this sparsity (some genes might only have 10 or 5 or even 1 transcript copy per cell) makes it difficult to appreciate how truly robust these analysis are, especially considering it still seems questionable which chromosome with the SNP served as the template for those transcripts and so on. It seems the study relied on statistical power from the number of cells, which allowed for the transcripts of that subpopulation of cell types to be analyzed in a pseudobulk manner, in my mind calling into question the single cell aspect of the study. Thus, it is also still a question of biological significance or technical that eQTLs are enriched in single cell analysis. I think more elaboration on how the statistical framework dealt with these biological and technical factors is needed to help bolster the claims, along with some type of validation and experimental study to show how these eQTL influence or produce a phenotype. The work is somewhat novel, but lacks experimental validation, and relies

too heavily on one set of methodology, and the text is difficult to read in the parts of the manuscript that tied the eQTLs to molecular genetic behavior. The study leans heavily on existing literature to establish links between candidate genes and IBD without offering new biological insights.

We sincerely thank the reviewer for their careful consideration of our manuscript. In this opening summary, there are a few points we would like to address.

a. "still unclear the impact some of these small effects at the cellular level would have on the biology of the disease, and how much weight these observations carry overall in biological significance".

It is now well established that drug targets with genetic associations supporting the target-indication pair are more likely to succeed through clinical trials (Nelson et al. 2015; Minikel et al. 2024; Trajanoska et al. 2023; Razuvayevskaya et al. 2024). This genetic support typically comes from genome-wide association studies that most often associate common variants of small effect with disease. Robust biological hypotheses can be built by identifying the effector gene(s), cell types(s) and pathway(s) that are dysregulated by a given disease associated variant. While these individual genetic effects are small, co-inheritance of independent risk alleles at multiple risk loci, especially those that converge on the same biological pathways, can confer larger, additive effects on disease. It is for this reason we focus on disease effector genes that are associated with broad biological functions (e.g Notch and Wnt signalling) and in the same likely effector cell-types (e.g dendritic cells) when highlighting examples of our novel findings. We also note that while modest perturbations on gene-expression often have only a small effect on disease status, the therapeutic hypotheses that this knowledge generates can be used to develop drugs that have a much stronger effect on the target and therefore a major effect on disease status.

b. "despite the high number of cells used in the study, the statistical power of single cell data is so limited by the few transcript copies per gene that make their way into the library for each cell that this sparsity (some genes might only have 10 or 5 or even 1 transcript copy per cell) makes it difficult to appreciate how truly robust these analysis are, especially considering it still seems questionable which chromosome with the SNP served as the template for those transcripts and so on. It seems the study relied on statistical power from the number of cells, which allowed for the transcripts of that subpopulation of cell types to be analyzed in a pseudobulk manner, in my mind calling into question the single cell aspect of the study"

There are a few points in this series of comments that touch on the cutting edge use of single cell RNA sequencing for eQTL mapping, and we are grateful for the opportunity to justify our approach here.

Firstly, we would like to outline some of the limitations pertaining to the use of single cell-level expression data for eQTL mapping:

1. **Sparsity due to technical dropout:** As the reviewer suggests, expression counts at a single cell level are inherently sparse. This can make the estimate of expression level unreliable, and so aggregation of cells improves the robustness and reliability of

expression counts.

2. **Statistical and computational burden:** Because multiple cells are collected from the same sample, these are not statistically independent from one another. Proper modelling of this therefore requires random effect terms in mixed models to avoid pseudoreplication bias. Such approaches drastically increase the computational burden of individual tests and given the number of conditions, samples, and cells we have collected, this makes genome-wide eQTL mapping in our dataset computationally infeasible.

3. **Performance:** Use of expression from single cells for eQTL mapping has been shown to not outperform pseudobulk approaches (Murphy and Skene 2022; Pullin and Wallace 2025).

This has made pseudobulking a standard, and popular practice for eQTL mapping from scRNAseq data (Soskic et al. 2022; Yazar et al. 2022; Nathan et al. 2022; Oelen et al. 2022; Bryois et al. 2022; Tian et al. 2024; Natri et al. 2024). The method we employ, calculation of the mean log-normalised expression, has been shown to outperform other pseudobulking methods in benchmarking studies (Cuomo et al. 2021).

In line with this, and in response to the specific comment - "**how truly robust these analysis are**" - we apply stringent quality control measures to limit the chance for spurious associations in our analysis. This includes; 1) removal of individuals with fewer than 5 cells for a given cell type, 2) not testing cell types with fewer than 30 contributing samples, 3) removal of genes expressed in less than 20% of samples for a given cell type (L974). Additionally, in response to comment 2 by reviewer #4/5, we now provide a Supplementary Note in the revised manuscript that indicates our eQTLs are well supported by GTEx and sc-eQTLs from Yazar *et al.,* (2022), but that this follows the biological similarity of comparisons being made.

With respect to the specific comment - "**it still seems questionable which chromosome with the SNP served as the template for those transcripts**" - Whilst we acknowledge the benefit of allele-specific expression analyses, it is not common practice for eQTL mapping studies to phase data and directly link genetic variants to the template transcripts. This is because eQTL summary statistics, without phasing information, are sufficient to determine effector genes via approaches such as colocalisation and mendelian randomisation (Yazar et al. 2022; GTEx Consortium 2020; eQTLGen Consortium et al. 12/2019). Nominating effector genes is the ultimate goal of our work, and so we do not feel incorporation of this information would help us in achieving this aim. Upon publication we will be releasing our raw data, and so this analysis will be possible for others to undertake.

With respect to the specific comment - **"calling into question the single cell aspect of the study"** - despite pseudobulking, we do believe that it is still appropriate to refer to our study as "single-cell", and that our choice of approach provides substantial advantages over alternatives. Our reasoning for this is as follows:

We hypothesised that GWAS signals would be enriched amongst contextually restricted eQTLs (L74), and so to test this we required collection of biologically distinct cell subsets at scale. One way this could be generated is by fluorescence-activated cell sorting (FACS) of

primary tissue based on surface marker proteins. However, this is very labour intensive, requires knowledge of the specific marker proteins *a priori,* and because sorting is limited by the number of detectable fluorophores, cell separation is based on only a handful of features. In contrast, single-cell RNA sequencing captures gene expression from an initially unknown number of cell-types, allowing the definition of such based on evidence from the expression of thousands of genes. Because there is currently no scRNAseq dataset that incorporates the blood, terminal ileum and rectum, especially not at a scale comparable with our dataset, we chose to derive our own cell-type annotations, identifying 86 transcriptionally distinct clusters. Our use of scRNAseq for this purpose therefore makes us uniquely placed to derive insights that existing bulk or FACS-based approaches could not, hence justifying our use of 'single cell' in this context. This is also in line with nomenclature used by other eQTL studies that pseudobulk expression from scRNAseq data (Soskic et al. 2022; Yazar et al. 2022; Nathan et al. 2022; Oelen et al. 2022; Bryois et al. 2022; Tian et al. 2024; Natri et al. 2024).

c. "The work is somewhat novel, but lacks experimental validation, and relies too heavily on one set of methodology, and the text is difficult to read in the parts of the manuscript that tied the eQTLs to molecular genetic behavior. The study leans heavily on existing literature to establish links between candidate genes and IBD without offering new biological insights."

While we acknowledge that our study does not include experimental validation and is based on a single methodological framework (single-cell eQTL mapping), we believe our work delivers multiple new insights and resources that significantly advance the understanding of complex disease genetics, both in IBD and more broadly.

Our goal was not to elucidate molecular mechanisms at a single locus, but to build a high-resolution map of regulatory genetic effects across hundreds of cell types and conditions relevant to IBD. This map provides a foundation for future experimental studies by enabling the systematic nomination of effector genes, cell types, and regulatory contexts at GWAS loci. In doing so, we facilitate rather than replace traditional reductionist approaches.

A key example of the long-term value of such colocalisation maps is the recent study by Stankey et al. (Stankey et al. 2024), which began with a colocalisation between an *ETS2* eQTL in macrophages and an IBD GWAS locus. This discovery led to a multi-year programme of experimental work revealing the causal role of *ETS2* dysregulation in disease pathogenesis. We see our study as enabling such efforts at scale: by systematically identifying and sharing these candidate gene–cell–context relationships, we provide the community with a prioritised set of hypotheses to test — spanning hundreds of loci. Our contribution is to make these hypotheses accessible now, so that diverse research teams with relevant disease and mechanistic expertise can explore them in parallel.

To this end, our work makes the following novel contributions:

A deep and disease-enriched scRNA-seq dataset.

We present the largest single-cell eQTL resource constructed to date for any solid tissue, comprising 2.2 million high-quality transcriptomes from 421 individuals (125 with Crohn's disease) and 732 samples from terminal ileum, rectum, and blood. Sampling includes healthy

and inflamed tissues, enabling regulatory comparisons across physiological contexts — a design feature rarely achieved in existing eQTL maps.

A comprehensive single-cell eQTL map.

We identify over 88,000 cis-eQTLs and 760 inflammation-dependent interaction eQTLs across 251 cell-type and tissue annotations. Notably, nearly one-third (29.3%) of the cis-eQTLs were cell-type-restricted and undetectable in coarser annotations, highlighting the added value of cell-type resolution. These eQTL associations can be colocalised with a catalogue of existing and future GWAS summary data to enable nomination of effector genes across diverse contexts.

Novel findings on the properties of cell-type-specific regulation.

We find that eQTLs detected at the cell-type level are more distal to transcription start sites, enriched in enhancer elements, and less likely to regulate the nearest gene than those discovered at tissue-level resolution. These features suggest a distinct regulatory architecture that underpins cell identity and state. Furthermore, these cell-type-specific eQTLs are over 3.5-fold more likely to colocalise with IBD GWAS loci than those identified at lower resolution. These results are of broad interest — to those studying the genomic architecture of gene regulation, enhancer biology, and mechanisms of disease association alike — and demonstrate why single-cell resolution is essential for interpreting non-coding genetic variation.

Biological relevance and translational potential.

By integrating our eQTL maps with IBD GWAS, we identify effector genes at over half of known IBD risk loci, including at 74 loci for which no effector had previously been statistically nominated. This includes new regulatory evidence for therapeutic targets (*ITGA4*, *JAK2*, *FERMT1*) and druggable genes implicated in other diseases (*PSEN2*, *NDUFAF1*), offering insights into possible pleiotropic effects and repurposing opportunities.

A scalable framework for future studies.

We provide a blueprint for using scRNA-seq and genotype data to systematically identify disease-relevant regulatory effects. The falling cost of single-cell technologies now makes it feasible for other groups to apply similar frameworks across different tissues and diseases — making our approach broadly applicable across human genetics.

While we do not include experimental validation, we draw on prior literature to contextualise our findings and highlight biological plausibility. But we are clear these mechanistic hypotheses remain to be tested. In response to reviewer comments, we have added the sentence below to the discussion to clarify this distinction and explicitly acknowledge the need for downstream experimental work.

L708: "*While experimental perturbation will be required to establish causality for individual genes and pathways, our results provide a focused set of hypotheses for effector genes, cellular contexts, and molecular processes underlying IBD.”*

In summary, we believe our study represents a substantial advance in the interpretation of GWAS signals and the construction of regulatory maps in disease-relevant tissues. It generates hypotheses at scale, contextualises existing associations, and provides an openly available resource that will support experimental research in IBD and other complex traits for years to come.

Comments/Questions:

1) Several papers published have used genetic and single cell level analysis, but not in IBD. The following are references of previous work in the field; PMID: 35389779, 40112817, 35328055. Another lab group appears to have also used similar technology using bulk RNAseq and analysis on an IBD cohort with similar sample size and results PMID: 33602935. This current manuscript used a large number of single cell, providing more than just incremental advances.

We thank the reviewer for their careful consideration of our work in the context of current literature, and appreciate the positive interpretation of the impact of our study.

2) The presentation was a bit challenging, and I had a difficult time reading through some of the text in the Results section and I didn't fully understand what was implied in certain instances. Given the descriptive nature of the study, I also thought there were several assertions made about the results regarding the impact on molecular genetic phenomena that were not fully supported by the data or clearly conveyed in the text. For example, it is stated: Line 168 "These findings demonstrate that genetic variation contributes to inter-individual differences in cellular responses to inflammation, and that interaction eQTL mapping using scRNA-seq data can identify context-dependent regulatory mechanisms relevant to disease."

We thank the reviewer for their evaluation of the presentation of our findings. To improve the clarity and readability of the manuscript, we sought feedback from colleagues across multiple relevant disciplines, including genetics, immunology, genomics, and gastroenterology. Based on their input, we have made edits throughout the manuscript to enhance clarity and accessibility, and we hope the reviewer now finds the paper easier to follow. If there are particular sections that remain unclear, we would greatly appreciate further feedback so we can address them more directly.

With regard to the specific sentence on line 168 of the original submission, our intention was to convey the following:

Some individuals' cells respond differently to inflammation, and part of this inter-individual variability is driven by genetic differences. By analysing single-cell gene expression data from biopsies with and without inflammation, we identified genetic variants whose effects on gene expression are modified by inflammatory status. These interaction eQTLs represent regulatory effects that are context-specific—emerging only, or changing, in the presence of inflammation. Notably, some of these inflammation-dependent eQTLs colocalise with IBD GWAS loci, suggesting they contribute to disease risk. Together, these findings support the value of

mapping genetic regulation under different disease-relevant conditions to uncover context-dependent mechanisms that would be missed in steady-state or healthy-tissue analyses.

In response to this comment, we have updated the sentence which now reads (L261):

"*Together, these findings support the value of mapping genetic regulation under different disease-relevant conditions to uncover context-dependent mechanisms that would be missed in steady-state or healthy-tissue analyses*"

3) More work needs to be done to merge the molecular genetic concepts with the more abstract and statistically based associations of eQTL and GWAS signals have on gene expression in the main text. For example, at Line 148 "Because GWAS loci are also enriched in enhancers rather than promoters, these findings suggest that eQTLs detected at higher transcriptional resolution may more closely align with the regulatory architecture of complex trait associations."

Again, we hope the changes we have made in response to feedback from a diverse set of internal reviewers has helped clarify our work associating molecular genetic features with our eQTLs and colocalisations.

For the given example, this statement refers to the relative enrichment of eQTLs detected at each cellular resolution ('All Cells', Major Population or Cell type level) in enhancers and promoters from FANTOM and ENCODE. Previous work (Mostafavi et al. 2023), which we cite in the introduction (L62), has shown that GWAS signals are enriched in enhancers rather than promoters, but that eQTLs are enriched in promoters as opposed to enhancers. We have used the same analytical approach as Mostafavi et al, but compared the enhancer vs promoter enrichment across different classes of eQTLs detected in our study. In keeping with the findings of Mostafavi et al, we show that eQTLs detected at the 'All Cells' level are enriched in promoters as opposed to enhancers. However, we also show that eQTLs detected solely at the cell-type resolution are enriched in enhancers rather than promotors, in keeping with the enhancer enrichment Mostafavi observed for GWAS loci. This supports our finding that eQTLs found at the cell-type resolution are 3.5 fold more likely to colocalise with an IBD GWAS hit than those found at the "All Cells" level. To improve clarity of this statement, we have updated this sentence to now read (L211):

"*Because GWAS signals also tend to be found in enhancers (Mostafavi et al. 2023), these findings suggest that gene expression changes driven by eQTL signals found only at a cell type resolution are more likely to mediate phenotypic differences in complex traits and diseases*"

4) What about chromatin structure or accessibility at these locations the authors identified? Or transcription factor site binding? There were also several words used like "colocalized" that had an unclear connotation. Sometimes it seemed to be used in a physical sense of chromosomal location and other times in a more abstract, association-like context when tied to a phenotype.

We thank the reviewer for highlighting the role of chromatin structure, accessibility, and transcription factor binding. Our primary aim was to nominate disease effector genes at scale

by identifying those dysregulated by IBD-associated variants in diverse cellular contexts. While assays of chromatin structure (e.g. Hi-C) or accessibility (e.g. ATAC-seq) have been valuable for interpreting regulatory mechanisms, they do not by themselves resolve target genes or capture disease-relevant effects in primary tissue at single-cell resolution. For this reason, we focused on sc-eQTL mapping, which more directly links genetic variation to gene expression changes in relevant cell types.

We agree that many sc-eQTL variants may act by altering transcription factor binding, and integration with TF binding data could provide mechanistic insights into upstream regulation. However, such analyses would complement, rather than alter, our primary goal of effector gene nomination, and are beyond the scope of the present study.

Finally, we recognise the ambiguity in our use of "colocalised." We now explicitly define colocalisation on first use as (L277):

"*This method tests the likelihood that two traits, in this case eQTL and GWAS associations, share a causal variant at a given genetic locus (Giambartolomei et al. 2014)*"

5) Use of the word effector cell types lines 242 is not clear how they can be labeled as such based on association data, then also mentions effector genes in line 245....so the word effector is a bit vague

We appreciate the reviewer's point about the ambiguity of the term "effector." In our study, we use "effector cell type" and "effector gene" in a specific, operational sense: the cell type or gene in which the colocalised eQTL–GWAS signal provides strongest support for mediating disease risk. Because germline genetic variants are fixed at conception and are not altered by gene expression changes, associations between genotype and expression are generally interpreted as reflecting genetic regulation of gene expression rather than the reverse. On this basis, we use "effector" to denote the most likely target gene or cell type of the regulatory variant at a given locus. This usage is consistent with recent disease-focused colocalisation studies, e.g. (Tian et al. 2024; Law et al. 2024). To make this clear, we have revised the text at L346 to read:

"*To refine hypotheses for the cell types in which colocalised effects may be active, we identified, for each colocalised signal, the cell type in which the lead eQTL variant explained the greatest proportion of variance in gene expression (Methods), and refer to these as 'effector cell types'*"

6) How can this statement about expression regulation be made based on association data? We identified 84,376 eQTLs regulating the expression of 20,389 of the 24,761 genes tested 118 (82.2%)

As outlined in our response to Comment 5, germline variants are fixed at conception and not influenced by downstream molecular traits, so associations between genotype and expression are generally interpreted as consistent with genetic regulation. Using this framework, we tested 24,761 expressed genes for cis-associations and identified eQTLs for 20,389 (82.2%). After testing for independent signals per gene across conditions, this yielded 88,548 distinct

eQTLs. We have clarified this wording in the manuscript to emphasise that these numbers reflect associations consistent with genetic regulation of expression (L168).

"*We identified 88,548 eQTLs associated with the expression levels of 20,389 of the 24,761 genes tested (82.2%)* (*genome-wide false discovery rate [FDR]<0.05, Methods*)*. Because the germline genome sequence remains fixed from conception, this represents genetic variation that confers a regulatory effect on the level of gene expression.*"

7) The description of the patient recruitment numbers is not clear, nor is which biopsies, blood, etc came from which patients. The Introduction mentions UC, part of the Results mention UC, but there is no mention of UC recruitment or sample collection. Were the rectal biopsies collected from different patients than the ileal biopsies? If so, is this accounted for in the analysis? The descriptions of the IBD phenotypes/metadata are not sufficiently detailed.

We thank the reviewer for highlighting the need for clearer description of patient recruitment, sample provenance, and UC relevance. We have revised the manuscript and added metadata to address these points:

1. **Recruitment and metadata**. Details of patient recruitment are provided at L745-752 and in our previous study (Krzak et al. 2024). To further aid clarity we will be providing the full metadata (containing age (binned), sex, disease status, inflammation, tissue, and smoking status) for each sample alongside our data release on EGA upon publication. We now refer to this in the revised methods (L752):

   "Sample-level metadata is available through EGA"

2. **UC patients**. We did not recruit UC patients. However, we obtained rectal biopsies from healthy individuals, the anatomical site most frequently involved in UC. We colocalised eQTLs across all sites and annotations with both UC and CD GWAS, and report multiple UC associations (e.g. Table 1). We also acknowledge in the Discussion (L620) that direct sampling of UC patients would be valuable.

3. **Sample provenance**. Rectal and ileal biopsies were collected from the same healthy individuals, and blood and ileal biopsies from the same CD patients where possible. These details are now clarified in the Methods and provided in data released on EGA (L750).

   "*Where possible, rectal and ileal samples were collected from the same healthy individuals, and blood and ileal samples were collected from the same CD patients.*"

4. **Cross-site analysis**. Within-site analyses use a single sample per patient. For cross-site analyses, we collapsed samples into one pseudo-sample per individual, so each person contributed only once. We therefore did not explicitly model correlation between samples from the same individual.

8) Why was there twice the number of cells encapsulated for scRNA-seq for IBD patients than for controls? How was the difference in sequencing depth across these samples accounted for? Additionally, given these differing targets, it would be important to report on the number of cells and the median number of genes per cell before and after quality control across both IBD and non-IBD samples

We thank the reviewer for their thorough examination of our study design. We targeted a higher number of cells from CD biopsies because IBD gut tissue exhibits marked immune infiltration and increased cellular heterogeneity. Capturing more cells improves representation of diverse cell populations and preserves power to quantify expression variation per annotation and map eQTLs. Sequencing depth was controlled by targeting 50,000 reads per cell for all samples; downstream analyses use standard per-sample normalisation (see Methods L764).

To directly address the reviewer's concern, the figure below compares terminal ileum samples from healthy and CD individuals. It confirms that more cells were captured from CD samples ($p<2.2e-16$) but that sequencing depth of each sample, as measured by median counts within each cell, did not differ across healthy and CD samples ($p=0.27$). This is now included in the revised manuscript as Supplementary Figure S13.

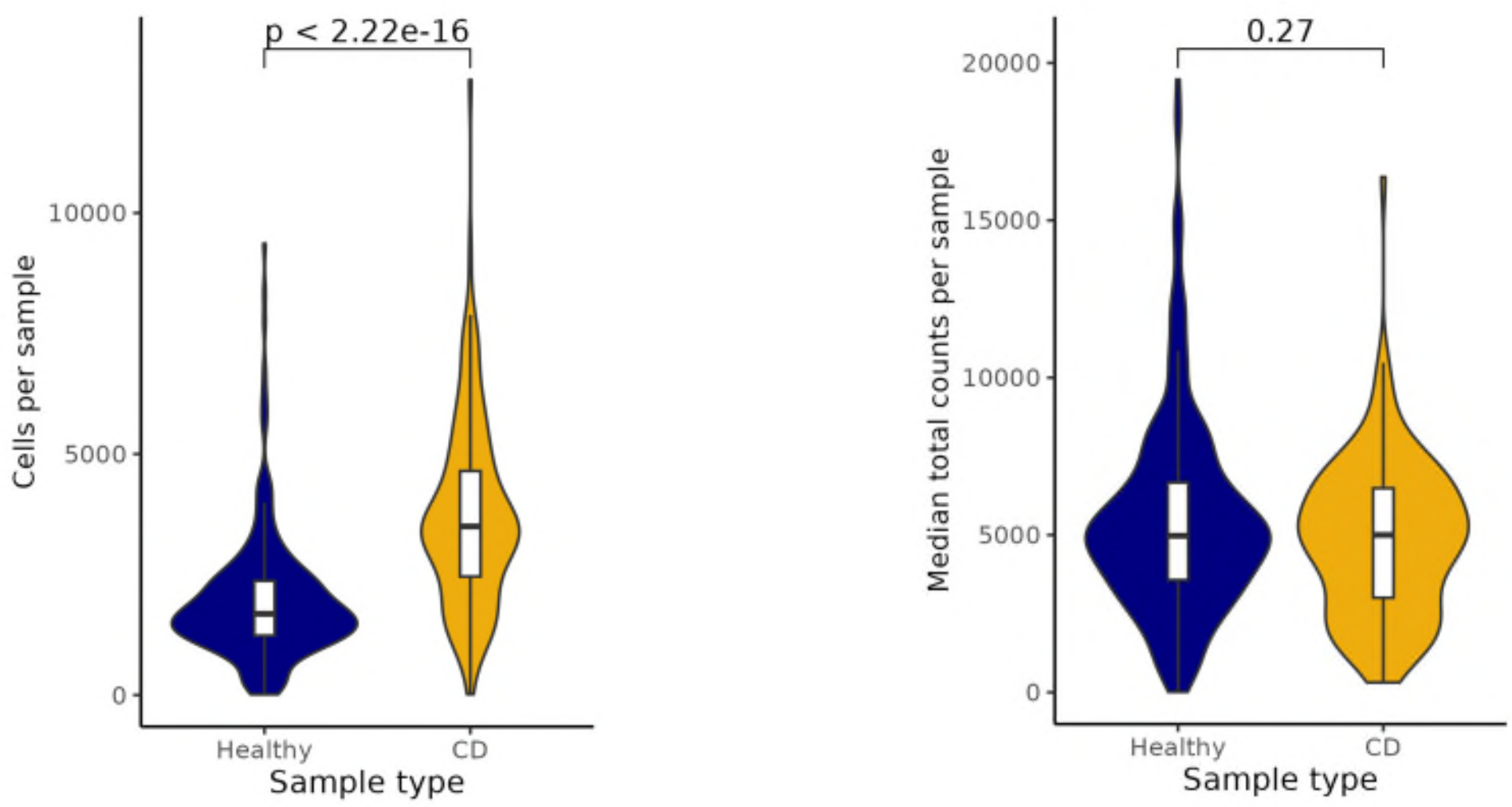

For clarity, we have also added the following text to the revised manuscript:

L759: "*Because IBD tissue displays increased cellular heterogeneity due to immune infiltration, we targeted 6000 and 3000 cells for CD and healthy biopsies respectively. This is to ensure adequate representation of diverse cell populations and to maintain statistical power for expression quantification and eQTL mapping across annotations.*"

L767: "*The greater number of cells targeted for CD patients is confirmed in the TI samples from the 'atlasing' dataset (see 'Single-cell RNA sequencing quality control and cell clustering') (wilcoxon p-value<2.2e-16), with no discernable influence on sequencing depth, as indicated by median number of counts per sample (p=0.27) (Supplementary Figure S13).*"

9) Line 525 reports “50,0000 reads per cell,” which appears to be a typographical error and should be corrected.

We thank the reviewer for spotting this typographical error. The text has been corrected to read “50,000 reads per cell” (L764).

10) Line 607: The gene MS4A1 is mentioned repeatedly in Line 607, which may be redundant and should be revised for clarity.

Again, we thank the reviewer for noticing this error. This repetition has now been corrected.

11) References 25 and 26 are cited to support a claim that genes involved in “response to metal ion binding” in enterocytes are protective against intestinal inflammation. However, both studies cited appear to be based only on metallothionein (MT) and in non-intestinal tissues, making their relevance to this claim questionable.

We thank the reviewer for their careful examination of our cited sources, and apologise for any lack of clarity in the relevance of these claims. There are four groups of metallothionein gene families in humans (named 1-4), which consist of eleven different isoforms: *MT-1* (*A, B, E, F, G, H, M*, and *X*), MT-2 (known as *MT2A*), *MT-3*, and *MT-4* *(Laukens et al. 2009)*. Each of these encode low molecular weight proteins which are capable of binding metal ions (Atrian and Capdevila 2013). Although we believe Inoue *et al.,* (previous reference 25) and Takano *et al.,* (previous reference 26) indeed provide supportive evidence for the role of these genes in inflammation, they both relied on genetic knock out of multiple *MT-1* and *MT-2* genes simultaneously, hence their collective reference as ‘metallothionein’, and the present lack of clarity.

To improve this, we have updated our text to mention specific leading edge genes, provide additional sources, and specify the specific tissues from which their evidence is derived (L256):

“*Notably, the most enriched pathway was ‘response to metal ion binding’ in enterocytes (coefficient = 2.04). Leading edge genes encode numerous isoforms of metallothionein proteins that bind metal ions (Atrian and Capdevila 2013), such as MT2A, MT1H and MT3 - genes also associated with protective effects against inflammation across several tissues including the skin(Sirvent et al. 2023), blood(Giacconi et al. 2005), liver (Zhang et al. 2021), lung(Takano et al. 2004) and intestine(Tsuji et al. 2013; Inoue et al. 2006).”*

12) In Line 168, the phrase “inter-individual differences” is used, yet the data presented do not seem to address variability across individuals. The terminology should be revised unless such variability is explicitly analyzed.

We thank the reviewer for their additional comment regarding this specific statement, and highlight our response to comment 2 by this reviewer. This statement has been updated for clarity and now reads (L261):

“Together, these findings support the value of mapping genetic regulation under different

disease-relevant conditions to uncover context-dependent mechanisms that would be missed in steady-state or healthy-tissue analyses"

13) The section spanning Lines 193–203 presents findings without any accompanying figures or data, which weakens the support for the claims made.

We thank the reviewer for their consideration of the presentation of our findings. We have currently not provided any figure for the findings presented in this section because these are high level summaries of the overlapping disease effector genes nominated by ourselves and Open Targets. All data in this section is available in Supplementary Material S2, and so in response to this comment we have provided additional reference to that supplementary resource (L295).

"Our eQTLs and ieQTLs also colocalised with 104 of the 137 (75.9%) GWAS loci already reported to colocalise with molecular QTLs in the Open Target Genetics portal (Supplementary Material S2)."

Referee #2 (Remarks to the Author):

I co-reviewed this manuscript with one of the reviewers who provided the listed reports.

Referee #3 (Remarks to the Author):

Alegbe et al. assembled a single-cell atlas comprising 1.8 million cells from blood, terminal ileum, and rectal tissues from 296 healthy donors and 125 patients with Crohn's disease to enable psuedo-bulk cis-eQTL mapping at cell-type resolution. They identified eQTLs for 20,389 genes, and demonstrated that increasing cellular granularity reveals lead eQTL variants are located more proximal to the TSS and in functional enhancers. Through 'interaction eQTL' (ieQTL) mapping, they demonstrate genetic effects attenuated by gut inflammation status. In some example loci, they highlighted heterogeneous effect sizes across cell populations and speculated their functional role in IBD in a cell-state specific manner. Using colocalization analysis, they show cell-type level associations are more likely to colocalize with GWAS loci, nominating effector genes at more than half of all reported IBD loci by their criteria. Finally, they explore whether nominated genes and the appropriate cellular context can inform therapeutic targets through potential drug repurposing.

I think that the data and summary statistics they generated would become valuable resources for the functional understanding of gene regulation in the gut, but more focused analysis and discussion might be warranted for suggesting novel hypotheses for causal gene discovery in the IBD genetics. Counting the overlapping and colocalizing loci in any of the cell types in the gut might not be sufficient to claim their causal role in pathogenesis.

We thank the reviewer for highlighting the value of our data as a resource and for the helpful comment on causal inference. We agree that colocalisation and overlap with GWAS loci are not by themselves sufficient to prove causality, and that further orthogonal evidence is required. Our intention is to nominate *candidate* effector genes and cellular contexts, rather than to assert definitive causal roles. In response to these comments and others (opening comment 'c' by reviewer #1, comment 6 by reviewer #2/3), we have added the following text to our discussion:

L708: "*While experimental perturbation will be required to establish causality for individual genes and pathways, our results provide a focused set of hypotheses for effector genes, cellular contexts, and molecular processes underlying IBD."*

L651: "*Our findings present a novel therapeutic hypothesis that preserving Notch signalling in protective immune contexts, while limiting its activation in others, will have therapeutic benefit in IBD. More broadly, they underscore the value of cell-type-level resolution for dissecting the roles of pleiotropic pathways in complex disease. Further experimental investigation is now required to test these hypotheses and establish causality*"

Major comments:

1. The primary findings they mentioned (e.g., the lead variant is distant from TSS in cell-type-specific eQTL) have been continuously shown in the other studies even with bulk RNA-seq (such as, M. Gutierrez-Arcelus et al. PLoS Genetics 2015, Brown et al. PLoS Genetics 2013, GTEx 2020, etc.). Can the authors acknowledge and discuss

these previous findings and further elaborate on the novel concepts they were able to identify through the usage of single-cell tissue data?

We thank the reviewer for highlighting prior work on the relationship between eQTL distance to TSS and specificity. Previous studies (Gutierrez-Arcelus et al. 2015; Oliva et al. 2020; Natri et al. 2024) compared eQTLs across cell types and showed that those specific to a single cell type tend to be located further from the TSS. Our analysis differs in approach: by structuring our data hierarchically ('All Cells' → Major population → cell type), we show that distance to TSS systematically increases as resolution increases within the same dataset. This hierarchical perspective has not been reported previously, so we regard it as a novel but expected finding that provides reassurance about the robustness of our data.

The major novel findings of our study are that cell type-level eQTLs are relatively enriched in enhancers and, most importantly, are over 3.5-fold more likely to colocalise with IBD GWAS loci than eQTLs detected at tissue-level ("All Cells") resolution (Figs. 2g, 3b). These results demonstrate that cell type resolution provides a more effective route to nominating effector genes and generating disease-relevant hypotheses for testing.

To highlight the existing excellent work in this area and better reflect our novel approach and findings, we have added the following text to the Discussion section of the manuscript:

L593: *"Previous studies have shown that cell-type-specific eQTLs tend to be more distal from the transcription start site* (*Gutierrez-Arcelus et al. 2015; Brown, Mangravite, and Engelhardt 2013; Natri et al. 2024*)*. Here, we extend this observation by analysing eQTLs across hierarchical levels of resolution within the same tissue ('All Cells' → Major population → cell type), showing that distance to TSS systematically increases with cellular resolution."*

2. There also exist single-cell eQTL studies investigating immune cell types (Yazar et al. 2022, Perez et al. 2022 etc.). To what extent do the immune cells from this tissue-specific data lead to novel discovery of IBD GWAS genes that had not been discovered in such peripheral blood studies?

To test the novelty of our findings against sc-eQTL studies of peripheral blood, we compared the disease effector genes we identify with those of Cuomo *et al.,* (2025) (https://doi.org/10.1101/2025.03.20.25324352), provided in Supplementary Table 5 of their manuscript. This dataset is used for two reasons; i) This represents an expansion of the dataset used in Yazar *et al.,* (2022) to 1,925 individuals, and will therefore be better powered to detect associations, and 2) The nomination of disease effector genes for IBD by Perez *et al.,* (2022) is done using TWAS, not colocalisation, and so comparisons between our two studies is confounded by method choice. As Cuomo *et al.,* utilise a PP.H4 threshold of 0.8 and only colocalise with IBD, we applied the same threshold and comparison filter to our findings. Cuomo *et al.,* do not report the colocalising variant in their supplementary data, and so mapping of genes to loci was based on the transcription start site of the gene being <0.5Mb from the index variant of the given GWAS locus. Because Cuomo *et al.,* map eQTLs within

100kb of the gene body, this mapping is analogous to our own for the 97.8% of their effector genes which are less than 300kb in length.

We first compared the set of genes nominated across all of our cell-types and tissues with those of Cuomo *et al*. Of the 255 effector genes we nominate for IBD (PP.H4>0.8), only a minority (82, 32.2%) of these are also nominated by Cuomo *et al*. The effector genes we nominate span 119 IBD GWAS loci, 49 (41.2%) of which do not have any gene nominated by Cuomo *et al.*

Limiting to immune cells, we nominate 117 disease effector genes, with only half (59, 50.4%) of these also being nominated by Cuomo *et al.,* (2025). Even when limiting to immune cells from TI and rectum only, a specific minority of our dataset, the number of disease effector genes we nominate is 79, with 41 (52%) not being nominated by Cuomo *et al.,* (2025). These 79 genes span 58 GWAS loci, with nearly one third (18, 31.0%) of those not having a gene nominated by Cuomo *et al.*

Despite being considerably better powered than ourselves (1,925 individuals compared with 421 individuals), this substantial number of effector genes and loci nominated only by our study speaks to the benefit of obtaining samples from the primary site of disease, and from diseased individuals, in order to nominate disease effector genes. We are grateful to the reviewer for this suggestion, as we believe this comparison further reinforces this benefit. We have therefore included this comparison in the revised version manuscript:

L286: "*Even compared with a sc-eQTL study of peripheral blood mononuclear cells from 1,925 healthy individuals(Cuomo et al. 2025), our study nominates 173 novel effector genes for IBD, including at 49 loci where one is not otherwise nominated (PP_{h4}>0.8). Despite our considerably smaller sample size, we identified 18 loci with effector genes nominated only in our gastrointestinal immune populations. Together, these findings demonstrate the considerable benefit of mapping eQTLs using samples ascertained from the primary site of disease. Notably, at two of these 180 loci, colocalisation was observed only with ieQTLs, illustrating that context-dependent regulatory effects can reveal additional candidate effector genes not detected under baseline conditions.*"

L1133: "*For comparison of disease effector genes nominated in this work with that of Cuomo et al., (Cuomo et al. 2025), as their colocalisation is limited to IBD and with a threshold of PP_{h4}>0.8, we applied the same filtration to our own associations.*"

3. Relatedly, how many of target genes through the colocalization were not identified in previous studies in bulk RNA-seq or single-cell RNA-seq. Can the authors find any features that led to increased discovery using the tissue- and disease-related dataset?

We thank the reviewer for this important follow-up question, which has yielded some significant novel insights. Whereas the previous comment (number 2) focused specifically on the added value of gut immune cells compared with PBMC sc-eQTL datasets, here we interpret the question as addressing novelty across the dataset as a whole, and the features of our design that enable increased discovery.

Novel discovery compared to previous eQTL studies:

To quantify the extent of novel effector gene discovery, we compared our findings with the Open Targets Genetics portal, which collates all publicly available QTL datasets. Our analysis shows that we nominate effector genes at 74 loci where no effector gene had previously been reported, and 257 novel effector genes. We have added this clarification to the manuscript:

L284*: "This includes 74 loci where no effector gene had previously been nominated by colocalisation in the Open Targets Genetics portal (Fig. 3a) and 257 novel disease effector genes."*

Features leading to increased discovery:

We explored whether inclusion of diseased samples enhanced effector gene discovery. In the terminal ileum, where both healthy and Crohn's disease (CD) biopsies were collected, we observed that annotations with a higher proportion of CD samples contributed disproportionately to effector gene discovery. This is illustrated in the new main-text figure (Fig. 3c, shown below), which demonstrates a strong positive association between the proportion of samples for a given cellular annotation that are derived from CD patients (x-axis) and the likelihood of effector gene discovery (a ratio of odds between eGene discovery and disease effector gene discovery, y-axis) (slope=1.31, p=4.68e-09). The greatest enrichment was observed in *ADGRL3*$^{+}$ myofibroblasts, proliferating macrophages, and *KLRG1*$^{+}$ γδ T cells—all cell types with established roles in IBD. This analysis provides further support for the value of including diseased tissue in single-cell eQTL efforts to map disease effector genes.

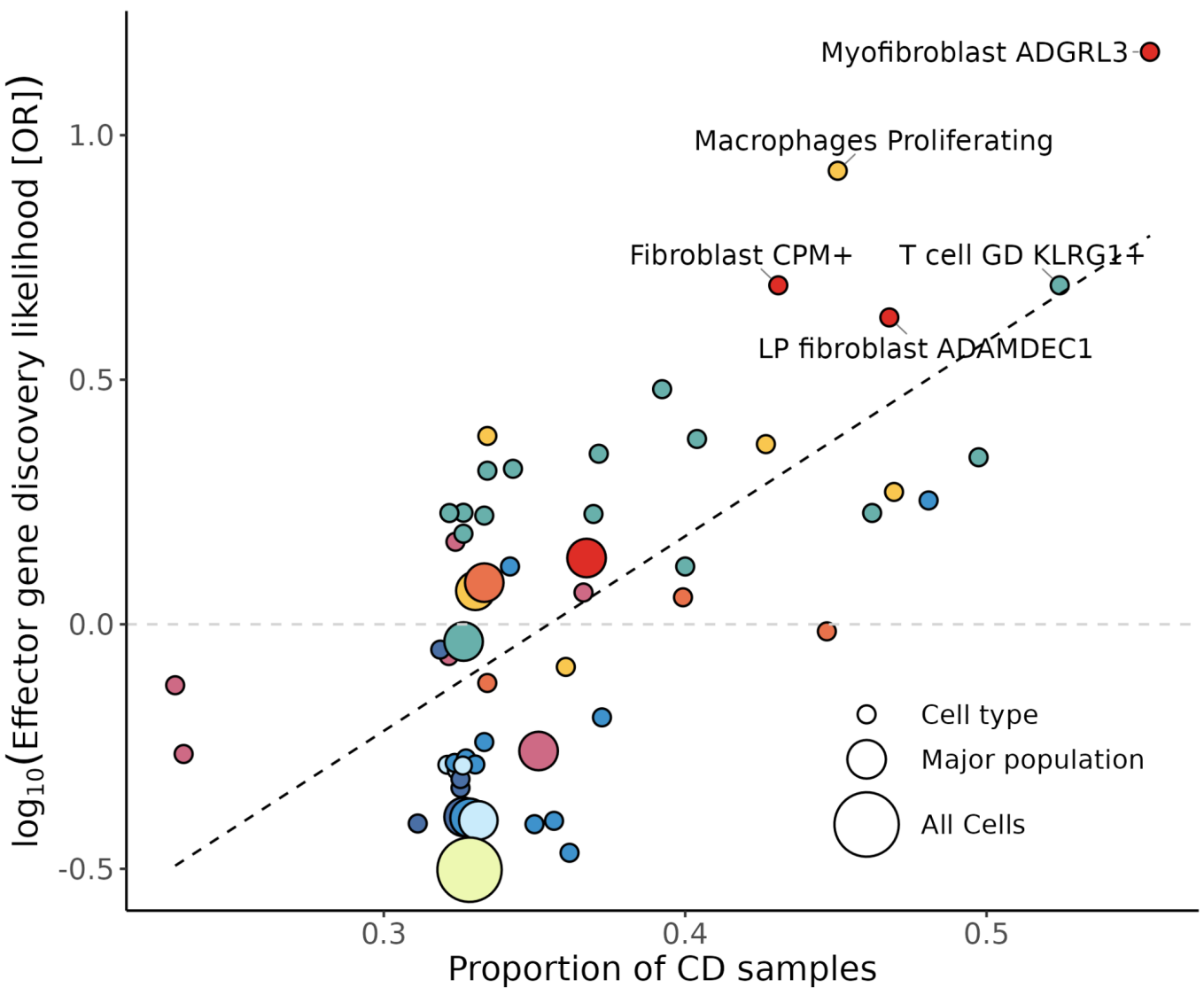

We have also made the corresponding update to the main text of the revised manuscript:

L336: *"To further determine whether the inclusion of diseased samples aided disease effector gene discovery, we compared the proportion of CD samples that contributed to eQTL mapping*

in each cell type of the TI and the likelihood that an eGene detected in this cell type was a disease effector gene (Fig. 3c). Broadly, this shows cell types enriched in CD samples are more likely to harbour regulatory effects that colocalise with disease (univariate linear model slope = 1.31, p=4.68e-09). The greatest enrichment of disease effector genes was observed in ADGRL3⁺ myofibroblasts, proliferating macrophages, and KLRG1⁺ γδ T cells – each with established roles in IBD susceptibility (Lin et al. 2022; Okuno et al. 2002; Garrido-Trigo et al. 2023; Uo et al. 2013; Hu et al. 2022)*. Together, this suggests the inclusion of CD samples has a general positive effect on the discovery of disease effector genes."*

We also tested whether overall gene expression level drives effector gene discovery. Only 27.2% of effector genes showed a correlation between expression level and the variance explained by the colocalising variant, suggesting that expression abundance can contribute but is not a predominant driver. We have added this to the manuscript:

L359: *"The variance in gene expression explained by the colocalising variant was correlated with overall expression level for only 103 effector genes (27.2%, p<0.05), indicating that expression level can sometimes contribute to discoverability, but is not the predominant driver of discovery."*

Together, these analyses show that our dataset provides substantial novel discovery beyond existing eQTL studies, and that inclusion of diseased tissue enhances the power to nominate biologically relevant effector genes.

4. A recent study in colon tissue from IBD patients revealed a subset of eQTL that were not found in non-IBD tissue, and larger effect sizes for eQTL in disease tissue (Nishiyama et al. bioRxiv 2024). For terminal ileum in which they both collected samples from healthy and IBD individuals, have the authors observed heterogenous effect sizes between disease vs. control that could be due to differences in cell state or cell type compositions? The authors should highlight the value of having both sets of samples.

We strongly believe that the inclusion of diseased samples enhances our discovery of disease effector genes. Unlike other common complex diseases, in the case of IBD we are fortunate that while still difficult, it is possible to obtain samples from patients during active stages of disease, as opposed to being restricted to postmortem sampling. We were therefore keen to assess and emphasise the value of obtaining biological samples from disease cases, and believe that we highlight this in a number of ways:

1. **ieQTLs.** We identify over a thousand genetic associations that were only detectable when considering patient level covariates, including disease status and disease severity (L220). The overwhelming majority of these are distinct from non-interacting effects (L227 and response to reviewer #2/3 minor comment 3), and hence, could only be uncovered by sampling from a heterogeneous disease population.
2. **Colocalisation of ieQTLs with GWAS.** At two loci, only ieQTLs, not eQTLs, colocalised with susceptibility GWAS (L292), reinforcing the utility of these effects to understand disease risk.
3. **Positive association between enrichment in disease samples and disease discovery of disease effector genes.** In response to reviewer #2/3 comment 2

(above), we now show there to be a linear relationship between the enrichment of cell types in diseased samples, and the likelihood that disease effector genes are discovered in them (Fig 3c in the revised manuscript). Together, we believe these findings already show there to be a substantial benefit to mapping eQTLs and ieQTLs across both healthy and diseased samples.

In this comment, the reviewer also highlights interesting findings from a study that compares eQTLs identified in healthy and diseased bulk RNAseq samples. This includes an indication that for colocalising effects, shrunk effect sizes are larger in diseased samples than healthy (Fig 4c in (Nishiyama et al. 2024)). Although it would be possible for us to test for replication of this finding in our dataset - by remapping our eQTLs within healthy and disease samples separately, and further investigating whether varied cell abundance across disease status contributes to any differences - we feel that undertaking this analysis is beyond the scope of our work. We believe our manuscript already includes several sets of complex analyses and presume, given the upload of (Nishiyama et al. 2024) onto *bioRxiv* in October 2024, that this paper is under peer review at another journal, and so do not believe inclusion of this analysis into our manuscript would necessarily be beneficial beyond the analyses described above. We are grateful to the reviewer for their suggestion and highlighting this work. Of course, all data required to replicate this analysis in our cohort will be possible for others when our data is made publicly available upon publication, and if the reviewer and editors deem it necessary for publication, we would be happy to conduct these tests in a future round of review.

5. Can the authors clarify the method used for ieQTL analysis and colocalization? There were two terms involving the interaction covariate: I and GT:I. The ieQTL discovery was on the coefficient for I, GT:I, or both? Also, how was the colocalization done with the ieQTL signal? If it is based on the significance of GT:I term, is that valid analysis to test shared causal variant probability between eQTL and GWAS?

We apologise for our oversight and thank the reviewer for picking up on this notable absence in the text of our methods. With respect to "**Can the authors clarify the method used for ieQTL analysis and colocalization",** in our testing of interaction eQTL effects, we employ the following model:

$$Ex_{G,C} \sim GT + I + GT{:}I + PC_{GT} + PC_{Ex_C}$$

Where *GT* denotes the dosage of alternative alleles across samples (the baseline effect of genotype), *I* denotes the interaction variable (i.e the effect of the interacting variable on expression independent of genotype) and *GT:I* denotes the interaction between genotype and interaction variable. The summary statistics we report for both the ieQTLs and colocalisation of ieQTLs are derived from the *GT:I* coefficient specifically. To clarify this, we have made the following update to the methods section of the paper (L1011):

"*GT:I indicates the coefficient for the interaction between genotype and the interaction variable, reported in results.*"

Specifically, approximate Bayes factors for colocalisation were calculated using the effect sizes and standard errors for the *GT:I* terms. Due to the differing distribution of the *GT:I* effect sizes, an altered prior on the true effect size was estimated using a representative subset of

the interaction summary statistics. We have now updated this in the methods of the revised manuscript (L1116):

“For colocalisation between GWAS and interaction eQTLs, approximate Bayes factors were computed from the effect sizes and standard errors of the GT:I term, and the prior standard deviation on the true effect size was estimated as 0.3 based on the distribution of GT:I effect sizes across all nominal results on chromosome 21.”

With regards to answering “**is that valid analysis to test shared causal variant probability between eQTL and GWAS?”,** we agree that it is an ad-hoc application of coloc. To examine whether it is appropriate in practice, we performed a brief simulation, where we simulated independent data for a trait and gene expression, with the same LD structure. We assumed the trait was under genetic control of one variant in the region and expression under control of one variant + an interaction effect with a binary covariate. We considered scenarios where all these variants were the same (denoted “tr expr int”), all different (“tr | expr | int”) or 2 variants the same and one different (eg “tr int | expr” indicates that the variant controlling interaction should colocalise with the trait, but that there is an additional variant in the region controlling average expression). We conducted colocalisation as described above (though without the PC terms) and compared the distribution of the posterior probability of H4 between the scenarios, shown below. This showed that in this last (perhaps unexpected) scenario we may lose power to detect H4 compared to where all three effects are caused by the same variant. Crucially, the median posterior probability of H4 remained low in all other scenarios. Colours indicate whether we should expect colocalisation across each scenario.

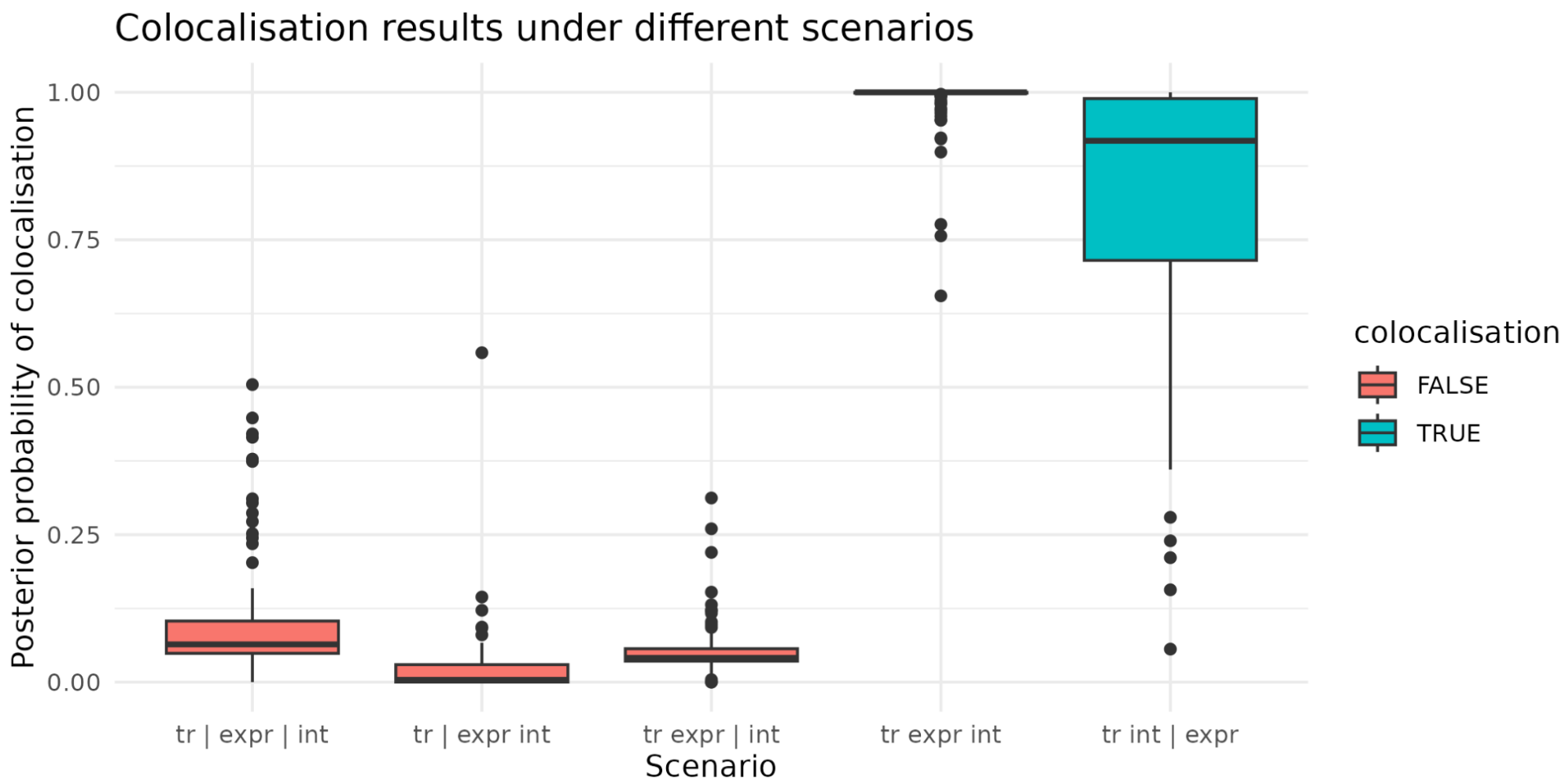

We believe this confirms our colocalisation of ieQTLs is unlikely to introduce false positive associations in this setting, making us confident in our application of this method.

6. While the authors sometimes provided biological interpretation of their findings based on previous literature, relying solely on evidence from any colocalization in any cell types to support a model in which an eQTL mediates disease risk may not be sufficient. For example, linkage disequilibrium can lead to spurious colocalizations. In addition, as we analyze more cell types, we expect to identify more different sets of causal variants, and some may be more likely to colocalize with GWAS, even if it

appears to be random and not in a cell type with additional evidence supporting its role in disease. To ultimately prove the causality of the target gene and variant from this eQTL study, experimental support (e.g., gene KO or variant editing) for selected loci would be necessary.

We thank the reviewer for their critical evaluation of the strength of our evidence. As discussed in response to reviewer #1’s opening comment ‘c’, we acknowledge colocalisation cannot be used to determine causality. We very much agree that functional, experimental evidence is required to confirm the causality and phenotypic consequences of gene dysregulation in various contexts. In light of this, we have added the following text to our discussion:

L651: “*Our findings present a novel therapeutic hypothesis that preserving Notch signalling in protective immune contexts, while limiting its activation in others, will have therapeutic benefit in IBD. More broadly, they underscore the value of cell-type-level resolution for dissecting the roles of pleiotropic pathways in complex disease. Further experimental investigation is now required to test these hypotheses and establish causality*”

L708: “*While experimental perturbation will be required to establish causality for individual genes and pathways, our results provide a focused set of hypotheses for effector genes, cellular contexts, and molecular processes underlying IBD.*”

7. The assumption that a single causal variant underlies the observed association with disease at GWAS loci may not always hold as previously shown. Integrating fine-mapping, such as with the SuSiE framework, can relax the single causal variant assumption by simultaneously estimating distinct signals and quantifying the evidence that each causal SNP is responsible for the observed colocalization. Such analysis could potentially increase colocalization signals with their higher resolution eQTLs (All Cells/Major Population) and provide stronger evidence for their observed colocalizations.

The reviewer raises an important limitation of our approach to nominate disease-effector genes. We agree that relaxing the single causal variant assumption, such as by use of coloc-SuSiE (Wallace 2021), may potentially increase the number of colocalisation events at loci where there are multiple independent association signals. However a known feature of the method we use (coloc, (Giambartolomei et al. 2014)) is that it can still detect colocalisation even in the presence of secondary associations, though with reduced efficiency for stronger secondary effects - Supplementary Figure S8 in (Giambartolomei et al. 2014). This therefore does not discredit those associations we are able to detect using the current method.

The reason we do not apply coloc-SuSiE in our setting is because this only provides an advantage when association signals can be effectively fine mapped. This will not be possible for every association region of every GWAS, and given the vast differences in statistical power across our contexts (Fig 2b), this is likely to be successful in only a sparse, minority of cases eQTLs. Ultimately, this would lead to different tests being performed on eQTLs in well-powered cell types compared with less well-powered cell-types, and across different loci. Consistent with our use, several other sc-eQTL mapping studies also colocalise under the

assumption of a single causal variant (Nathan et al. 2022; Oelen et al. 2022; Bryois et al. 2022; Tian et al. 2024; Natri et al. 2024; Ding et al. 2024; Wang et al. 2025).

To acknowledge the potential benefit of relaxing the single causal variant assumption in the revised manuscript manuscript, we have added the following statement to our discussion (L626):

"*Additionally, while we have performed colocalisation under the assumption that both eQTL and GWAS association are underpinned by a single causal variant, subsequent methods, such as coloc-SuSiE(Wallace 2021), have been developed that relax this assumption. Application of such may uncover additional colocalisation events at loci that contain multiple independent signals, and lead to the identification of additional candidate effector genes*".

8. In the *FUBP1* example, the authors mentioned the opposing directional effect on gene expression that is associated with CD risk loci. This type of analysis can be tricky, since variant(s) in LD, which are causal in one cell type, can affect the sign of the causal variant in the other cell type. Did the authors confirm that they were able to fine-map this locus in all the cell types, and they find the same variant as causal in all the cell types, but still they observe opposing effect directions?

We thank the reviewer for raising an interesting and important question regarding potential confounders affecting the opposite direction of effect for the colocalising *FUBP1* eQTL. To clarify, while we detect colocalisation between a *FUBP1* eQTL and CD susceptibility in Colonocytes (PP.H4=1), the three annotations in which we find the greatest variance in *FUBP1* expression explained by the colocalising eQTL variant are cDC2 cells from the rectum (beta = -1.02, variance explained=0.1243), *ADGRL3*$^{+}$ Myofibroblasts from the rectum (beta=0.81, variance explained=0.1238) and *CD8*$^{+}$ *TGCR2*$^{+}$ Tissue TRM cells from the blood (beta=-0.81, variance explained=0.1238). For this reason, our further analysis into this colocalising eQTL has focused on these three annotations.

In this comment, the reviewer asks whether we have conducted fine mapping of our eQTLs, and whether the same, colocalising eQTL variant is causal (i.e included in fine mapping credible sets for eQTL effects) across these annotations. In theory, such an analysis would indeed allow direct comparison of the eQTL across annotations, however we did not conduct fine mapping of our eQTLs because the vast differences in statistical power across cellular annotations, as shown in Figure 2b, would make the overall efficiency of fine mapping low and very variable. Hence, comparing putative causal variants to answer questions such as these would become unreliable.

In line with this, although we believe the colocalising eQTL is active in these cell types - given the nominal significance of association ($p<0.05$), and relatively large proportion of variance in *FUBP1* expression explained by the colocalising variant (>0.12) - the eQTL does not pass genome-wide significance in these conditions (genome-wide FDR<0.05), likely because of diminished statistical power due to the relatively low abundance of these cell-types. This illustrates the precise scenario which we aim to overcome by conducting the variance explained analysis, where a variant potentially elicits its effect in a particular cell type, but does not colocalise as a result of insufficient statistical power.

In light of these issues, to investigate whether there was any evidence that eQTLs we, GTEx, or Yazar *et al.,* (2022) identify for *FUBP1* (genome-wide FDR < 0.05) could confound the opposite directions of effect we observe across these cell types, we reperformed LD clumping including lead variants from these datasets ($r^2 > 0.5$). In addition, while not detected as genome-wide significant, we also included variants that had the smallest p-value for eQTL effects on *FUBP1* in the three annotations where the colocalising effect was strongest. This analysis included a total of 15 different lead variants, 14 of which were identified in our imputed genotypes, and were ultimately found to form 8 distinct eQTL clumps. Below, we have summarised the linkage disequilibrium (r^2) between the lead variants in our dataset. The boxes along the diagonal indicate the grouping of leads into LD clumps, blue if they contain lead variants from our study, black if not. We also annotated which clump contained lead variants that colocalised with disease susceptibility (orange box). A '*' is placed above the index variant of each clump on the y-axis.

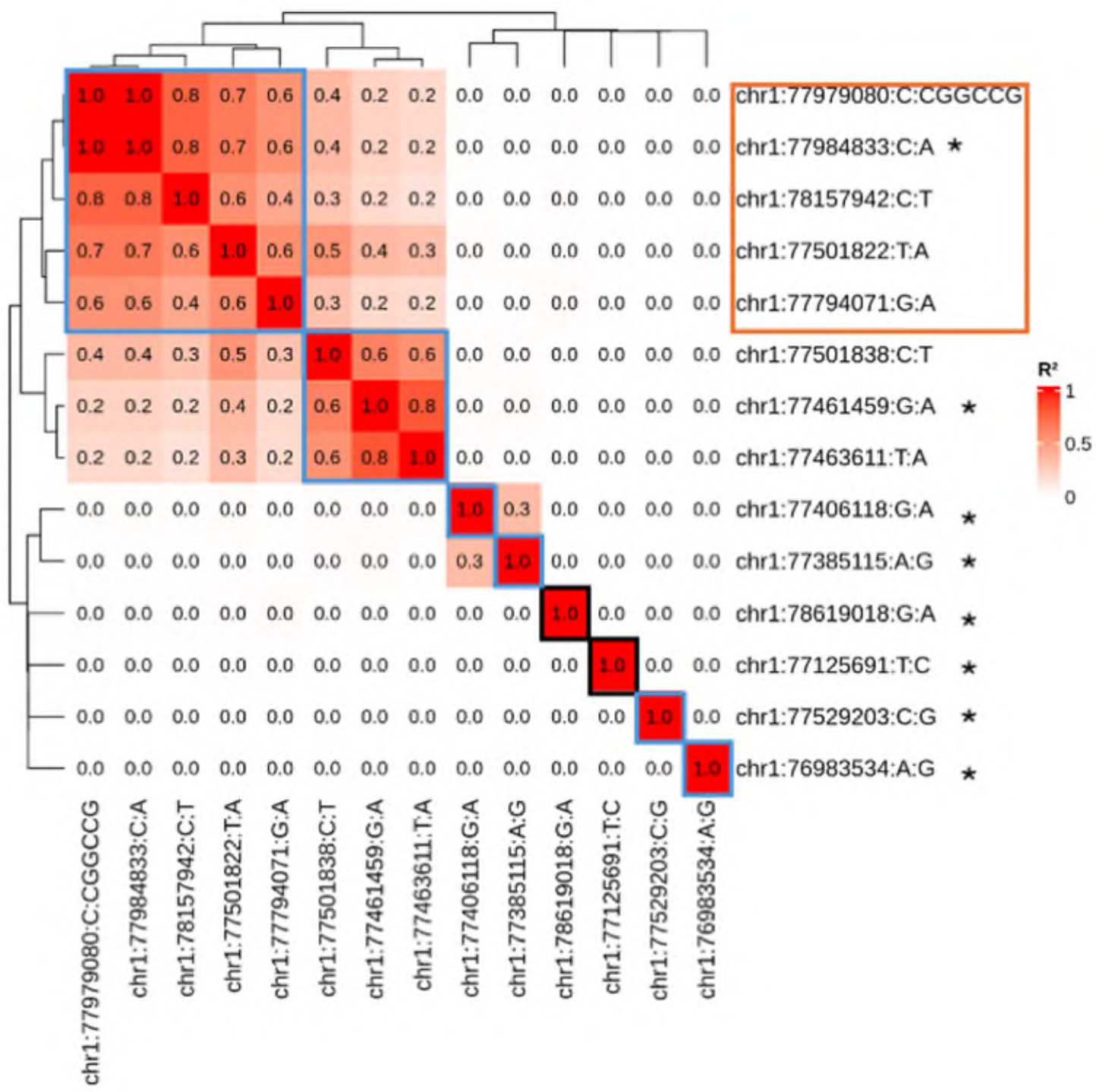

The 'chr1:77406118:G:A', 'chr1:77385115:A:G', 'chr1:78619018:G:A', 'chr1:77125691:T:C', 'chr1:77529203:C:G' and 'chr1:76983534:A:G' variants (bottom right hand corner of the plot) are in minimal LD with the 'chr1:77984833:C:A' colocalising eQTL clump, hence are unlikely to confound our observation. However, while forming two distinct clumps, lead variants from the 'chr1:77461459:G:A' and 'chr1:77984833:C:A' clumps show modest LD. To delineate whether these variants could be responsible for the opposite directions of effect observed across cell types, we compared the primary eQTL associations between the variants within each of the clumps and *FUBP1* expression, limiting to the three annotations where the colocalising eQTL effect appears strongest, displayed below. The horizontal dashed line indicates a nominal p-value threshold of 0.05. The vertical dashed line indicates a beta of 0, and points are coloured by the index variant for the eQTL clump they belong to. It is worth noting that the 'chr1:77979080:C:CGGCCG' and 'chr1:77984833:C:A' are in complete LD in our dataset, and the 'chr1:77501822:T:A' and 'chr1:77794071:G:A' variants are in complete

LD when subset to samples that contribute to the rectal cDC2 analysis, hence the lower number of points observed than what may be expected.

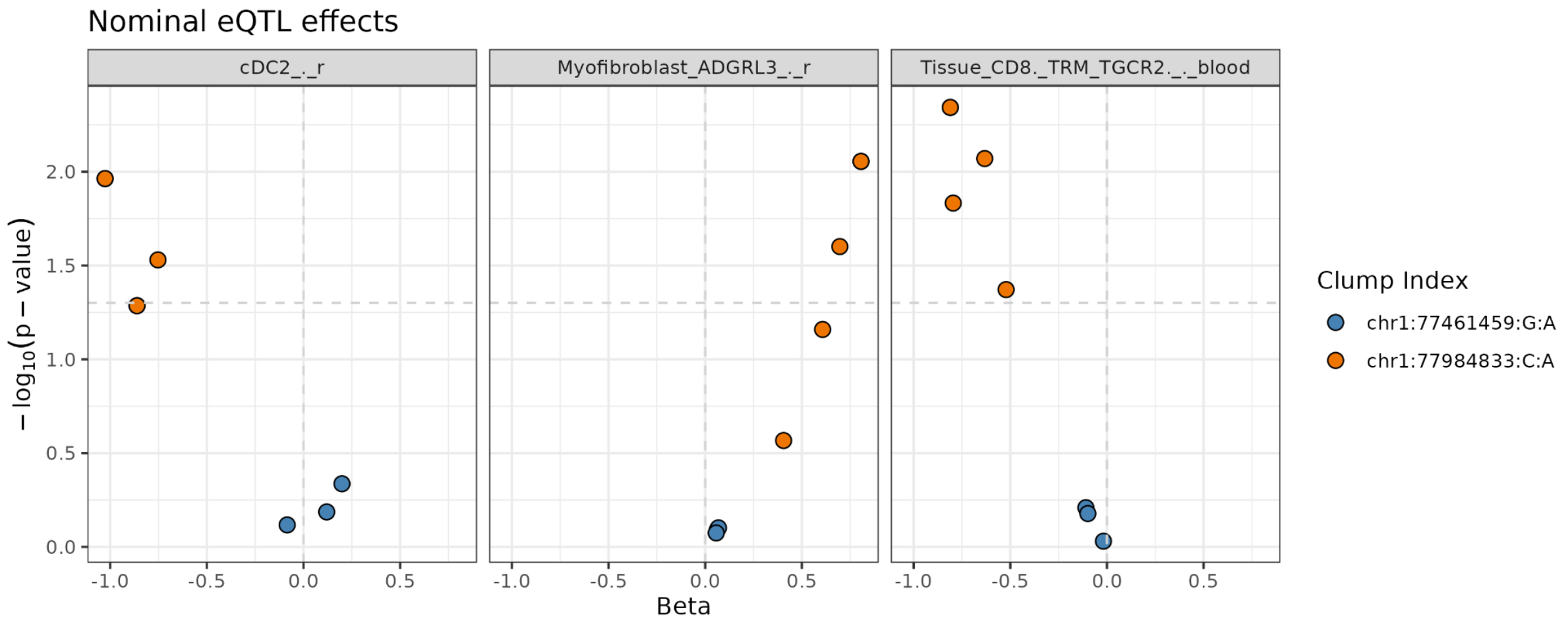

Overall, this indicates that only variants from the ‘chr1:77984833:C:A’ clump, the colocalising eQTL, show any association with the expression of *FUBP1* in these annotations (nominal p-value<0.05). In addition, this also supports opposite directions of effect to be observed in the rectal Myofibroblasts compared with rectal cDC2s and blood *CD8*$^{+}$ *TCGR2*$^{+}$ TRM T cells. To further examine whether the nominal association between variants in the ‘chr1:77984833:C:A’ clump are not influenced by those in the ‘chr1:77461459:G:A’ clump, we also re-tested these associations conditioned on the lead ‘chr1:77461459:G:A’ variant (shown below), again finding only variants from the ‘chr1:77984833:C:A’ clump to show nominal significance.

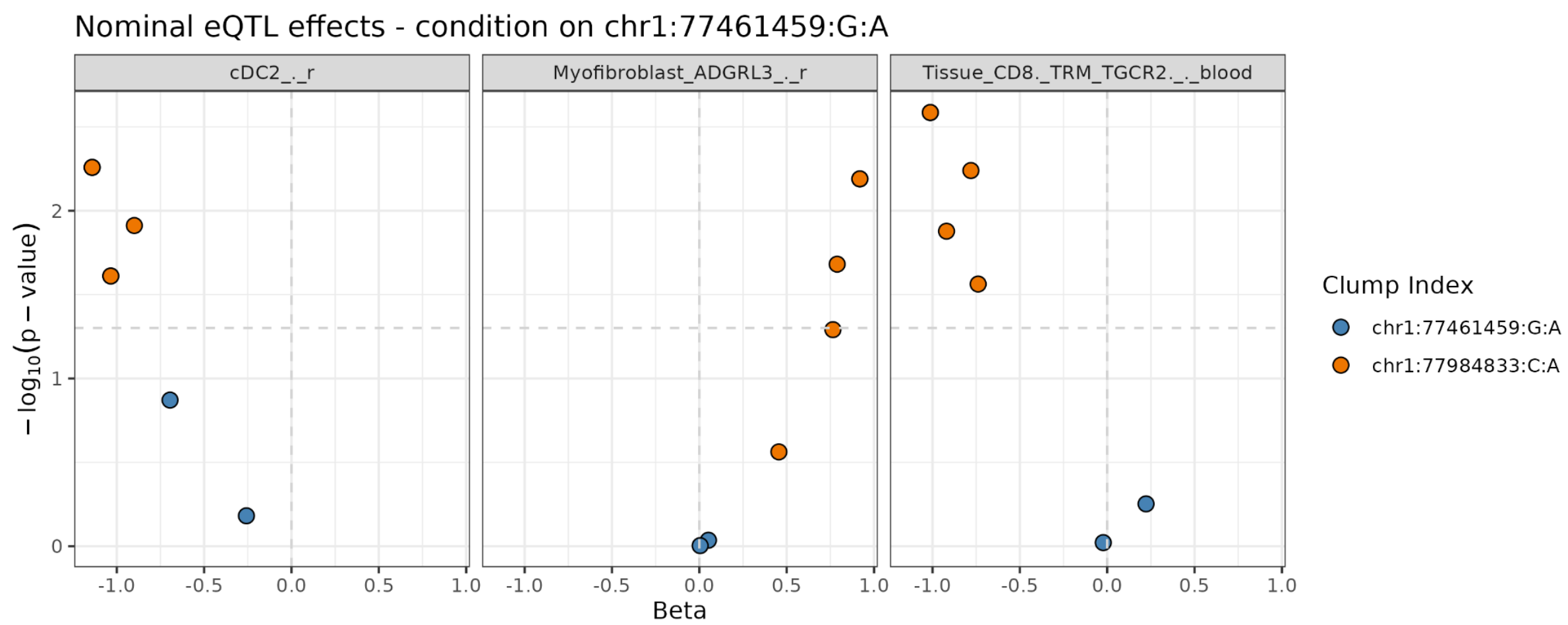

This evidence suggests that the *FUBP1* eQTL, for which we identify colocalisation with CD susceptibility, is unlikely to be strongly influenced by other eQTLs which could be tested in our dataset, and indeed appears to show opposite directions of effect across these cell types. Of course, it is possible that there are other eQTLs that regulate *FUBP1* expression, however, as we find no evidence of these in our dataset, it is difficult to infer their influence on our observation.

All data required to replicate this analysis for any colocalised eQTL effect will be available upon publication. We have therefore chosen to not update the revised manuscript with respect to this further analysis, and hope this satisfies the reviewer's concern.

9. In lines 183-185, the authors state, "To benchmark our findings, we compared them to colocalisations previously reported for IBD loci in the Open Targets Genetics portal, including those based on expression, splicing, and other molecular QTLs." Use of Open Target to compare against their causal gene links can be tricky, since the Open Target includes multiple sources of information for V2G links, which include eQTL but also other sources like pQTL and in-silico predictions. When validating their disease genes, it would be more valuable if they compare their links separately against each individual source to more clearly interpret the additional value of this study.

The reviewer is correct in indicating that Open Targets Genetics utilises multiple molecular assay types and methods in order to nominate disease effector genes for various diseases. This includes colocalisation of molecular QTLs (eQTLs, sQTLs, pQTLs) with GWAS data, the aforementioned V2G, burden tests, variant effect predictions, CRISPR screens, interaction data and literature summaries. In this work, our goal was to nominate effector genes for disease susceptibility using eQTLs, and utilise the Open Targets Genetics database to; 1) quantify how many loci had disease effector genes previously nominated by molecular QTLs, and 2) identify which disease effector genes had been nominated at each locus - benchmarking our own findings against these.

For this reason, while there is some discrepancy between our own and Open Targets Genetics colocalisation methods, to ensure our benchmarking is as unlikely to be confounded by methodological choice as possible, our comparisons with Open Targets Genetics was based on colocalisation of molecular QTLs only. This therefore uses only the QTL data itself and not any *in silico* predictions based on these.

To clarify this detail in the manuscript, we have adjusted the following statement to the methods section(L1129):

"We used the R package otargen (v1.1.5) to download all colocalisations between genes with molecular QTLs and inflammatory bowel disease, Crohn's disease and ulcerative colitis GWAS loci from the Open Targets Genetics portal (accessed January 22nd 2025). We retained any colocalisations with PPH4 > 0.75 and assigned to IBD loci as previously described for our colocalisations."

In addition, there are indeed likely to be cases where variants influence disease susceptibility by acting as an sQTL or pQTL, but not an eQTL, for example. Similarly, in a minority of cases variants may confer disease risk by modification of protein-coding sequences, not expression level. In all such scenarios, our ability to nominate such an effect by eQTLs would be significantly diminished, and by benchmarking our findings against all QTL types, we are likely being conservative in our estimates of the novelty provided by our dataset. In some cases, knowledge of whether disease effector genes had been nominated by non-eQTL-based associations may lead to some interesting findings, but deeply characterising such is not the main focus of our work. Hence, in our manuscript, we have chosen to focus only on whether

genes had been nominated as disease effector genes previously, regardless of the molecular assay type, albeit making the novelty of our study likely a conservative estimate.

Importantly, many of the examples we highlight in the original manuscript (*RASGRP1*, *PSEN2*, *MAML2*) are nominated at loci where there is not any colocalisation evidence in Open Targets Genetics, despite use of all QTL types. We therefore believe these examples provide the greatest degree of novelty, and hence focussed on these in the manuscript.

10. The arguments about drug safety and repurposing seemed largely conjectural and lacking thorough evidence. For example, they mention ITGA4, JAK2, & IL23R eQTLs as direct molecular targets of current drugs, but don't describe whether the eQTL effect aligns with the molecular mechanism like they do for their other examples. They loosely mention some other eQTLs that are in immune pathways modulated by IBD drugs saying the pathways are "active across multiple cell types and tissues." In addition, the arguments on diarrhea which they thought as phenotypically related with IBD as drug side effects of metformin and γ-secretase inhibitors are too speculative, relying solely on a single-gene eQTL result while such effects must involve multiple genes and pathways.

We thank the reviewer for their comments regarding the support our genetic associations may provide for therapeutics. We have chosen to address the multiple points mentioned in this comment separately for clarity.

Firstly, we acknowledge that our mention of colocalisation between *ITGA4*, *JAK2*, and *IL23R* expression with disease susceptibility is not accompanied by any evaluation of the direction of these effects, and apologise for the lack of detail. It is also worth noting that our inclusion of *IL23R* in this list is incorrect, we do not detect a colocalised eQTL effect on this gene (PPH4>0.75), and have therefore removed this from our revised manuscript. With respect to the direction of effects on *ITGA4* and *JAK2*, we have provided variance explained plots for the colocalised eQTL below, along with updated regional manhattan plots for the colocalisation (see response to comment 1d by reviewer #2/3).
For *ITGA4*, in the annotation where the colocalising eQTL explains the most variation in gene expression (blood intermediate monocytes), carriage of the IBD susceptibility allele is associated with increased gene expression. Upregulation of *ITGA4* being associated with IBD susceptibility is consistent with the hypothesis that inhibition of this protein would alleviate IBD phenotypes. While the opposite direction of effect is observed in some mesenchymal and T

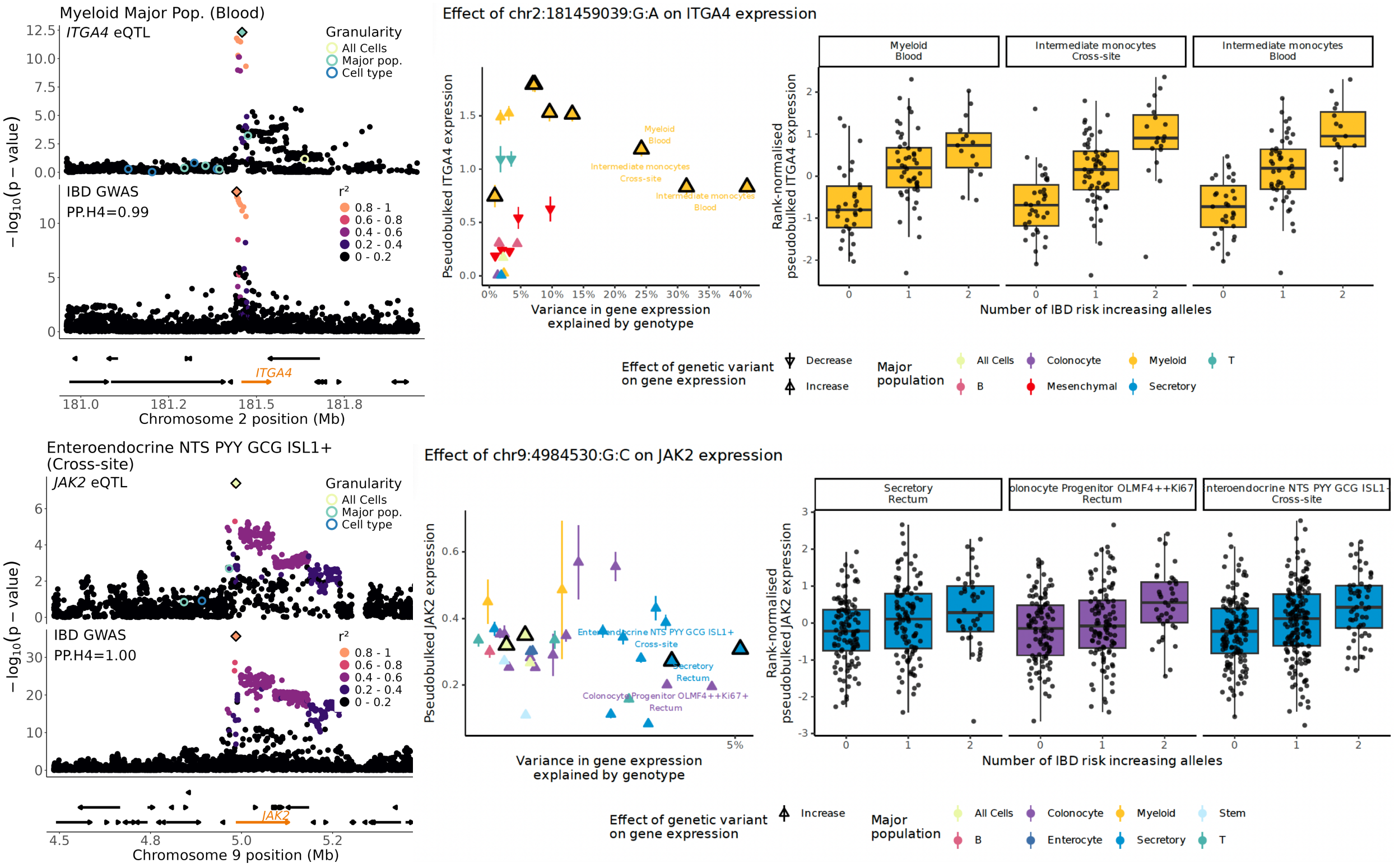

cell subsets, the strength of the eQTL effect is ~25% of that observed in myeloid cells (maximum variance explained=10% and 40% respectively).

Similarly, for *JAK2*, carriage of the IBD susceptibility allele is associated with increased gene expression in all annotations. This is also consistent with the therapeutic hypothesis that inhibition of this protein would alleviate IBD phenotypes. Together, we believe this evidence serves as a positive control in our use of colocalised eQTL effect direction to refine therapeutic hypotheses. We have now included this evidence as supplementary figures and now refer to it in the manuscript (L506):

"*To explore how our effector gene nominations might inform therapeutic strategies, we first asked whether any of the colocalising genes were established or clinically relevant drug targets for IBD. We observed colocalisation between IBD risk and eQTLs for ITGA4 and JAK2, which are the direct molecular targets of vedolizumab and tofacitinib, respectively. The strongest colocalising regulatory effects for these genes indicate their expression is positively associated with IBD susceptibility, consistent with the hypothesis that their inhibition would be therapeutically effective (Supplementary Figure S12)*"

Secondly, we also acknowledge that our original description of immune pathways being "active across multiple cell types and tissues" is somewhat vague. This is mentioned after outlining the cellular annotations in which colocalising eQTL effects for *TNFSF15*, *ITGAL* and *ITGA4* are believed to be strongest. Specifically, our variance explained analysis indicates this to be blood myeloid cells, rectal *CD38*++++ plasma B cells and blood intermediate monocytes respectively (Supplementary Material S3). We believe that greater detail over the specific cell types and tissues in which these effects are believed to be greatest would support the notion that the relevant, currently therapeutically targeted pathways are indeed active across diverse

cell types (myeloid and B cells) and tissues (blood and rectum). We have therefore updated the manuscript text to add this detail, which now reads (L520):

"Notably, the strongest regulatory effects for these genes were distributed across diverse tissues and cell types, including myeloid cells from the blood (TNFSF15), CD38^{++++} plasma B cells from the rectum (ITGAL), and intermediate monocytes from the blood (ITGA4) (Supplementary Material S3). These findings are consistent with understanding that IBD treatments target a number of different immunological pathways, many of which are active across multiple cell types and tissues"

The reviewer also suggests our claims of "**IBD as drug side effects of metformin and γ-secretase inhibitors are too speculative, relying solely on a single-gene eQTL result while such effects must involve multiple genes and pathways**".

It is true that our mapping of drug mechanisms (e.g 'Gamma-secretase inhibitor') to disease effector genes (e.g *PSEN2*) is based solely on the disease effector genes encoding proteins that are directly targeted by the drug (in this case, a component of the γ-secretase complex). However, dysregulation or perturbed function of *PSEN2*, for example, could lead to dysregulation of multiple other genes, and enrichment of disease effector genes amongst these would further support our claims. We did consider incorporating an enrichment test for genes in entire pathways targeted by specific drugs, but were concerned that this would lead to false positive associations. In particular, given the evidence from our variance explained analyses, directions of effect for disease effector genes can frequently be opposite depending on context, making extrapolation of drug targeting effects to a pathway level less reliable.

While conservative, by basing associations solely on genes that encode the direct target of drugs, we believe this leads to much more robust findings. In addition, as described (L549), *PSEN2* dysregulation likely leads to dysregulation of Notch signalling, for which we find multiple other regulators, such as *MAML2* and *ZMIZ1* (L389, L420 respectively). In this specific case, we therefore believe we have orthogonal evidence to support the notion that *PSEN2* inhibition by γ-secretase inhibitors may influence IBD related phenotypes.

Furthermore, while we do not derive this ourselves, potential on-target side effects for both metformin and γ-secretase inhibitors is supported by observational evidence in humans (Bonnet and Scheen 2017; Subramaniam, Joseph, and Babu 2021; Hur 2022; Doody et al. 2013), with the later further supported by functional, experimental evidence in mouse models (Erkert et al. 2024). We acknowledge that a gold standard experiment would be genetic perturbation in these cell types specifically, and have provided an additional sentence in the discussion section to suggest this, in line with another reviewer comment (reviewer #2/3, comment 6) (L651).

"*Our findings present a novel therapeutic hypothesis that preserving Notch signalling in protective immune contexts, while limiting its activation in others, will have therapeutic benefit in IBD. More broadly, they underscore the value of cell-type-level resolution for dissecting the roles of pleiotropic pathways in complex disease. Further experimental investigation is now required to test these hypotheses and establish causality*"

Minor comments:

1. Figures

1a) Figure 2A: It's not readily clear how the varying resolutions produce 384 possible annotations and could be made more apparent.

We thank the reviewer for their evaluation of the interpretability of our overview figures, and apologise for the lack of clarity. Admittedly, we had deliberated about how best to present the various ways in which we mapped eQTLs. To convey the actual number of cell-types in which we tested for eQTL mapping alone (n=86) would require a very large figure, which would quickly become very complicated when overlaid with the collapsing of cell types for major populations and 'All Cells' resolutions, along with grouping within and across anatomical sites. Hence, we chose to rely on a simplified schematic, and aim to convey various composition of cell-types across different anatomical sites (for example Enterocytes in TI, Colonocytes in rectum), and overlay an example cross-site grouping of a myeloid cell-type, the myeloid major population and cross-site 'All Cells'.

However, we agree that this means it is difficult to interpret how this specific grouping ultimately led to 384 potential annotations, and so have updated the accompanying figure legend, which now reads:

"*Schematic to illustrate the ways in which cellular annotations were grouped for eQTL mapping, with example cross-site groupings of a myeloid cell-type, the myeloid major population, and 'All Cells' resolution. Overall, within each anatomical site, eQTLs were mapped at the level of i) cell-types (highest resolution, maximum number of annotations per site = 86), ii) major populations (coarse, middle resolution, maximum number of annotations per site = 9) and iii) the entire sample, referred to as 'All Cells' (lowest resolution, maximum number of annotations per site = 1). By also combining samples across sites within each individual (referred to as 'cross-site'), this leads to 4 site-level annotations and 96 cell-level annotations, resulting in a potential total of 384 unique annotations (see Methods)."*

1b) Figure 3A: The number of loci from Open Target Genetics portal is inconsistent between the figure (n=137) and the text (n=138).

We apologies for the typographical error, this has now been updated in the text to read the correct quantity (L295):

"Our eQTLs and ieQTLs also colocalised with 104 of the 137 (75.9%) GWAS loci already reported to colocalise with molecular QTLs in the Open Target Genetics portal."

1c) Figure 3E: This figure panel is not presented in order, and only introduced later after Figure 4. It may be more clear to restructure the results accordingly.

We have now added a missing reference to Figure 3e, now Figure 3f in the revised manuscript (L375), which means the figure panels are referred to sequentially in the text:

"*Amongst myeloid cell types, dendritic cells (cDC2, and cDC1, and pDC), were three of the four cell types most frequently nominated as the likely effector cell types, with a total of 18*

IBD loci showing the highest proportion of gene expression variance explained in these populations (Fig. 3f)."

1d) Figure 4B: I would recommend changing the colors here, because both the light grey and yellow are hard to read on the axes, but as mentioned earlier, it would be more informative to color by LD here, as well as Figure 5A-C.

On reflection, we acknowledge the issues regarding these figures. We have now replaced the corresponding figure panels with versions where variants for each GWAS or eQTL dataset are coloured by in-sample linkage disequilibrium (r^2) with the lead variant from that region. We have also included annotations of which variants represent additional eQTLs on the given gene (detailed by the outline of points).

Below is an example of our updated version of the figures. Figure legends have also been updated accordingly, with an example also presented below.

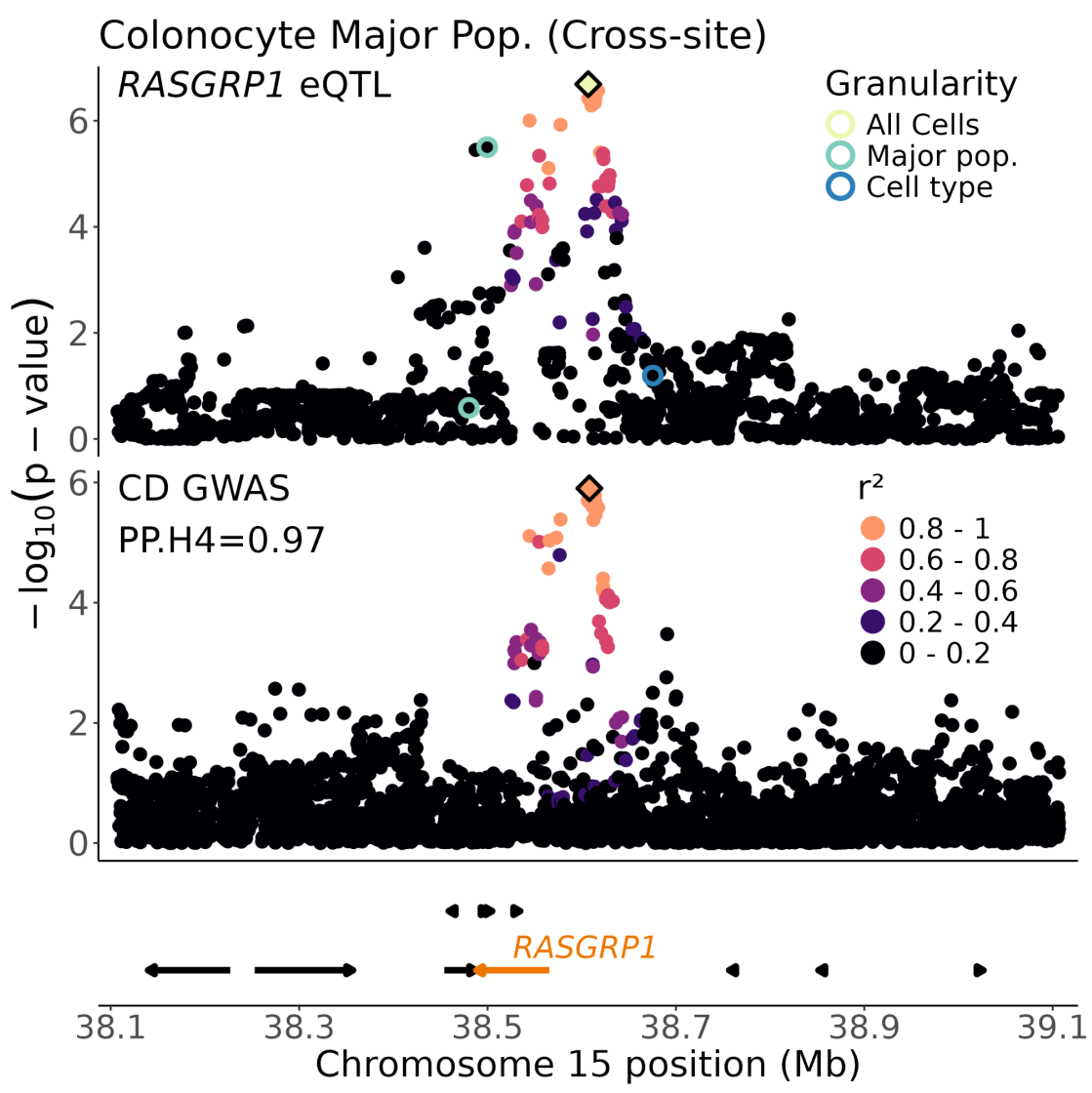

Example legend - "*Significance (-log10[p-value]) of the association between variants with RASGRP1 expression and CD susceptibility. Lead variants for each dataset are presented as a diamond. The lead variant for the eQTL is coloured by the minimum resolution at which the eQTL is detected. All other variants are coloured by their in-sample linkage disequilibrium (r^2) with the index variant from each dataset. Lead variants for other eQTLs are outlined by the colour of the minimum resolution at which they were detected.*"

1e) Figure 6: The spacing along the x-axis needs to be increased, some of the text is overlapping and is illegible.

We have enlarged the text on the Y axis and increased the spacing.

1f) Figure S7: It would facilitate easier interpretation of the results if the authors provided a definition for "maximum specificity of expression" in the figure legend instead of just citing CELLEX.

We have now adjusted the text in the legend for figure S7 to read:

"*Disease effector genes were grouped by the minimum resolution at which their colocalisation was detected (x-axis), as in Figure 2d. The specificity of effector gene expression in each cell type was then calculated using CELLEX (Timshel et al., 2020), and the maximum specificity of effector gene expression is presented (y-axis).*"

1g) Figure S13: I don't know that this figure is showing anything particularly informative. Both the axes and legend are too small to read, and even when zooming in the resolution is too low to read.

Upon reflection, we agree this figure is not informative and have therefore removed it from the manuscript.

2. The authors should add to the methods section on ieQTL mapping whether biological sex as a variable is self-reported or genetically-inferred?

Biological sex of each sample was confirmed for each sample by both genotype (Y chromosome carriage) and gene expression (comparing *XIST* expression to all genes on the Y chromosome). We now include this information in the manuscript (L1012):

"*We tested for interactions with sex, age (in bins of 5 years), disease status (CD or healthy), smoking status, and inflammation status (TI-SES-CD score, as defined previously(Krzak et al. 2024)). The reported sex of each sample was verified by Y chromosome carriage and pseudobulk expression of XIST versus all genes on the Y chromosome.*"

3. In lines 160-162, the authors state, "Although most ieGenes (941; 96.3%) were also eGenes, only 44 ieQTLs (4.2%) were in strong LD with eQTLs (R2 > 0.5), indicating these effects are largely distinct from those observed in conventional eQTL analyses." At a more relaxed LD threshold (R2 > 0.1) what proportion of ieQTL effects are distinct from eQTLs ? Are these analyses all on lead variants?

To first clarify, lead variants for each eGene from all primary (genome-wide FDR<0.05), conditional (genome-wide FDR<0.05) and interaction eQTLs (adjusted p-value < 0.05) were input into LD-clumping. Such approaches have been used to enable the comparison of eQTL signals across conditions previously (Mostafavi et al. 2023). To compare the overlap of ieQTL and eQTLs, we checked how many clumps that contained ieQTLs also contained an eQTL for the same gene, which is only 53 (5.0%) after updating our clumping analysis (see opening statement). In response to this comment, and comment 5 by reviewer #4/5, we have added the following detail to the methods section (L1076):

"This was performed using lead variants from primary eQTLs, conditionally independent eQTLs (genome-wide FDR < 0.05), ieQTLs (adjusted p < 0.05) and lead variants after intersection with GWAS summary statistics to help map colocalisation events to eQTLs"

Next, to test **“At a more relaxed LD threshold (R2 > 0.1) what proportion of ieQTL effects are distinct from eQTLs?” -** we re-derived the LD clumps at multiple, reduced r^2 thresholds and re-quantified the intersection of ieQTLs with eQTLs, displayed below.

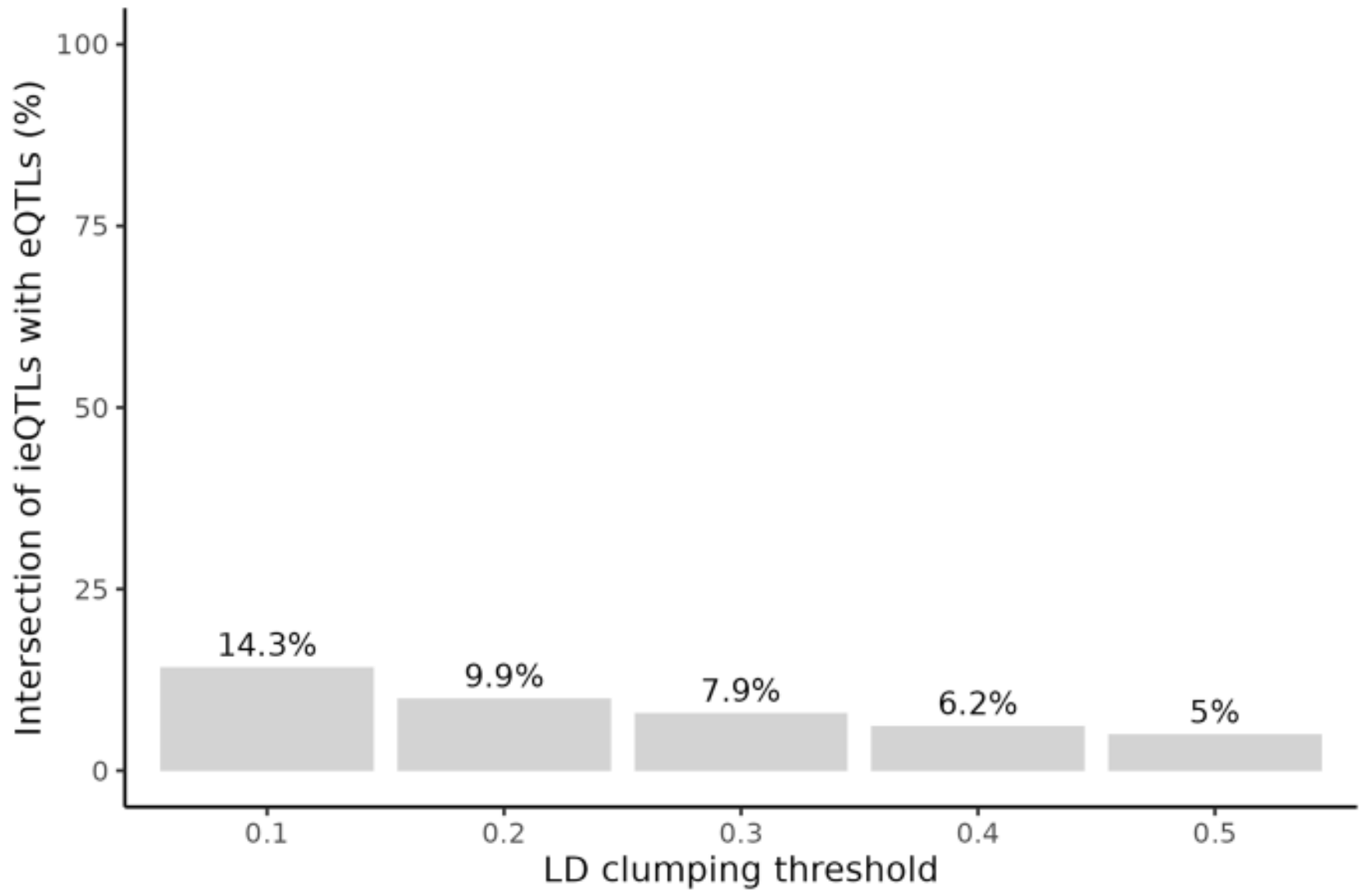

As expected, the proportion of ieQTLs that overlap with eQTLs is increased at lower LD thresholds. However this never exceeds 14.3%, less than 1 in 6, even at a minimal r^2 threshold of 0.1, indicating ieQTLs remain largely distinct from eQTLs. For this reason, we remain confident that ieQTL and eQTL effects on gene expression in our dataset are largely independent of one another.

We thank the reviewer for their query about the overlap of ieQTLs and eQTLs. In light of this supportive new evidence, we have decided to add this figure to the supplement of the manuscript, and the following text to refer to it (L226):

“Although most ieGenes (941; 96.3%) were also eGenes, only 53 ieQTLs (5.0%) were in strong LD with eQTLs (r2>0.5) for the same gene. Even at a much lower threshold of r^2>0.1, only 149 (14.3%) of ieQTLs overlapped with eQTLs (Supplementary Fig. S7), indicating these effects are largely distinct from those observed in conventional eQTL analyses”

4. In lines 215-217, the authors state, “To refine effector cell-type hypotheses, we identified, for each colocalised signal, the cell type in which the lead variant explained the greatest proportion of variance in gene expression.” Is this referring to the lead eQTL variant or the lead GWAS variant at that locus?

We thank the reviewer for raising this important point and apologise for the lack of clarity. We test the variance in gene expression explained by the lead eQTL variant from each colocalisation after intersection with the GWAS variants being compared with. Reviewer #1 also raised a concern over the clarity of this statement in manuscript, which has now been updated to read (L346):

"*To refine hypotheses for the cell types in which colocalised effects may be active, we identified, for each colocalised signal, the cell type in which the lead eQTL variant explained the greatest proportion of variance in gene expression (Methods), and refer to these as 'effector cell types'*"

We have also clarified this in the methods section L1149:

"*To circumvent the influence of varied statistical power in the nomination of cell types within which colocalisation events are active, for each colocalisation, we quantified the proportion of variance in disease effector gene expression (r-squared) that was explained by the lead eQTL variant after intersection with the GWAS variants across each condition.*"

Referee #3 (Remarks on code availability):

A script used for the colocalization was inaccessible to the reviewer. The exact script for QTL mapping was not available, as the repository was for general QTL mapping but not specifically for this study.

We have rectified this by providing the config files necessary to run QTLight in our code repository at https://github.com/andersonlab/IBDVerse-sc-eQTL-code, and by making the colocalisation repository public at https://github.com/andersonlab/snakemake_colocalisation_sceqtl.

Referee #4 (Remarks to the Author):

I co-reviewed this manuscript with one of the reviewers who provided the listed reports.

Referee #5 (Remarks to the Author):

There is significant interest in understanding the molecular mechanisms that underlie genetic associations with disease. While expression quantitative trait loci (eQTLs) derived from bulk RNA-seq have been widely used for this purpose, they have not consistently delivered on the promise of elucidating causal mechanisms. One proposed explanation for this shortcoming is that bulk RNA-seq may mask cell type-specific expression variability that is relevant to disease. In this impressive study, Alegbe, Harris et al. investigate variants linked to inflammatory bowel disease (IBD) and their overlap with cell type-specific eQTLs identified from a large collection of single-cell RNA-seq data derived from blood and intestinal biopsies. This work is both important and timely, particularly as the field considers how best to use functional genomics to interpret complex trait genetics. The authors carefully map eQTLs across diverse cell types and demonstrate significant overlap with IBD GWAS loci, an observation with important implications for explaining the often-limited overlap between eQTL and GWAS signals. Building on this set of effector gene candidates, the authors highlight the roles of Notch and Wnt signaling in IBD pathogenesis and nominate several potential drug targets. Overall, the study is rigorously executed and provides findings of broad relevance to both the IBD research community and the larger field of complex trait genetics. In addition to the scientific insights, the dataset represents a valuable single-cell resource that will be broadly useful. I have not identified any flaws that would preclude publication, and assuming appropriate responses to reviewer comments, I would likely recommend the manuscript for publication.

We would like to thank the reviewer for their thorough evaluation of our work and their overwhelmingly positive response to our approach and study impact.

Major
1. In the two sections of the results implicating Notch and Wnt signaling in IBD, the strength of evidence for prioritizing these pathways over others was unclear. In the Notch section, two of thirteen cDC effector genes (or loci?) are noted to be involved in Notch signaling, but is this more than expected by chance? Was any enrichment analysis performed? Similarly, the Wnt signaling section refers to implicated genetic variation, but it is unclear how the connection to Wnt was statistically established. I did not find a comparable statistical argument supporting Wnt's involvement in the main text. If the authors are making pathway-level claims, it is essential that these be supported with appropriate enrichment or statistical tests relative to a null model.

We thank the reviewer for their critical evaluation of the method by which we highlight potential pathways dysregulated by our disease effector genes. Frankly, we deliberated for a long time about how best to nominate such pathways, and had considered including a formal enrichment analysis as the reviewer mentions. However, there are several limitations to the use of formal enrichment analyses to nominate pathways in accordance with genetic associations, particularly when applied to sc-eQTLs, detailed below:

The sheer number of possible tests: In an exploratory analysis such as the one the reviewer describes, it may seem appropriate to test as many different pathways as possible. To our knowledge, using pathways from sources such as KEGG, REACTOME, MSigDB and others, there are at least 34,000 different gene sets which could serve as reasonable candidates for us to test. Given that we have 251 annotations in which we detect eQTLs, with colocalisation events observed in 245 of these, this ultimately leads to an enormous number of tests being performed, which would result in very little statistical power if one was to correct for all hypotheses being tested. This would likely result in only large, high level immune and cytokine signalling pathways being statistically significant (e.g. immune response, cytokine signalling). Such pathways are already well-associated with IBD, and so would offer little novelty.

Limitations of pathway structure: In many cases, pathways are grouped hierarchically (see REACTOME - https://reactome.org/PathwayBrowser/). This means that the genes comprising higher level pathways are entirely composed of multiple, lower level pathways. Not only does this somewhat arbitrarily inflate the number of tests being performed, but can also make it difficult to statistically evaluate the enrichment of individual pathways with respect to one another.

Limitations of pathway sources: A large proportion of these pathways are manually curated from existing literature and biological knowledge (e.g KEGG), or if molecular evidence is utilised, this is usually based on microarray or bulk RNAseq of cancer cell lines or tumours (e.g MSigDb). It is reasonable to assume that such evidence will likely lead to the aggregation of genes into pathways that do not accurately reflect the case observed in scRNAseq of non-tumour primary tissue, and hence be less accurate in our data. In addition, these also tend to be biased towards pathways observed in cancers or immune cells, further diminishing their relevance to our setting.

Curated pathways may not be amenable to single cell type pathway enrichment analyses: It is also difficult to determine the resolution and method by which it would be most appropriate to perform our testing. For example, the most straight-forward test would be to perform the analysis within each of our cell types. However, the upregulation of a gene encoding a ligand, for example, in one cell-type may have an influence of signalling in a separate cell-type. Related to the derivation of these pathways primarily from bulk studies, this therefore means formal pathway enrichment could plausibly lead to false negatives in some cell-types where a multi-cellular pathway may indeed be initiated or responded to.

Ranking of disease effector genes for gene set testing: For formal gene set enrichment, genes must be ranked by some value in order to determine their relative enrichment. One way in which this could be done is by the posterior probability of colocalisation, or significance of the eQTL, however given the disparity in statistical power we observe across our cellular annotations, we believe these would not be reliable metrics. Another possibility would be to rank disease genes by their eQTL effect size, however given the aforementioned caveat of the inclusion of genes in pathways irrespective of their direction of effect, this is also likely to lead to unreliable findings.

It is likely that for these reasons, performing formal enrichment analyses of nominated effector genes is uncommon, with several examples of high impact work not relying on such to interpret their findings (Hatzikotoulas et al. 2025; Cuomo et al. 2025; X. Meng et al. 2024; Lammi et al.

2025; Emani et al. 2024). Our previous GWAS of IBD highlighted several integrin genes to be dysregulated by susceptibility variants in the absence of formal pathway testing (de Lange et al. 2017). Therapeutic inhibition of integrins is a widely used and successful treatment of IBD, and so this illustrates meaningful and clinically important insights can be drawn from associations without the need for formal enrichment testing.

Our approach was to therefore carefully examine the potential disease-associated functions of newly nominated disease effector genes at previously uncharacterised loci. It is at these regions where we believe our work offers the greatest benefit in terms of refining mechanistic hypotheses. Especially when also considering the cellular context in which these effects appeared to be strongest (relying on our variance explained analysis), we observed an accumulation of Notch signalling regulators in dendritic cells, and Wnt regulators in epithelial cells, drawing us to these pathways and cell-types specifically. It was never our intention to suggest these are the pathways most enriched with effector genes, above and beyond well-characterised mechanisms of susceptibility.

Because formal enrichment analysis was not the method by which we arrived at these pathways, we feel it would be disingenuous and misleading to retrospectively apply such an approach in the revised version of the manuscript. However, we appreciate it would be beneficial if the reader could evaluate the statistical evidence for which these pathways may be enriched with disease effector genes relative to a null model, as the reviewer suggests. We have therefore performed a targeted over-representation test of canonical Notch and Wnt signalling pathways (GO:0007219 and GO:0016055) in our disease effector genes against the background of all eGenes we identify using clusterProfiler v4.10.1(Wu et al. 2021). We find both pathways to be significantly over-represented in our disease effector genes (pvalues=0.026 and 0.015 respectively), and have therefore, in conjunction with evidence provided by additional comparisons in response to comment 3 by reviewer #4/5, made the following corresponding changes to the revised manuscript.

L385: *"Of the 13 IBD loci where the strongest colocalising regulatory effects were observed in cDCs, two nominate genes involved in Notch signalling. When considered across all disease effector genes, Notch pathway members are significantly enriched (p = 0.026): whereas Open Targets Genetics and Cuomo et al. (Cuomo et al. 2025) previously nominated four genes in this pathway, we identify six additional Notch regulators"*

L457: *"Previously nominated effector genes by Open Targets Genetics and Cuomo et al., (Cuomo et al. 2025) include only six Wnt regulators, however we nominate an additional fourteen, making this pathway over-represented amongst our disease effector genes (p=0.015)."*

L1156: *"To assess the evidence for enrichment of Notch and Wnt pathway regulators in our disease effector genes, we performed a gene ontology over-representation test of 'Notch signalling pathway' (GO:0007219) and 'Wnt signalling pathway' (GO:0016055) in our disease effector genes against a background of all eGenes using clusterProfiler v4.10.1(Wu et al. 2021). This was performed using default parameters except minGSSize=3, pvalueCutoff=1, qvalueCutoff=1. These gene sets were also used to quantify the number of disease effector genes previously and presently nominated in these pathways."*

We once again thank the reviewer for raising this concern because the manuscript has been strengthened as a result.

2. The observation that over 75% of GWAS colocalizations overlap with those from Open Targets is encouraging, but further validation of identified eQTLs would strengthen confidence that these eQTLs are not dataset-specific artifacts. Are there existing datasets that can be used to assess the overlap of these cell type-specific eQTLs with prior observations? While this dataset is unique, it may still be possible to show concordance for a subset of eQTLs. For instance, bulk RNA-seq-based eQTLs from GTEx could serve as a point of comparison for the 'all cells' analysis that aggregates data across the terminal ileum tissue. The blood eQTLs could perhaps be validated with other large-scale blood single-cell resources (Yazar et al.). While I recognize that similarly powered resources may not exist for validating interaction eQTLs, additional benchmarking of standard eQTLs would help support the robustness of the findings.

As the reviewer suggests, there are no other sc-eQTL studies of the terminal ileum, rectum, or blood of CD patients, making our dataset unique. This makes it inherently difficult to infer whether our findings are dataset-specific artefacts, or are true associations not detected previously because of our advantageous size, sampling and methodology. However, we believe we already provide evidence that supports our associations are likely reliable and not artefactual:

1. Many of our disease effector genes are shared with Cuomo *et al.,* (2025) (see response to comment 2 by reviewer #2/3), and Open Targets Genetics.
2. We replicate the known relative enrichment of bulk eQTLs (approximated by our 'All Cells' annotation) in promoters rather than enhancers (Fig 2g), seen previously (Mostafavi et al. 2023).
3. Colocalisation is enriched at the cell type level (Fig 3b), which is very unlikely to be observed across so many loci if these associations were enriched with false associations.

However, to further quantify how well our eQTLs are captured by previous work, we computed π1 statistics (Storey and Tibshirani 2003) between eQTLs from each of our cellular annotations with bulk eQTLs from GTEx v8 tissues and sc-eQTLs of PBMCs of Yazar *et al.,* (2022). Yazar *et al.,* (2022) was utilised instead of Cuomo *et al.,* (2025) because summary statistics are currently not available for the latter. We believe this analysis enhances the reliability of our findings, and have therefore provided this evidence in the revised manuscript in the form of a Supplementary Note. For convenience, we also provide a brief summary of this evidence below:

Capture of our eQTL effects in GTEx

Firstly, eQTLs detected in the majority of our annotations were well-captured by at least one GTEx tissue, with a median maximum π1 for our annotations of 0.77 (Supplementary Figure S16), shown below (a). In addition, at the 'All Cells' level, the GTEx tissues that best captured eQTLs from each of our tissues were the ones that are expected to be most biologically similar - our blood 'All Cells' eQTLs with GTEx blood (π1=0.79), our TI 'All Cells' eQTLs with GTEx

blood (π1=0.62) and transverse colon (π1=0.59), and our rectum ‘All Cells’ eQTLs with GTEx thyroid (π1=0.604) and transverse colon (π1=0.603) (Supplementary Figure S16), (b) below.

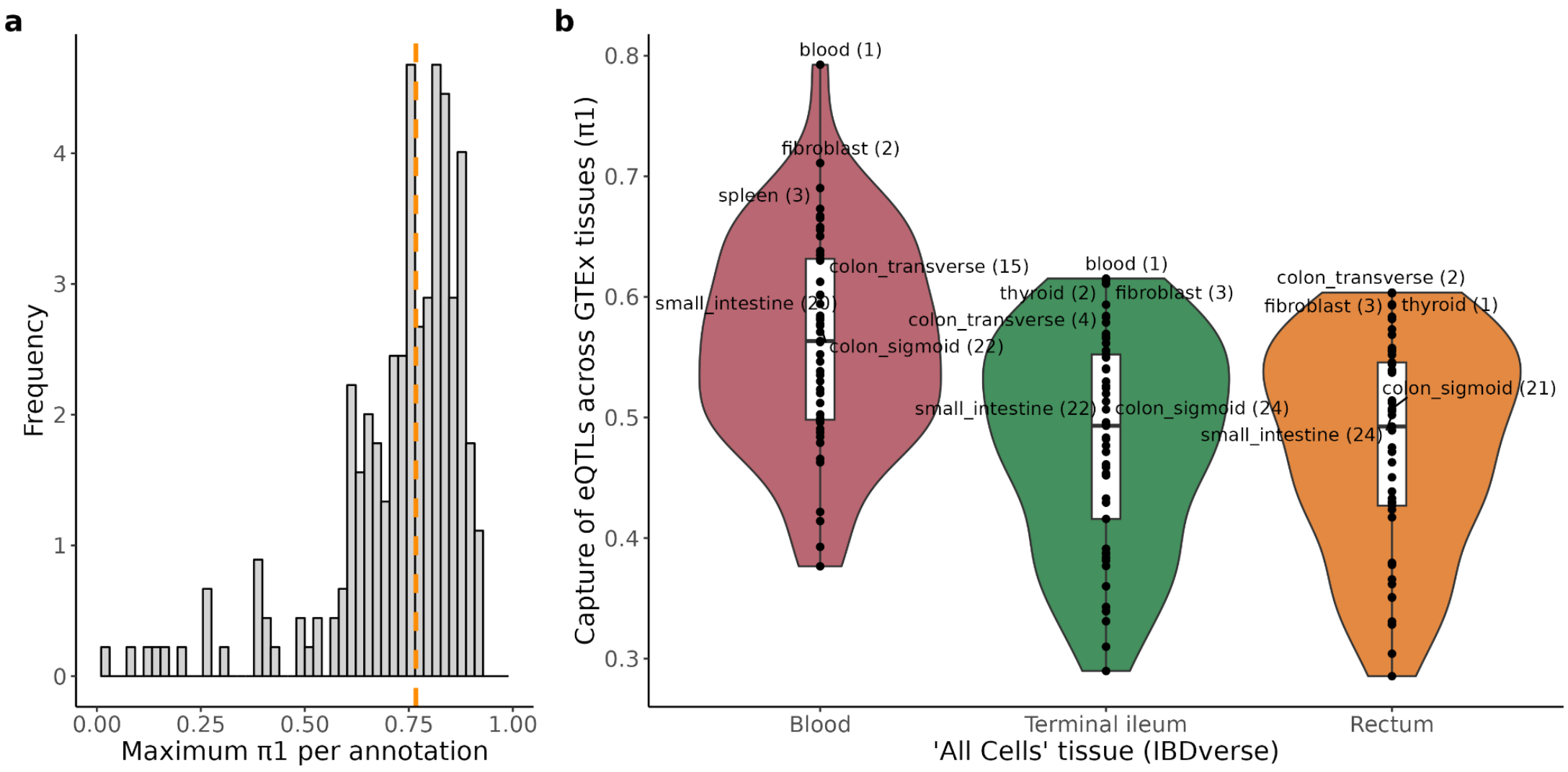

In addition, we also computed how well eQTLs we identify at each cellular resolution, rather than specific cellular annotation, were captured by each GTEx tissue. eQTLs detected at the cell-type level were significantly less well captured than those detected at the major population and ‘All Cells’ level (median π1=0.2, 0.25 and 0.4 respectively, paired wilcoxon p-value=1.1e-14 and 3.6e-15 respectively). We have now included this in the main figures of the manuscript (Fig 2e), and is shown below (e) alongside neighbouring panels.

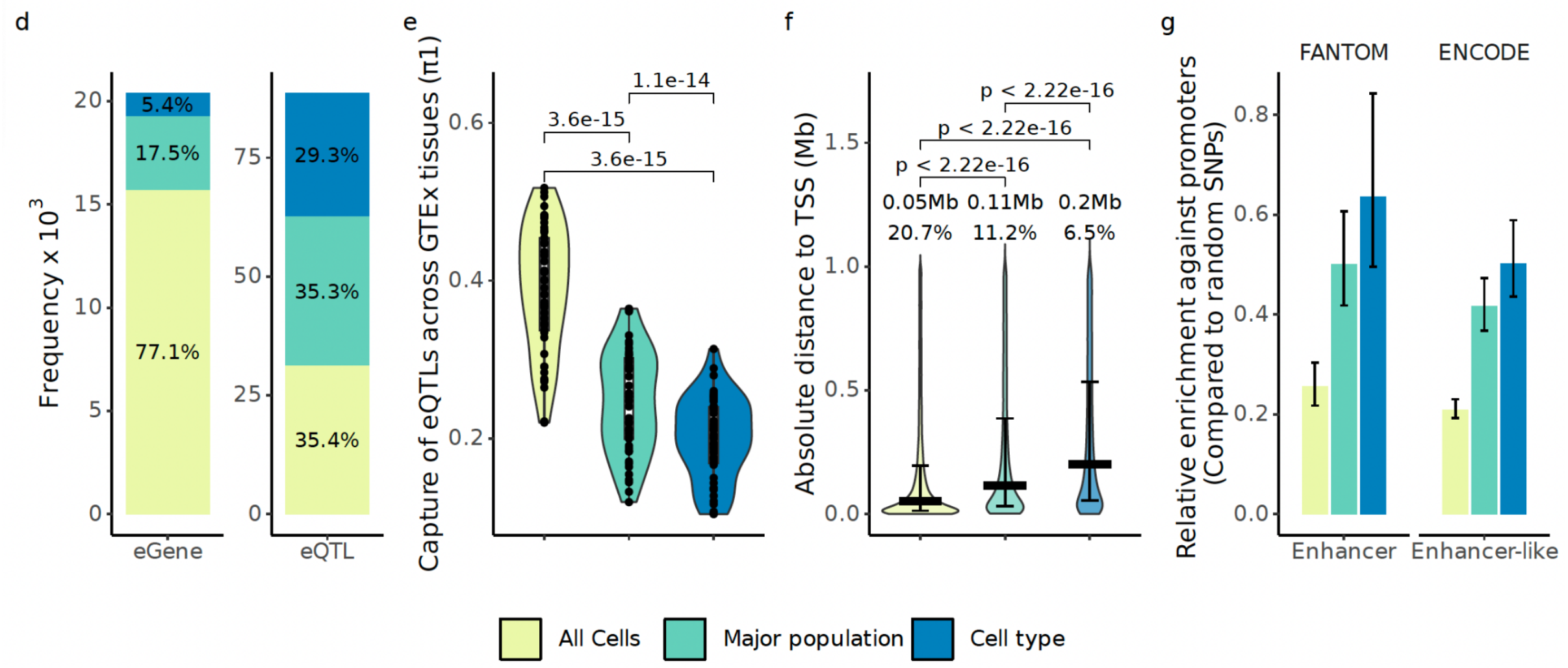

Capture of our eQTLs in Yazar et al., (2022)

Capture of eQTLs detected in cell types from our immune major populations by Yazar *et al.,* (2022) cell types was high, and generally greater than non-immune major populations (median max-π1 range=0.71-0.93 compared with 0.62-0.7 respectively) (Supplementary Figure S17a), (a) below. To test whether the Yazar *et al.,* cell-type that best captured eQTLs of each of our cell types was biologically similar, we grouped Yazar *et al.,* into broad major populations analogous to our own based on nomenclature (see update to Methods below). We then tested

how often the major populations matched for our immune cell-types and the Yazar *et al.,* cell type that best captured these eQTLs Supplementary Figure 17b, (b) below. Major population annotations matched 76.3% of time, significantly better than might be expected by chance (Cohen's kappa test=0.6, p=2.1e-8) and were particularly well-matched for eQTLs detected in our T-cell populations (F1 score=0.84), Supplementary Figure 17c, (c) below.

In light of this supportive evidence, we have made the following changes to the revised manuscript:

L193: "*While many of our eQTLs are also captured by bulk eQTLs from GTEx(GTEx Consortium 2020) or peripheral blood sc-eQTLs from Yazar et al.,(Yazar et al. 2022), as quantified by the π1 statistic (Methods, Supplementary Note), our cell type eQTLs were much*

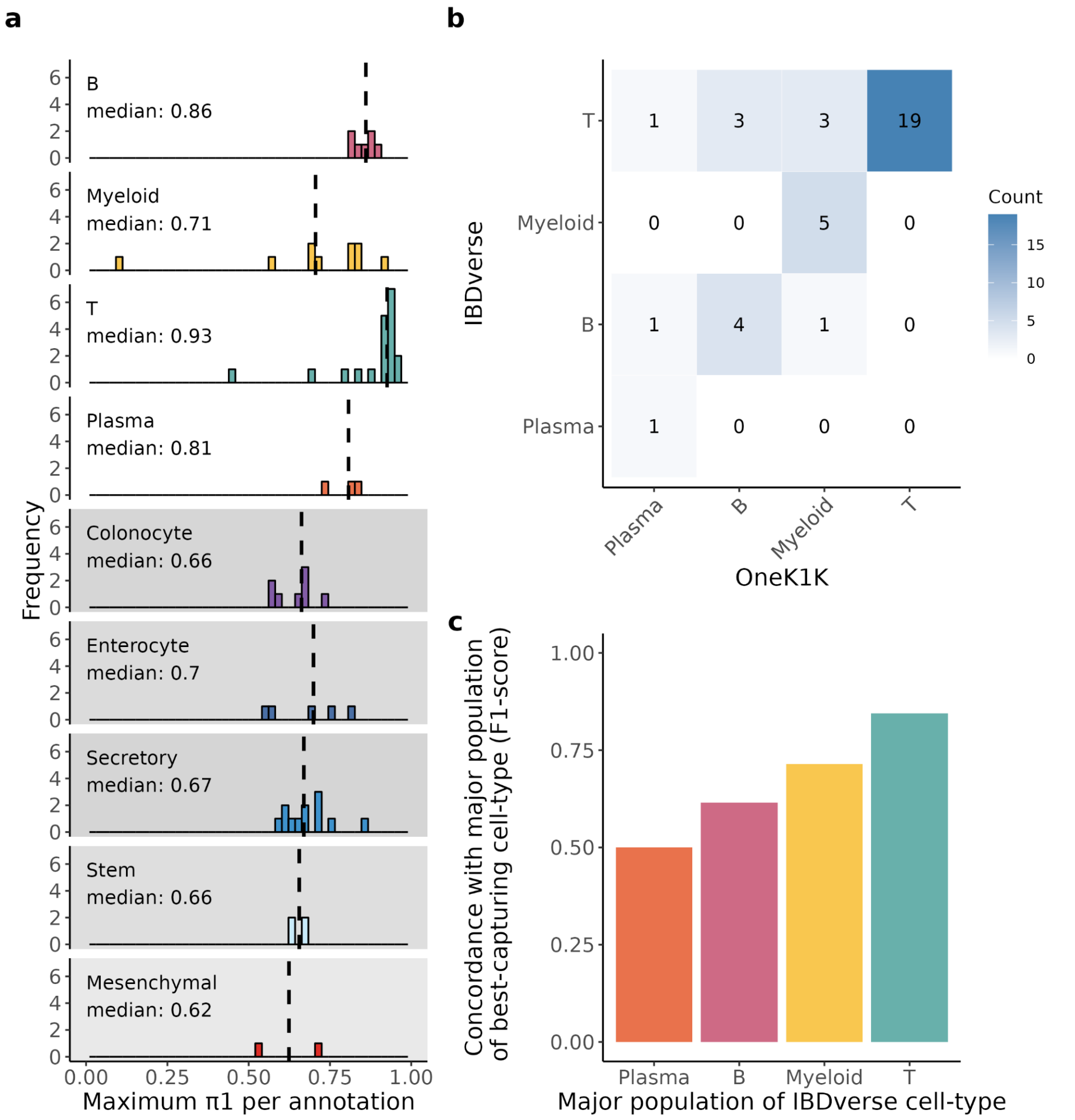

less likely to be found previously than major population or 'All Cells' eQTLs (median π1 with GTEx tissues=0.2, 0.25 and 0.4 respectively, maximum p for comparison across resolution=1.1e-14) (Fig 2e)."

L1090: "***Inferring the capture of our eQTLs in other studies***

Harmonised nominal summary statistics for eQTLs from every GTEx v8 bulk RNAseq tissue(GTEx Consortium 2020) and sc-eQTLs for peripheral blood mononuclear cells of Yazar et al.,(Yazar et al. 2022) were downloaded from eQTL catalogue (https://www.ebi.ac.uk/eqtl/,

study IDs QTS000015 and QTS000038 respectively). For eQTLs detected in each of our annotations, we then calculated the enrichment of these associations amongst low p-values, π1(Storey and Tibshirani 2003), in each external dataset using qvalue v2.34.0(Storey et al. 2023). This was also performed between eQTLs detected at each resolution in our dataset with eQTLs from GTEx tissues. For comparison with Yazar et al.,(Yazar et al. 2022) cell-types were grouped into major populations based on nomenclature (B - intermediate, memory, naive; Myeloid - CD14_Mono, CD16_Mono, Platelet, cDC2, pDC; T - CD4_CTL, CD4_Naive, CD4_TCM, CD4_TEM, CD8_Naive, CD8_TCM, CD8_TEM, MAIT, NK, NK_CD56bright, NK_Proliferating, Treg, dnT, gdT; unknown - HSPC, Haematopoietic stem; Plasma - Plasmablast). A detailed summary of these comparisons is shown in the Supplementary Note."

L1191: "*The pipeline used to perform QTL mapping is available at https://github.com/wtsi-hgi/QTLight, for testing eQTL replication rates at https://github.com/andersonlab/pi1_pairs,...*"

3. The manuscript would benefit from a stronger discussion of how these findings relate to other large-scale single-cell eQTL datasets that assess GWAS overlap. For example, Yazar et al. examined the intersection of immune traits with single-cell eQTLs in blood. Do the current findings corroborate or extend what has been described in such studies? In addition, are there prior reports that support a role for Notch or Wnt signaling in IBD genetics? Connecting the results to prior literature more directly would help readers interpret the novelty and robustness of these conclusions.

In addressing this comment we believe we now incorporate evidence that strengthens support for our findings, and so are grateful for the suggestions. For clarity, we have chosen to address this comment in two parts.

Firstly, with respect to the question "**Do the current findings corroborate or extend what has been described in such studies?"** - Early in our analysis we reasoned that assessing any putative benefit for our approach to identify disease effector genes would be of significant interest to the immune mediated disease research community. To address this, we benchmarked our findings against the most comprehensive publicly available dataset, Open Targets Genetics (Fig 3b). In addition, as part of our response to comment 2 by reviewer #2/3, we have performed a further, detailed comparison of our data specifically to that of Cuomo *et al*., (2025), the largest sc-eQTL study of PBMCs to date (a follow-on study to Yazar et al).

Several of our disease effector genes, including *MAML2*, *RASGRP1* and *MYC*, are critical regulators of Notch or Wnt signalling and have not been nominated previously. We therefore refer to these specific examples as 'novel' (L340, 390, 411). However, to comprehensively assess whether our findings corroborate, or extend previous evidence regarding the involvement of these pathways in IBD risk, we intersected all known disease effector genes from Open Targets Genetics and Cuomo *et al.,* (2025), in addition to our own, with designated Notch / Wnt pathway genes (used in our response to reviewer 5, comment #1), shown below. This shows we more than double, and even triple, the number of disease effector genes nominated for these pathways, colocalising an additional 6 (150%) and 14 (233%) Notch- and Wnt-associated disease effector genes respectively.
Secondly, with respect to the comment - "**Connecting the results to prior literature more directly would help readers interpret the novelty and robustness of these conclusions"**

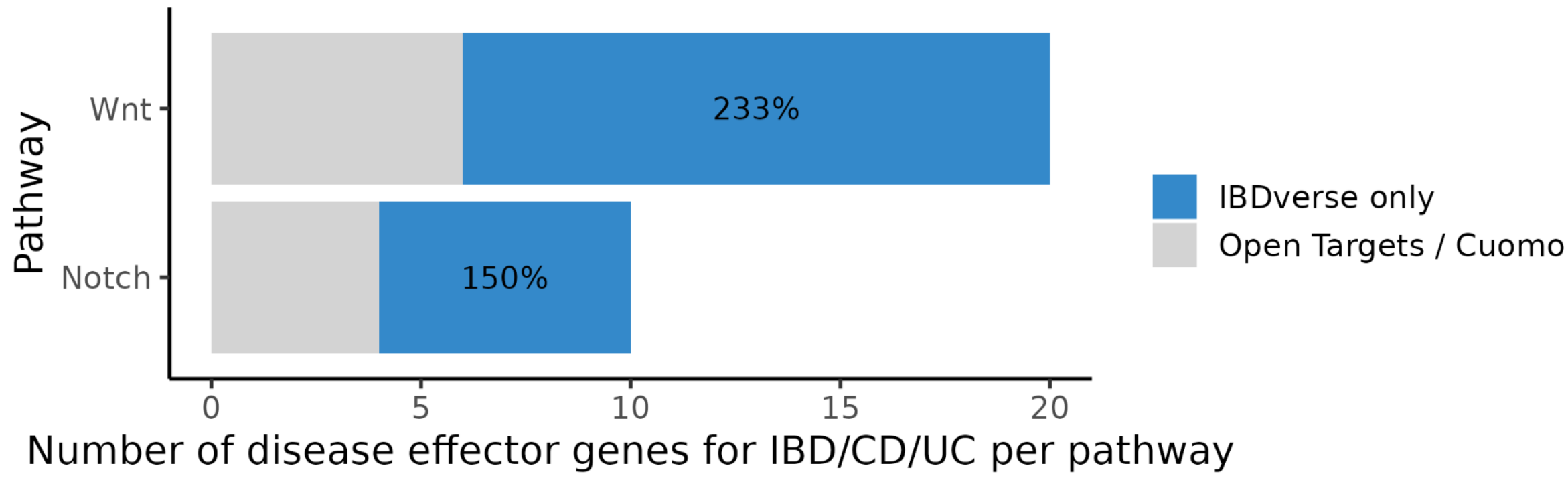

- for brevity in our original submission we did not extensively detail the knowledge-base for Notch/Wnt signaling in IBD, but agree that this would be helpful to give further context to our work. As such, we discuss previous work for broader relevance below:

In addition to the evidence for colocalisation of common regulatory variants, several genes that harbour rare mutations associated with monogenic forms of IBD also encode both direct and indirect regulators of Notch signalling. This includes; *ADAM17*, *IL2RA*, *ITCH* and *TGFBR2* (Blaydon et al. 2011; Uhlig et al. 2021; Bolton et al. 2022; Ouahed et al. 2020; Pazmandi et al. 2019; Nambu et al. 2022). None of these genes have been associated with colocalising eQTL effects previously in Open Targets Genetics, however, we identify colocalisation between an eQTL regulating *TGFBR2* expression and IBD for the first time (PPH4=0.83), shown below.

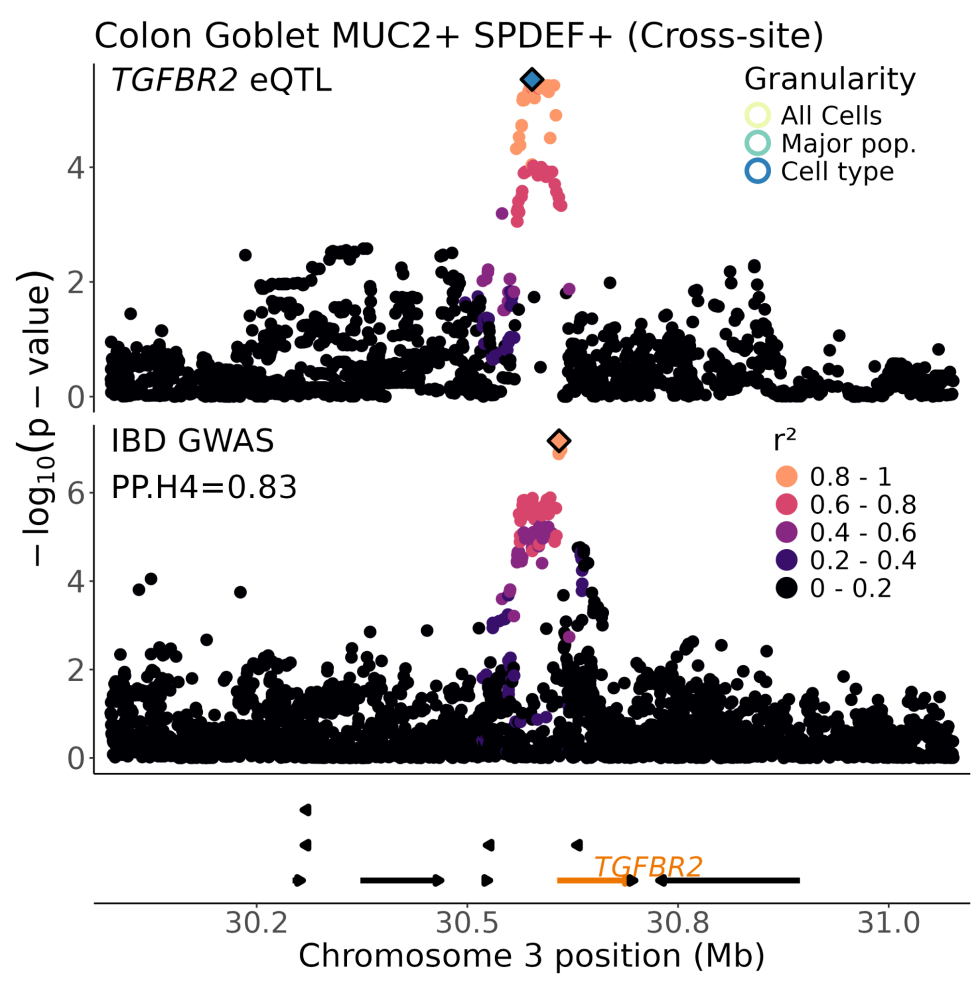

Furthermore, and in keeping with our own data, findings from murine models of IBD suggest that Notch signaling can have both protective and disease-promoting effects depending on cellular context: Epithelial expression of the critical Notch effectors *RBP-J, NOTCH1* and *HES1* is required to prevent loss of barrier function and predisposition to colitis (Obata et al. 2012; Dunkin et al. 2018). Conversely, circulating human CD1c^{+} DCs upregulate Notch ligand *DLL4* in response to TLR stimulation and in graft versus host disease, are associated with augmented cytotoxic Th1 and Th17 differentiation (L. Meng et al. 2016).

Similarly, one of the most common causes of very early onset IBD (<6 years old) pertains to Wnt signalling - namely, loss of function mutations in *XIAP*, a E3 ligase that regulates *WNT*

transcription via ubiquitination of TLE co-repressors (Nambu et al. 2022; Uhlig et al. 2014), with *XIAP* mediated loss of ubiquitination also abrogating signalling by NOD2 (Goncharov et al. 2018), another canonical monogenic IBD gene. One of the most crucial functions of Wnt signalling in intestinal homeostasis relates to epithelial regeneration and repair. Here, stromal and Paneth cells at the crypt base secrete Wnt and Wnt-potentiators (e.g. R-spondin) conferring high Wnt pathway activity, with ileal CD patients demonstrating lower levels of the Wnt-signaling pathway transcription factor Tcf-4 independent of inflammation (Wehkamp et al. 2007). This evidence underlines the importance of this pathway in barrier restitution post-inflammation.

Taken together, this evidence shows that while both Wnt and Notch pathways have known critical roles in immune and gastrointestinal homeostasis, and are also targeted by genetic variation that underlies rare, monogenic forms of IBD, our findings significantly extend evidence in support for these pathways to also be disrupted by common regulatory variants that confer IBD risk. We also show these pathways to be significantly overrepresented in our data (see response to comment 1 by reviewer #4/5), and refine these effects to specific cellular contexts. We have therefore provided the following additional text in the revised manuscript to better present this evidence:

L644: "*The context-specific role of Notch signalling is further exemplified by evidence that Notch-ligand DLL4 induced by TLR stimulation in circulating human myeloid cells may promote effector T cell polarisation associated with graft versus host disease (Meng et al. 2016). Conversely, expression of Notch signalling targets RBP-J, NOTCH1 and HES1 is critical to maintain epithelial barrier function and protect against colitis in mice (Obata et al. 2012; Dunkin et al. 2018).* "

L678: "*Together, these findings motivate the hypothesis that disruption of epithelial renewal and stem cell maintenance in the absorptive epithelial cells of the rectum may be particularly relevant to IBD pathogenesis - consistent with work that highlights Wnt signalling to be reduced in CD patients, independent of inflammation(Wehkamp et al. 2007). Regulators of both Notch and Wnt signalling also harbour rare variants that predispose to monogenic forms of IBD, such as FERMT1, XIAP, AMAD17, IL2RA, ITCH and TGFBR2 (Blaydon et al. 2011; Uhlig et al. 2021; Bolton et al. 2022; Ouahed et al. 2020; Pazmandi et al. 2019; Nambu et al. 2022). In our data, genes in these pathways are notably more frequently nominated as disease effector genes at IBD GWAS loci—more than doubling and tripling the representation of Notch and Wnt pathway members, respectively. This extends the evidence that dysregulation of these pathways contributes to complex IBD risk, and refines it to specific epithelial and myeloid cellular contexts.*

Genes involved in developmental pathways such as Wnt and Notch are pleiotropic and tightly regulated, with well-documented roles in oncogenesis as well as repair…"

And in conjunction with comment 1 by reviewer #4/5:

L385: *"Of the 13 IBD loci where the strongest colocalising regulatory effects were observed in cDCs, two nominate genes involved in Notch signalling. When considered across all disease effector genes, Notch pathway members are significantly enriched (p = 0.026): whereas Open*

Targets Genetics and Cuomo et al. (Cuomo et al. 2025) previously nominated four genes in this pathway, we identify six additional Notch regulators."

L457: *"Previously nominated effector genes by Open Targets Genetics and Cuomo et al., (Cuomo et al. 2025) include only six Wnt regulators, however we nominate an additional fourteen, making this pathway over-represented amongst our disease effector genes (p=0.015)."*

4. The role and interpretation of interaction eQTLs could be clarified. These analyses are potentially very insightful, but were not explained in enough depth. For example, regarding the association with RNF14, the authors suggest that the risk allele reduces RNF14 expression in inflamed enterocytes. However, as I understand it, the measured ileal inflammation is not specific to enterocytes, so this interpretation may be inaccurate. Overall, the biological interpretation of interaction effects should be more precisely described.

We thank the reviewer for their critical evaluation of the presentation of our ieQTL results, and apologise for the lack of clarity. The inflammation status of our patients is derived from macroscopic scoring at the TI by clinicians at the time of sampling, reported previously (Krzak et al. 2024). This inflammation will be caused by a concert of gene expression changes that affect a range of cell-types, including, but not limited to, enterocytes.

We have therefore clarified the derivation of the inflammation score, and potential implication of these findings in the revised manuscript:

L216: "*We performed interaction eQTL (ieQTL) mapping using genotype–expression models that included interaction terms for age, sex and smoking status as well as Crohn's disease diagnosis and macroscopic degree of ileal inflammation at the time of sampling, where possible (Methods). This analysis could uncover effects that are exacerbated by, specific to, or diminished during disease onset or progression.*"

With respect to the specific example of *RNF14*, we have clarified that inflammation is determined at the sample level. The relevant statement in the manuscript now reads:

L493: "*In enterocytes from inflamed samples, the CD risk allele was associated with reduced RNF14 expression (Fig. 5d), suggesting that Wnt signalling and epithelial regeneration may be impaired in the presence of inflammation*"

5. The main text should include more detail on the statistical thresholds used for eQTL discovery. For example, line 117 reports 84,376 eQTLs, but it is unclear what significance cutoff was applied. Providing this information is essential for evaluating the strength of the reported associations. I did not find clarification on this point in the Methods section either. What p-value threshold was used, and how was multiple hypothesis testing addressed?

We appreciate the reviewer's concerns over the clarity of thresholds we apply in order to declare significant eQTL associations. We employed TensorQTL to perform our association testing (L997). This method applies multiple testing correction thresholds that vary slightly for

primary, non-primary, and interaction eQTL effects, as described here https://github.com/broadinstitute/tensorqtl.

We have now included the following detailed description section in the methods (L1026):

"***Multiple hypothesis testing correction***

Correction of p-values to account for multiple hypothesis testing in eQTL mapping was done within each cellular annotation independently. This varied slightly for primary, conditional and interaction effects and is detailed below.

Multiple testing correction of primary signals

1) *Within each gene, multiple variants are being tested for their association with expression. To account for this, expression is permuted 1000 times, and associations with each variant are recalculated. This procedure allows estimation of a null distribution of associations, and calculation of beta-approximated empirical p-values (beta-p). The variant with the smallest beta-p value is then selected for each gene.*

2) *Multiple genes are being tested for eQTL associations within each annotation. A q-value correction(Storey and Tibshirani 2003) is then applied to the variants with the smallest beta-p value per gene, allowing estimation of a genome-wide false discovery rate (FDR). We then apply a threshold of FDR<0.05 to account for both the number of variants being tested per gene and the number of genes, while accommodating for the varied underlying null distributions.*

Multiple testing correction of conditionally independent effects

For genes with a significant eQTL association in a cellular annotation (genome-wide FDR<0.05), subsequent signals are then tested in a forward regression framework by iteratively incorporating variants into the tested model. Firstly, the minimum beta-p value with genome-wide FDR<0.05 is defined across all genes (min-beta-p). During each round of conditional analysis, associations between variants and gene expression level are re-tested and permuted, and the variant with the smallest beta-p value is selected for each gene. If this variant has a beta-p < min-beta-p from the primary analysis, it is declared a conditionally independent signal and included into the model being tested for that gene in the next round. Because the beta-p must always be less than min-beta-p, conditional signals are iteratively mapped per gene until they would fail to pass the genome-wide FDR<0.05 from the primary analysis.

Multiple testing correction of interaction effects

Following ieQTL testing, the number of effectively independent variants being tested is estimated for each gene using eigenMT(Davis et al. 2016). Nominal p-values for ieQTL effects are then adjusted using the Bonferroni method to account for the number of effectively independent tests being performed (emt-p). The variant with the minimum emt-p value is then selected for each gene, and a Benjamini-Hochberg adjustment is then applied to account for

the number of genes being tested (adjusted p). Reported ieQTL effects all have adjusted p < 0.05."

We also adjusted our description of the LD clumping procedure to incorporate this detail (L1070)

"***Aggregation of QTL effects***

Lead variants for the same genetic association may differ across conditions or QTL types (primary, conditional, interaction) due to minor changes in significance. To group lead variants across all tests and define comparable QTL signals, LD clumping was performed using the '--clump' flag in plink v1.90b6.27(Chang et al. 2015) and a threshold of r^2>0.5 for variants within 1Mb with no additional significance filter. This was performed using lead variants from primary eQTLs, conditionally independent eQTLs (genome-wide FDR < 0.05), ieQTLs (adjusted p < 0.05) and lead variants after intersection with GWAS summary statistics to help map colocalisation events to eQTLs".

In order to clarify this detail in the main text of the manuscript, we have adjusted the text in the following positions, incorporating the updated values:

L168 - "*We identified 88,548 eQTLs associated with the expression levels of 20,389 of the 24,761 genes tested (82.2%) (genome-wide false discovery rate [FDR]<0.05, Methods).*"

L220 - *"We identified 1,051 ieQTLs regulating the expression of 977 genes (ieGenes), and 764 (78.2%) of these ieGenes were modulated by gut inflammation status (adjusted p value < 0.05, Methods)"*

Minor

1. In several figure panels, the text is too small and the colors too soft, making them difficult to read. Notable examples include the figure legend text and gene names in Fig. 4 (especially panel b), Fig. 5, and the bottom column labels in Fig. 6. This issue appears in other figures as well and should be addressed for overall readability.

In response to this comment and others, we have adjusted our presentation of colocalisation manhattan plots (Fig. 4b, 5). We have also rotated Fig. 6, increasing axes text and title size, in addition to enlarging that of Fig. 2b, Fig. 2c, Fig. 3, Fig. 4, Fig. 5 and Supplementary Fig. S15, which we believed also suffered from low readability.

2. Fig. 2e: The two values above the violin plot should be clearly labeled with units (e.g., "%" and "MB") to avoid confusion.

Figure 2e (now Figure 2f) has been updated to include the units for these values.

3. Fig. 3a: Although the main text provides context, the numbers outside the pie chart are unclear. Please clarify their meaning in the figure legend or annotate the panel directly.

The legend for Figure 3a now reads:

"*The number of IBD GWAS loci (Liu et al. 2023) coloured by the proportion that previously colocalised (posterior probability of hypothesis 4; PPH4 > 0.75) with existing bulk QTL studies in Open Targets Genetics (see Methods), or do so for the first time with IBDverse sc-eQTLs. The total number of loci (321) is shown on top, along with the cumulative number of loci for which disease effector genes are nominated by each dataset.*"

4. Fig. 3b: The figure legend does not clearly explain what the odds ratio on the y-axis represents. A more detailed description would improve interpretability.

To improve clarity, we have updated the y-axis of Fig 3b to "*Effector gene discovery likelihood (OR)*", and the corresponding legend, which now reads:

"*Comparison between the proportion of eGenes detected (x-axis), and the likelihood that effector genes are discovered at each resolution, expressed as a ratio of odds (OR) between disease effector gene and eGene detection (y-axis). Values >1 indicate an enrichment of disease effector genes amongst eGenes, values < 1 indicate underrepresentation of disease effector genes amongst eGenes.*"

5. Line 600 (Methods): The phrase "quality control and QC" is redundant. Please remove either "quality control" or "QC."

This has now been rectified.

6. The link for the colocalization code was not working: [https://github.com/andersonlab/snakemake_colocalisation [github.com]](https://github.com/andersonlab/snakemake_colocalisation)

We apologise for our error of not making this available upon initial submission. This has now been rectified at the following link: https://github.com/andersonlab/snakemake_colocalisation_sceqtl

<u>Referee #5 (Remarks on code availability):</u>

I did not run the code but I skimmed through it. Most of the code was available. There was one repository that wasn't available: [https://github.com/andersonlab/snakemake_colocalisation [github.com]](https://github.com/andersonlab/snakemake_colocalisation)

References for this response

Atrian, Sílvia, and Mercè Capdevila. 2013. "Metallothionein-Protein Interactions." *Biomolecular Concepts* 4 (2): 143–60.

Blaydon, Diana C., Paolo Biancheri, Wei-Li Di, Vincent Plagnol, Rita M. Cabral, Matthew A. Brooke, David A. van Heel, et al. 2011. "Inflammatory Skin and Bowel Disease Linked to ADAM17 Deletion." *The New England Journal of Medicine* 365 (16): 1502–8.

Bolton, Chrissy, Christopher S. Smillie, Sumeet Pandey, Rasa Elmentaite, Gabrielle Wei, Carmen Argmann, Dominik Aschenbrenner, et al. 2022. "An Integrated Taxonomy for Monogenic Inflammatory Bowel Disease." *Gastroenterology* 162 (3): 859–76.

Bonnet, Fabrice, and André Scheen. 2017. "Understanding and Overcoming Metformin

Gastrointestinal Intolerance." *Diabetes, Obesity & Metabolism* 19 (4): 473–81.
Brown, Christopher D., Lara M. Mangravite, and Barbara E. Engelhardt. 2013. "Integrative Modeling of eQTLs and Cis-Regulatory Elements Suggests Mechanisms Underlying Cell Type Specificity of eQTLs." *PLoS Genetics* 9 (8): e1003649.
Bryois, Julien, Daniela Calini, Will Macnair, Lynette Foo, Eduard Urich, Ward Ortmann, Victor Alejandro Iglesias, et al. 2022. "Cell-Type-Specific Cis-eQTLs in Eight Human Brain Cell Types Identify Novel Risk Genes for Psychiatric and Neurological Disorders." *Nature Neuroscience* 25 (8): 1104–12.
Chang, Christopher C., Carson C. Chow, Laurent Cam Tellier, Shashaank Vattikuti, Shaun M. Purcell, and James J. Lee. 2015. "Second-Generation PLINK: Rising to the Challenge of Larger and Richer Datasets." *GigaScience* 4 (1): 7.
Cuomo, Anna S. E., Giordano Alvari, Christina B. Azodi, single-cell eQTLGen consortium, Davis J. McCarthy, and Marc Jan Bonder. 2021. "Optimizing Expression Quantitative Trait Locus Mapping Workflows for Single-Cell Studies." *Genome Biology* 22 (1): 188.
Cuomo, Anna S. E., Eleanor Spenceley, Hope A. Tanudisastro, Blake Bowen, Albert Henry, Hao Lawrence Huang, Angli Xue, et al. 2025. "Impact of Rare and Common Genetic Variation on Cell Type-Specific Gene Expression." *medRxiv*. https://doi.org/10.1101/2025.03.20.25324352.
Davis, Joe R., Laure Fresard, David A. Knowles, Mauro Pala, Carlos D. Bustamante, Alexis Battle, and Stephen B. Montgomery. 2016. "An Efficient Multiple-Testing Adjustment for eQTL Studies That Accounts for Linkage Disequilibrium between Variants." *The American Journal of Human Genetics* 98 (1): 216–24.
Ding, Ruofan, Qixuan Wang, Lihai Gong, Ting Zhang, Xudong Zou, Kewei Xiong, Qi Liao, Mireya Plass, and Lei Li. 2024. "scQTLbase: An Integrated Human Single-Cell eQTL Database." *Nucleic Acids Research* 52 (D1): D1010–17.
Doody, Rachelle S., Rema Raman, Martin Farlow, Takeshi Iwatsubo, Bruno Vellas, Steven Joffe, Karl Kieburtz, et al. 2013. "A Phase 3 Trial of Semagacestat for Treatment of Alzheimer's Disease." *The New England Journal of Medicine* 369 (4): 341–50.
Dunkin, David, Alina C. Iuga, Sanda Mimouna, Carolyn L. Harris, Jean-Vianney Haure-Mirande, Dominique Bozec, Garabet Yeretssian, and Stephanie Dahan. 2018. "Intestinal Epithelial Notch-1 Protects from Colorectal Mucinous Adenocarcinoma." *Oncotarget* 9 (71): 33536–48.
Emani, Prashant S., Jason J. Liu, Declan Clarke, Matthew Jensen, Jonathan Warrell, Chirag Gupta, Ran Meng, et al. 2024. "Single-Cell Genomics and Regulatory Networks for 388 Human Brains." *Science (New York, N.Y.)* 384 (6698): eadi5199.
eQTLGen Consortium, BIOS Consortium, Eleonora Porcu, Sina Rüeger, Kaido Lepik, Federico A. Santoni, Alexandre Reymond, and Zoltán Kutalik. 12/2019. "Mendelian Randomization Integrating GWAS and eQTL Data Reveals Genetic Determinants of Complex and Clinical Traits." *Nature Communications* 10 (1): 3300.
Erkert, Lena, Reyes Gamez-Belmonte, Melanie Kabisch, Lena Schödel, Jay V. Patankar, Miguel Gonzalez-Acera, Mousumi Mahapatro, et al. 2024. "Alzheimer's Disease-Related Presenilins Are Key to Intestinal Epithelial Cell Function and Gut Immune Homoeostasis." *Gut* 73 (10): 1618–31.
Garrido-Trigo, Alba, Ana M. Corraliza, Marisol Veny, Isabella Dotti, Elisa Melón-Ardanaz, Aina Rill, Helena L. Crowell, et al. 2023. "Macrophage and Neutrophil Heterogeneity at Single-Cell Spatial Resolution in Human Inflammatory Bowel Disease." *Nature Communications* 14 (1): 4506.
Giacconi, Robertina, Catia Cipriano, Elisa Muti, Laura Costarelli, Cardelli Maurizio, Vittorio Saba, Nazzarena Gasparini, Marco Malavolta, and Eugenio Mocchegiani. 2005. "Novel -209A/G MT2A Polymorphism in Old Patients with Type 2 Diabetes and Atherosclerosis: Relationship with Inflammation (IL-6) and Zinc." *Biogerontology* 6 (6): 407–13.
Giambartolomei, Claudia, Damjan Vukcevic, Eric E. Schadt, Lude Franke, Aroon D. Hingorani, Chris Wallace, and Vincent Plagnol. 2014. "Bayesian Test for Colocalisation between Pairs of Genetic Association Studies Using Summary Statistics." *PLoS Genetics* 10 (5): e1004383.

Goncharov, Tatiana, Stefanie Hedayati, Melinda M. Mulvihill, Anita Izrael-Tomasevic, Kerry Zobel, Surinder Jeet, Anna V. Fedorova, et al. 2018. “Disruption of XIAP-RIP2 Association Blocks NOD2-Mediated Inflammatory Signaling.” *Molecular Cell* 69 (4): 551–65.e7.

GTEx Consortium. 2020. “The GTEx Consortium Atlas of Genetic Regulatory Effects across Human Tissues.” *Science (New York, N.Y.)* 369 (6509): 1318–30.

Gutierrez-Arcelus, Maria, Halit Ongen, Tuuli Lappalainen, Stephen B. Montgomery, Alfonso Buil, Alisa Yurovsky, Julien Bryois, et al. 2015. “Tissue-Specific Effects of Genetic and Epigenetic Variation on Gene Regulation and Splicing.” *PLoS Genetics* 11 (1): e1004958.

Hatzikotoulas, Konstantinos, Lorraine Southam, Lilja Stefansdottir, Cindy G. Boer, Merry-Lynn McDonald, J. Patrick Pett, Young-Chan Park, et al. 2025. “Translational Genomics of Osteoarthritis in 1,962,069 Individuals.” *Nature* 641 (8065): 1217–24.

Hu, Madeleine D., Natasha B. Golovchenko, Grace L. Burns, Prema M. Nair, Thomas J. Kelly 4th, Jonathan Agos, Mudar Zand Irani, et al. 2022. “Γδ Intraepithelial Lymphocytes Facilitate Pathological Epithelial Cell Shedding via CD103-Mediated Granzyme Release.” *Gastroenterology* 162 (3): 877–89.e7.

Hur, Ji-Yeun. 2022. “Γ-Secretase in Alzheimer’s Disease.” *Experimental & Molecular Medicine* 54 (4): 433–46.

Inoue, Ken-Ichiro, Hirohisa Takano, Akinori Shimada, Emiko Wada, Rie Yanagisawa, Miho Sakurai, Masahiko Satoh, and Toshikazu Yoshikawa. 2006. “Role of Metallothionein in Coagulatory Disturbance and Systemic Inflammation Induced by Lipopolysaccharide in Mice.” *FASEB Journal: Official Publication of the Federation of American Societies for Experimental Biology* 20 (3): 533–35.

Krzak, Monika, Tobi Alegbe, D. Leland Taylor, Gareth-Rhys Jones, Mennatallah H. Ghouraba, Michelle Strickland, Bradley T. Harris, et al. 2024. “Single-Cell RNA Sequencing of Terminal Ileal Biopsies Identifies Signatures of Crohn’s Disease Pathogenesis.” *medRxiv*. https://doi.org/10.1101/2023.09.06.23295056.

Lammi, Vilma, Tomoko Nakanishi, Samuel E. Jones, Shea J. Andrews, Juha Karjalainen, Beatriz Cortés, Heath E. O’Brien, et al. 2025. “Genome-Wide Association Study of Long COVID.” *Nature Genetics* 57 (6): 1402–17.

Lange, Katrina M. de, Loukas Moutsianas, James C. Lee, Christopher A. Lamb, Yang Luo, Nicholas A. Kennedy, Luke Jostins, et al. 2017. “Genome-Wide Association Study Implicates Immune Activation of Multiple Integrin Genes in Inflammatory Bowel Disease.” *Nature Genetics* 49 (2): 256–61.

Laukens, Debby, Anouk Waeytens, Pieter De Bleser, Claude Cuvelier, and Martine De Vos. 2009. “Human Metallothionein Expression under Normal and Pathological Conditions: Mechanisms of Gene Regulation Based on in Silico Promoter Analysis.” *Critical Reviews in Eukaryotic Gene Expression* 19 (4): 301–17.

Law, Philip J., James Studd, James Smith, Jayaram Vijayakrishnan, Bradley T. Harris, Maria Mandelia, Charlie Mills, Malcolm G. Dunlop, and Richard S. Houlston. 2024. “Systematic Prioritization of Functional Variants and Effector Genes Underlying Colorectal Cancer Risk.” *Nature Genetics* 56 (10): 2104–11.

Lin, Si-Nan, Alessandro Musso, Jie Wang, Pranab K. Mukherjee, Gail A. West, Ren Mao, Ruishen Lyu, et al. 2022. “Human Intestinal Myofibroblasts Deposited Collagen VI Enhances Adhesiveness for T Cells - A Novel Mechanism for Maintenance of Intestinal Inflammation.” *Matrix Biology: Journal of the International Society for Matrix Biology* 113 (November):1–21.

Meng, Lijun, Zhenjiang Bai, Shan He, Kazuhiro Mochizuki, Yongnian Liu, Janaki Purushe, Hongxing Sun, et al. 2016. “The Notch Ligand DLL4 Defines a Capability of Human Dendritic Cells in Regulating Th1 and Th17 Differentiation.” *The Journal of Immunology* 196 (3): 1070–80.

Meng, Xiangrui, Georgina Navoly, Olga Giannakopoulou, Daniel F. Levey, Dora Koller, Gita A. Pathak, Nastassja Koen, et al. 2024. “Multi-Ancestry Genome-Wide Association Study of Major Depression Aids Locus Discovery, Fine Mapping, Gene Prioritization and

Causal Inference." *Nature Genetics* 56 (2): 222–33.
Minikel, Eric Vallabh, Jeffery L. Painter, Coco Chengliang Dong, and Matthew R. Nelson. 2024. "Refining the Impact of Genetic Evidence on Clinical Success." *Nature* 629 (8012): 624–29.
Mostafavi, Hakhamanesh, Jeffrey P. Spence, Sahin Naqvi, and Jonathan K. Pritchard. 2023. "Systematic Differences in Discovery of Genetic Effects on Gene Expression and Complex Traits." *Nature Genetics* 55 (11): 1866–75.
Murphy, Alan E., and Nathan G. Skene. 2022. "A Balanced Measure Shows Superior Performance of Pseudobulk Methods in Single-Cell RNA-Sequencing Analysis." *Nature Communications* 13 (1): 7851.
Nambu, Ryusuke, Neil Warner, Daniel J. Mulder, Daniel Kotlarz, Dermot P. B. McGovern, Judy Cho, Christoph Klein, et al. 2022. "A Systematic Review of Monogenic Inflammatory Bowel Disease." *Clinical Gastroenterology and Hepatology: The Official Clinical Practice Journal of the American Gastroenterological Association* 20 (4): e653–63.
Nathan, Aparna, Samira Asgari, Kazuyoshi Ishigaki, Cristian Valencia, Tiffany Amariuta, Yang Luo, Jessica I. Beynor, et al. 2022. "Single-Cell eQTL Models Reveal Dynamic T Cell State Dependence of Disease Loci." *Nature* 606 (7912): 120–28.
Natri, Heini M., Christina B. Del Azodi, Lance Peter, Chase J. Taylor, Sagrika Chugh, Robert Kendle, Mei-I Chung, et al. 2024. "Cell-Type-Specific and Disease-Associated Expression Quantitative Trait Loci in the Human Lung." *Nature Genetics* 56 (4): 595–604.
Nelson, Matthew R., Hannah Tipney, Jeffery L. Painter, Judong Shen, Paola Nicoletti, Yufeng Shen, Aris Floratos, et al. 2015. "The Support of Human Genetic Evidence for Approved Drug Indications." *Nature Genetics* 47 (8): 856–60.
Obata, Yuuki, Daisuke Takahashi, Masashi Ebisawa, Kisa Kakiguchi, Shigenobu Yonemura, Toshi Jinnohara, Takashi Kanaya, et al. 2012. "Epithelial Cell-Intrinsic Notch Signaling Plays an Essential Role in the Maintenance of Gut Immune Homeostasis." *The Journal of Immunology* 188 (5): 2427–36.
Oelen, Roy, Dylan H. de Vries, Harm Brugge, M. Grace Gordon, Martijn Vochteloo, single-cell eQTLGen consortium, BIOS Consortium, et al. 2022. "Single-Cell RNA-Sequencing of Peripheral Blood Mononuclear Cells Reveals Widespread, Context-Specific Gene Expression Regulation upon Pathogenic Exposure." *Nature Communications* 13 (1): 3267.
Okuno, T., A. Andoh, S. Bamba, Y. Araki, Y. Fujiyama, M. Fujiyama, and T. Bamba. 2002. "Interleukin-1beta and Tumor Necrosis Factor-Alpha Induce Chemokine and Matrix Metalloproteinase Gene Expression in Human Colonic Subepithelial Myofibroblasts." *Scandinavian Journal of Gastroenterology* 37 (3): 317–24.
Oliva, Meritxell, Manuel Muñoz-Aguirre, Sarah Kim-Hellmuth, Valentin Wucher, Ariel D. H. Gewirtz, Daniel J. Cotter, Princy Parsana, et al. 2020. "The Impact of Sex on Gene Expression across Human Tissues." *Science (New York, N.Y.)* 369 (6509). https://doi.org/10.1126/science.aba3066.
Ouahed, Jodie, Elizabeth Spencer, Daniel Kotlarz, Dror S. Shouval, Matthew Kowalik, Kaiyue Peng, Michael Field, et al. 2020. "Very Early Onset Inflammatory Bowel Disease: A Clinical Approach with a Focus on the Role of Genetics and Underlying Immune Deficiencies." *Inflammatory Bowel Diseases* 26 (6): 820–42.
Pazmandi, Julia, Artem Kalinichenko, Rico Chandra Ardy, and Kaan Boztug. 2019. "Early-Onset Inflammatory Bowel Disease as a Model Disease to Identify Key Regulators of Immune Homeostasis Mechanisms." *Immunological Reviews* 287 (1): 162–85.
Pullin, Jeffrey M., and Chris Wallace. 2025. "Flexible and Efficient Count-Distribution and Mixed-Model Methods for eQTL Mapping with Quasar." *medRxiv*. https://doi.org/10.1101/2025.07.17.25331702.
Razuvayevskaya, Olesya, Irene Lopez, Ian Dunham, and David Ochoa. 2024. "Genetic Factors Associated with Reasons for Clinical Trial Stoppage." *Nature Genetics* 56 (9): 1862–67.

Sirvent, Sofia, Andres F. Vallejo, Emma Corden, Ying Teo, James Davies, Kalum Clayton, Eleanor G. Seaby, et al. 2023. “Impaired Expression of Metallothioneins Contributes to Allergen-Induced Inflammation in Patients with Atopic Dermatitis.” *Nature Communications* 14 (1): 2880.

Soskic, Blagoje, Eddie Cano-Gamez, Deborah J. Smyth, Kirsty Ambridge, Ziying Ke, Julie C. Matte, Lara Bossini-Castillo, et al. 2022. “Immune Disease Risk Variants Regulate Gene Expression Dynamics during CD4+ T Cell Activation.” *Nature Genetics* 54 (6): 817–26.

Stankey, C. T., C. Bourges, L. M. Haag, T. Turner-Stokes, A. P. Piedade, C. Palmer-Jones, I. Papa, et al. 2024. “A Disease-Associated Gene Desert Directs Macrophage Inflammation through ETS2.” *Nature* 630 (8016): 447–56.

Storey, John D., Andrew J. Bass, Alan Dabney, and David Robinson. 2023. “Qvalue: Q-Value Estimation for False Discovery Rate Control.” https://doi.org/10.18129/B9.bioc.qvalue.

Storey, John D., and Robert Tibshirani. 2003. “Statistical Significance for Genomewide Studies.” *Proceedings of the National Academy of Sciences of the United States of America* 100 (16): 9440–45.

Subramaniam, Karthik, Manoj P. Joseph, and Lakshmi A. Babu. 2021. “A Common Drug Causing a Common Side Effect at an Uncommon Time: Metformin-Induced Chronic Diarrhea and Weight Loss after Years of Treatment.” *Clinical Diabetes: A Publication of the American Diabetes Association* 39 (2): 237–40.

Takano, H., K. Inoue, R. Yanagisawa, M. Sato, A. Shimada, T. Morita, M. Sawada, K. Nakamura, C. Sanbongi, and T. Yoshikawa. 2004. “Protective Role of Metallothionein in Acute Lung Injury Induced by Bacterial Endotoxin.” *Thorax* 59 (12): 1057–62.

Tian, Yijun, Lang Wu, Chang-Ching Huang, and Liang Wang. 2024. “Identify Regulatory eQTLs by Multiome Sequencing in Prostate Single Cells.” *bioRxivorg*. https://doi.org/10.1101/2024.06.19.599704.

Trajanoska, Katerina, Claude Bhérer, Daniel Taliun, Sirui Zhou, J. Brent Richards, and Vincent Mooser. 2023. “From Target Discovery to Clinical Drug Development with Human Genetics.” *Nature* 620 (7975): 737–45.

Tsuji, Toshifumi, Yuji Naito, Tomohisa Takagi, Munehiro Kugai, Hiroyuki Yoriki, Ryusuke Horie, Akifumi Fukui, et al. 2013. “Role of Metallothionein in Murine Experimental Colitis.” *International Journal of Molecular Medicine* 31 (5): 1037–46.

Uhlig, Holm H., Fabienne Charbit-Henrion, Daniel Kotlarz, Dror S. Shouval, Tobias Schwerd, Caterina Strisciuglio, Lissy de Ridder, et al. 2021. “Clinical Genomics for the Diagnosis of Monogenic Forms of Inflammatory Bowel Disease: A Position Paper from the Paediatric IBD Porto Group of European Society of Paediatric Gastroenterology, Hepatology and Nutrition.” *Journal of Pediatric Gastroenterology and Nutrition* 72 (3): 456–73.

Uhlig, Holm H., Tobias Schwerd, Sibylle Koletzko, Neil Shah, Jochen Kammermeier, Abdul Elkadri, Jodie Ouahed, et al. 2014. “The Diagnostic Approach to Monogenic Very Early Onset Inflammatory Bowel Disease.” *Gastroenterology* 147 (5): 990–1007.e3.

Uo, Michihide, Tadakazu Hisamatsu, Jun Miyoshi, Daiki Kaito, Kazuaki Yoneno, Mina T. Kitazume, Maiko Mori, et al. 2013. “Mucosal CXCR4+ IgG Plasma Cells Contribute to the Pathogenesis of Human Ulcerative Colitis through FcγR-Mediated CD14 Macrophage Activation.” *Gut* 62 (12): 1734–44.

Wallace, Chris. 2021. “A More Accurate Method for Colocalisation Analysis Allowing for Multiple Causal Variants.” *PLoS Genetics* 17 (9): e1009440.

Wang, Lida, Havell Markus, Dieyi Chen, Siyuan Chen, Fan Zhang, Shuang Gao, Chachrit Khunsriraksakul, et al. 2025. “An Atlas of Single-Cell eQTLs Dissects Autoimmune Disease Genes and Identifies Novel Drug Classes for Treatment.” *Cell Genomics* 5 (4): 100820.

Wehkamp, Jan, Guoxing Wang, Irmgard Kübler, Sabine Nuding, Alex Gregorieff, Anke Schnabel, Robert J. Kays, et al. 2007. “The Paneth Cell Alpha-Defensin Deficiency of Ileal Crohn’s Disease Is Linked to Wnt/Tcf-4.” *The Journal of Immunology* 179 (5): 3109–18.

Wu, Tianzhi, Erqiang Hu, Shuangbin Xu, Meijun Chen, Pingfan Guo, Zehan Dai, Tingze Feng, et al. 2021. “clusterProfiler 4.0: A Universal Enrichment Tool for Interpreting Omics Data.” *Innovation (Cambridge (Mass.))* 2 (3): 100141.

Yazar, Seyhan, Jose Alquicira-Hernandez, Kristof Wing, Anne Senabouth, M. Grace Gordon, Stacey Andersen, Qinyi Lu, et al. 2022. “Single-Cell eQTL Mapping Identifies Cell Type-Specific Genetic Control of Autoimmune Disease.” *Science (New York, N.Y.)* 376 (6589): eabf3041.

Zhang, Feng, Shuijiao Guo, Wenhui Zhong, Kaijun Huang, and Yubin Liu. 2021. “Integrative Analysis of Metallothioneins Identifies MT1H as Candidate Prognostic Biomarker in Hepatocellular Carcinoma.” *Frontiers in Molecular Biosciences* 8 (October):672416.

Referee #1 (Remarks to the Author):

The authors have done an outstanding job addressing the concerns of the reviewers and have clarified most of the concerns the reviewers had. Appreciated the length and depth they went to explain things in their rebuttal letter. Still think the study leaves open many unanswered mechanistic questions that need experimental validation, but the authors have argued this is beyond the scope of the study. Major concern with this is that there are already over 200 loci that have been identified for IBD, and except for a few, we still don't know the mechanisms or how they might contribute to disease- thus we have been left with many associations and suggestions on their impact. Nonetheless, the bioanalytical methodology they presented is rigorous and well done, and agree that resolving the eQTLs at higher resolution is a major step forward in functional genomics.

We thank Reviewer 1 for their comments about our previous response, and appreciate the positive remarks regarding the detail we provided and our methodology. We wholeheartedly agree that further experimentation is required to fully characterise the mechanisms by which our nominated genes influence associations confer IBD susceptibility, and sincerely hope researchers are both inspired and empowered to undertake this work - building upon the mechanistic hypotheses we have generated.

Referee #2 (Remarks to the Author):

I co-reviewed this manuscript with one of the reviewers who provided the listed reports.

Referee #3 (Remarks to the Author):

We appreciate the authors' response to our comments and additional analyses which clarified the novelty of this study. We are satisfied with most of the points that were raised in the first review round, but have several remaining points that we think need to be addressed for the publication of the manuscript, which we have summarized below.

In some of their present comments, reviewers #2/3 have asked for more specific comparisons of the disease effector genes we nominate at IBD loci compared with Cuomo *et al* (2025) (comment #1) and Open Targets (comment #4), suggesting we provide some explanation for differences between our study with these other datasets. It is of course of great interest and importance for us to critically evaluate the novelty of our findings, and to this end, we made use of the most comprehensive database of colocalisations between IBD GWAS and molecular QTLs (pQTLs, sQTLs, eQTLs), provided by Open Targets Genetics, and in response to the first round of reviewer comments, have supplemented this with the largest sc-eQTL study ever conducted,

comprising 1,925 blood scRNAseq samples from Cuomo *et al.,* (2025). Comparing against either dataset, we have shown our study nominates hundreds of novel disease effector genes across dozens of loci where no colocalisation has previously been detected.

Although understanding why we may not nominate the same disease effector genes as other studies is an interesting question, discrepancies can arise for both biological and technical reasons. Potential sources of discrepancy include; differences in study size and statistical power; tissue and cellular context; sample condition (e.g. diseased vs. healthy, stimulated vs. unstimulated); analytical methodology (such as fine-mapping strategies or colocalisation approaches); cell/sample annotation; sample preparation methods; analytical parameter choices (e.g. gene expression thresholds); and underlying technology (assay type, sequencing platform, bulk versus single-cell data).

Although we have shown that certain biological factors likely contribute to our improved detection of disease effector genes (e.g. higher eQTL mapping resolution, Fig. 3b, and the use of samples from diseased patients, Fig. 3c), the many technical variables involved make it difficult to explain differences at individual loci. As such, absence of evidence should not be interpreted as evidence of absence. In addition, we are reluctant to open a discussion into why some findings from other studies might not replicate in our dataset as we do not wish to suggest that a lack of replication might indicate that these associations are false.

While we have attempted to quantify the factors that contribute to some of the differences between studies in response to the reviewer's comments (see responses below), for the reasons given above we have not given specific examples at individual loci. Nonetheless, we hope we have still been able to satisfactorily address the reviewer's questions.

Major:

1. **(Previous comment #2): It was great to see that the authors were able to nominate effector genes through colocalization with their eQTL that had not been identified by Cuomo et al. However, we thought that it would be also important to mention what percentage of colocalizations by Cuomo et al. were not identified in the authors' study using colonic tissues, for a fair comparison. For the genes that were only identified by their study, it would be of interest to assess what contributed to the differences between this work and Cuomo et al. For example, whether eQTL from the cell types specific to the colonic immune cells / colonic tissues were more likely to have contributed to the additional discovery, since this study is statistically less powered than Cuomo et al.?**

For clarity, we have chosen to respond to the multiple points in this comment separately.

Firstly, in response to: "**We thought that it would be also important to mention what percentage of colocalizations by Cuomo et al. were not identified in the authors' study using colonic tissues, for a fair comparison**" - After requantifying the overlap of disease effector genes nominated by the two studies, we note 251 disease effector genes detected in Cuomo *et al.,* (PPH4 > 0.8, IBD only), for which we corroborate 82 (32.7%), leaving 169 (67.3%) to be specific to their study. If we focus on eQTLs detected in the intestinal samples of our dataset (ignoring all blood and 'cross-tissue' analyses), we corroborate 67 (26.7%) of the disease effector genes identified by Cuomo *et al*. We now reference these figures in the revised manuscript:

L204: "*Even compared with a sc-eQTL study of peripheral blood mononuclear cells from 1,925 healthy individuals[35], our study nominates 173 novel effector genes for IBD, including 88 across 49 loci where one is not otherwise nominated (PP_{h4}>0.8) - leaving 169 (67.3%) of the 251 effector genes Cuomo et al., nominate to be specific to their dataset.*"

As the reviewer outlines, the substantially larger sample size of the dataset used in Cuomo *et al.,* (1,925 individuals compared with our 421) makes it inherently difficult to make a 'fair comparison' between the two studies. However, using the same threshold for declaring colocalisation, we actually nominate 255 disease effector genes, slightly more than Cuomo *et al.,* with 173 specific to our dataset (as per our response to the previous comment, and L208 in the revised manuscript). We believe this is particularly noteworthy given the disparate sample sizes, and provide further exploration of the novel findings below.

Next, regarding: "**For the genes that were only identified by their study, it would be of interest to assess what contributed to the differences between this work and Cuomo et al. For example, whether eQTL from the cell types specific to the colonic immune cells / colonic tissues were more likely to have contributed to the additional discovery, since this study is statistically less powered than Cuomo et al.**" - To address this comment we quantified, for each major population, the odds ratio between disease effector gene discovery in that population and the discovery of disease effector genes that were not identified in Cuomo *et al.,* analogous to Figure 3c made in response to a previous comment by these reviewers. We then compared this odds ratio, a proxy for the novelty provided by each major population (y-axis), against the proportion of cells from that population that are collected from the blood (x-axis), shown below. It is worth noting this is an imperfect proxy for enrichment of populations in gastrointestinal tissues, given the differences in the number of cells collected from CD and healthy individuals, the varied number of samples from each tissue, and the varied proportion of CD and healthy samples collected from each tissue.

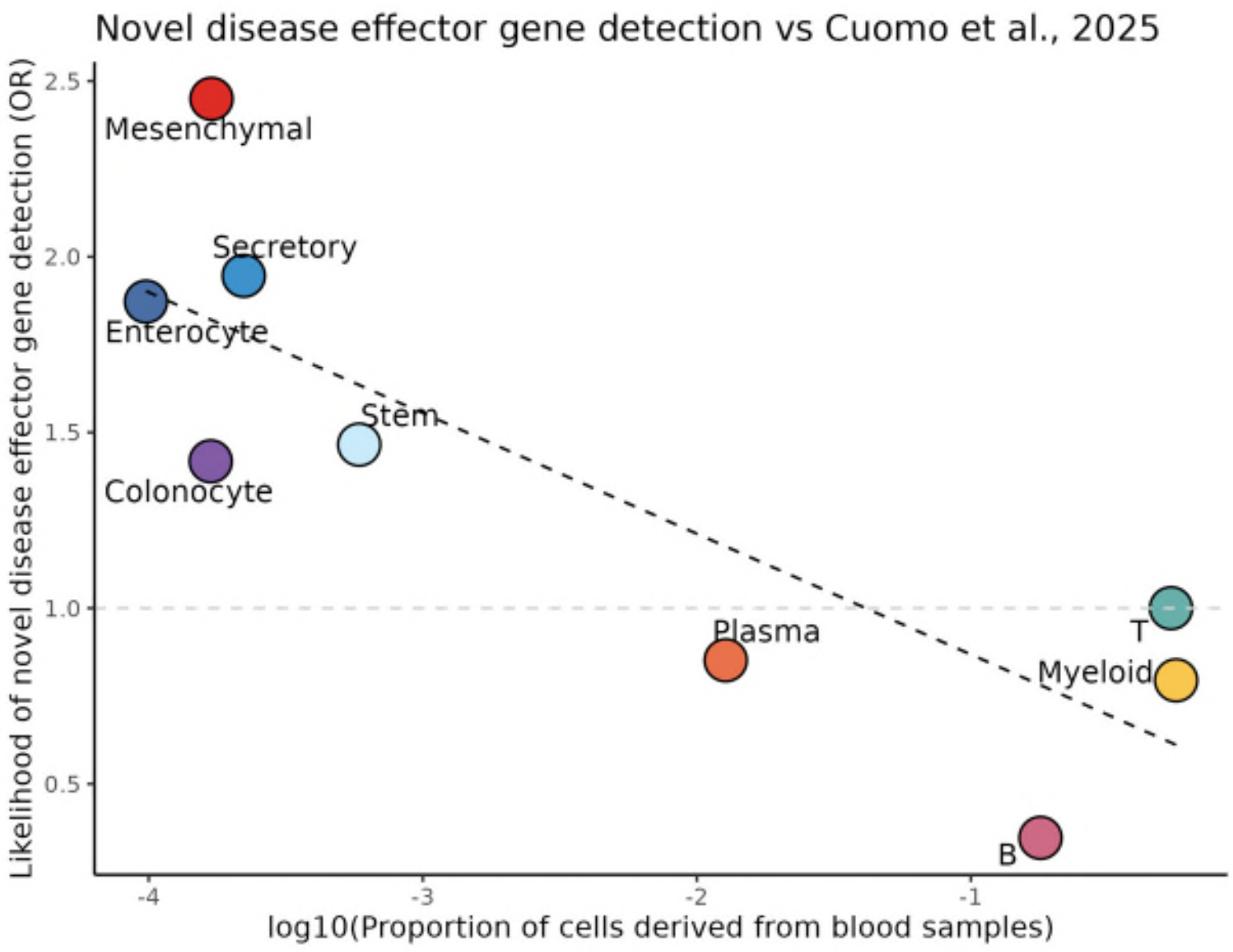

This indicates that in major populations which have the fewest cells present in the blood, such as Mesenchymal (0.017%) and Enterocytes (9.8e-3%), we are more likely to discover a disease effector gene not also found in Cuomo *et al.,* (OR=2.45 and 1.87 respectively). Conversely, Myeloid and T cells, which are the only two populations where the majority of cells are derived from the blood (56.1 and 53.7% respectively), show negative or no enrichment for novel disease effector genes (OR=0.79 and 1.00 respectively). These covariates are statistically associated (slope = -0.344, p = 5.4e-3), highlighting the discovery of novel disease effector genes in our dataset to be negatively associated with the proportion of cells that are derived from the blood in each of our cell-types.

We believe our analysis in response to this comment more formally quantifies the benefit that mapping eQTLs in the primary site of disease provides for identifying disease effector genes. We have therefore included this figure in the Supplementary Materials of the revised manuscript (Supplementary Figure S7), and have updated the text to reference this analysis:

L209: *"The likelihood that we identify a novel disease effector gene compared with this dataset was negatively associated with the number of cells within each major population that was derived from the blood (slope = -0.344, p = 5.4e-3) (Supplementary Fig S7) - indicating a considerable benefit to the inclusion of samples from the primary site of disease."*

2. **(Previous comment #5): Thanks for the clarification on the methods. We appreciate that the authors conducted a simulation study of the power to**

detect colocalization. However, we are still unsure if the colocalization using the interaction term is valid or not. GT:I term can become significant not only in the cases when the true causal variant of GWAS causes gene expression changes in a subset of cells but also in the cases when the variant has significantly heterogenous effect sizes without any apparent subset of cells which drive the association of both expression and GWAS. The latter may not serve as a causal mechanism of GWAS, but rather be a coincidence of a variant with heterogenous effect sizes with expression and a GWAS variant. In the real examples of colocalization between GT:I and GWAS, can the authors show that there exist cell states that demonstrate a causal variant with a significant main effect (G) for the gene expression and also with high PIP with GWAS?

Before addressing this comment directly, we would like to outline that we recognise we are among the first groups to utilise colocalisation analysis to investigate the shared association of variants with disease susceptibility and ieQTLs. It is for this reason that we worked closely with the corresponding author and active developer of the coloc method, Chris Wallace (co-author on this manuscript), to sense-check and validate our approach. To this end, Chris had performed the simulation tests in response to major comment 5 by these reviewers in the previous round of review, which indicated that using ieQTL summary data for colocalisation is unlikely to result in false positive associations being reported.

To address the reviewer's present question - "**can the authors show that there exist cell states that demonstrate a causal variant with a significant main effect (G) for the gene expression and also with high PIP with GWAS?**", we first performed fine-mapping of the GWAS association for CD using SuSIE, identifying a single 95% credible set (CS) of variants (n=53). As the original ieQTL was detected by interaction of genotype with inflammation status in Enterocyte top villus cells, we then tested for main (G) eQTL effects of these variants with *RNF14* expression level in this population, testing within inflamed samples (SES-CD score >= 3, n=58) and uninflamed samples (SES-CD < 3, n=262) separately.

In inflamed samples, the variant from the CD CS with the largest PIP, chr5:142146492:C:A, was the variant with the smallest nominal p-value for the eQTL, with the negative effect on gene-expression remaining significant after FDR correction across these 53 variants (CD GWAS PIP=0.033, β-eQTL=-0.556, eQTL nominal p=1.56e-3, eQTL FDR=1.70e-03). In contrast, in uninflamed samples, this variant was associated with a marginally significant positive effect on gene expression (β-eQTL=0.164, eQTL nominal p=3.22e-2). The regulatory association of this variant on *RNF14* expression in Enterocyte top villus cells from inflamed and non-inflamed individuals is shown below (a).

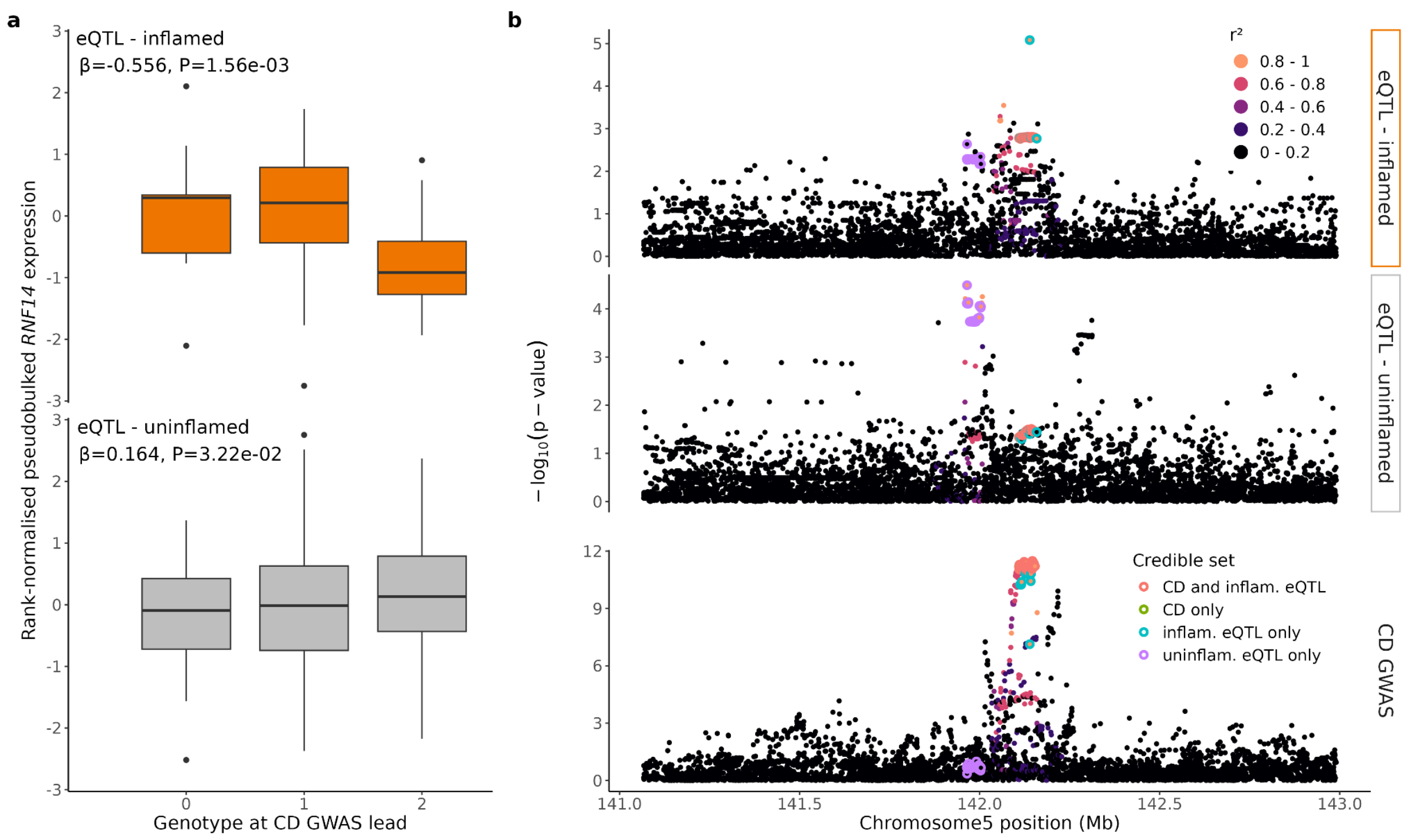

To better characterise the *RNF14* eQTL signals in these subsets, we then performed conventional cis-eQTL mapping, testing for a main effect (G) of all variants within 1Mb of the transcription start site of *RNF14* (MAF>0.05), and subjected these to fine-mapping. Unfortunately, a 95% credible set of variants could not be identified for either eQTL effect using SuSIE, likely due to the reduction in statistical power by focusing on a smaller number of samples. In light of this, we then computed an approximate credible set of variants for the eQTL associations using the Probabilistic Identification of Causal SNPs (PICS) algorithm (Farh *et al.,* 2014, *Nature*), which incorporates the haplotype structure to estimate the posterior probability that variants are within the credible set based on the in-sample LD with the lead variant.

PICS fine mapping identified a non-overlapping set of 64 and 68 variants for eQTLs from inflamed and uninflamed samples respectively. 52 of the 53 CD CS variants were included in the approximate credible set from the inflamed eQTL, but none were included in the eQTL from uninflamed samples. While we acknowledge these approximate credible sets are not as statistically robust as those computed with SuSIE, the substantial overlap of the CD CS with the eQTL CS from inflamed samples is consistent with the regulatory signal being shared with that of CD susceptibility. In addition, the exclusivity of these variants further supports this regulatory effect to be specific to inflamed samples only, in line with our findings from the original ieQTL. The regional association of variants for both eQTLs and CD is shown above (b), with points coloured by their LD with the lead variant from each dataset and outlined by their overlap across datasets.

We believe the evidence presented in response to this comment further supports our original findings, and so have chosen to include this analysis in the revised manuscript, along with additional details in the Methods:

L380: "*Fine-mapping of the CD association at this region identified putatively causal variants which showed main eQTL effects for RNF14 in Enterocytes from inflamed samples (β=-0.556, p=1.56e-3) that was stronger and in opposite direction to that observed in uninflamed samples (β=0.164, p=3.22e-2). The approximate credible set of variants for the eQTL association in inflamed samples included 52 of 53 variants in the credible set for CD GWAS, while none were included in the eQTL from the uninflamed samples (Methods). This supports the genetic association for CD susceptibility in the region to be shared with a regulatory effect on RNF14 expression in Enterocytes that is specific to inflamed samples only (Supplementary Figure S13).*"

L1058: "*Fine-mapping eQTL and CD associations at the RNF14 locus*

To identify variants which are putatively causal at the RNF14 locus, the CD association was fine-mapped using default parameters of coloc-susie(Wallace 2021) and linkage disequilibrium estimates from our cohort. This identified a set of 53 variants with a 95% probability of containing a causal variant (referred to as the '95% credible set'). To assess the likelihood that these variants conferred a regulatory effect within the inflamed and uninflamed samples, we tested these variants for non-interaction univariate eQTL effects in Enterocyte top villus cells for the 58 individuals with either 'mild' or 'severe' inflammation separately to the 262 uninflamed individuals. To identify putatively causal variants for the eQTL associations, we tested all variants within 1Mb of the TSS of RNF14, and applied the Probabilistic Identification of Causal SNPs (PICS) algorithm(Farh et al. 2015) using linkage disequilibrium estimates derived from our cohort, as we did not have statistical power to fine-map using coloc-susie. 52 of the 53 variants included in the 95% credible set for the CD susceptibility association signal were included in the approximate eQTL credible set of inflamed individuals, consistent with both these phenotypes sharing a genetic association in the region. None of the CD CS variants were included in the approximate credible set for the eQTL in uninflamed samples."

If the reviewers still feel dissatisfied with our response, we are willing to move these results to the supplementary and reduce the text dedicated to these findings. While we feel this would be overly cautious and detract from the paper, we do not wish to jeopardise potential acceptance over these results.

3. **(Previous comment #8): The authors explain in their rebuttal that fine-mapping of their eQTLs would not be appropriate given the vast differences in statistical power across cell types represented in their dataset, and instead attempted to address this comment with an LD clumping strategy. While LD clumping is useful for approximating independent signals, it does not facilitate**

identification of the likely causal variant and importantly, it cannot correct for LD-induced sign flips. We would also like to argue that by performing colocalization analyses in other sections, the authors have implicitly already performed fine-mapping analysis. If myofibroblasts do not share exactly the same causal variant as rectal cDC2 and T cells, but instead have causal variants that are in LD, the eQTL effect truly represents a mixture of these signals, which is particularly important when the effect is marginal, as is the case for the "chr1:77461459:A:G" clump index the authors demonstrate. Therefore, we believe that fine-mapping even with caveats is necessary to support the claim of opposite directional effects between cell types, which would represent a very interesting finding of this study worth highlighting.

Firstly, with respect to the comment "**While LD clumping is useful for approximating independent signals, it does not facilitate identification of the likely causal variant**" - We agree that LD clumping is a useful, but imperfect method to determine independent signals across our cell types. Using this method, our goal was to generalise eQTL associations across conditions that may represent the same signal but have slight differences in leading variants. While this approach has been used in this setting previously (Mostafavi *et al.,* 2023, *Nat. Genetics*), we acknowledge that fine-mapping would also be useful for this task, and advantageous to delineate whether eQTL signals indeed share putative causal variants with opposite directions of effect, albeit limited by the requirement for substantial statistical power.

While the reviewer's query is specifically regarding evidence in support of our claim of opposite directions for eQTL effects on *FUBP1*, we sought to first test the likelihood that opposite directions of effect could be identified for disease effector genes across cell-types more generally. To this end, we fine-mapped eQTL associations for the 370 disease effector genes we nominate where the colocalising eQTL variant shows inconsistent direction of effect across conditions (cell-type and tissue pairs), including *FUBP1*, and tested for a maximum of 10 independent signals while using in-sample LD. We then merged credible sets across cell-types where there was i) at least one overlapping variant in the 95% credible sets (CS) and ii) colocalisation of causal variants, calculated using coloc.susie (PP.H4 > 0.75). It is worth noting that to maximise discovery, we attempted fine mapping in all cell-types, without filtering the eQTL effects for statistical significance. Code is available at https://github.com/andersonlab/sceqtl_finemapping.

Although rare, 5 (2.1%) eQTL credible sets containing the disease-associated variant colocalised across multiple cell types but showed opposing directions of effect on gene expression level. One example is the credible set of variants for an eQTL that regulates *IL18R1* expression, which colocalises with CD (PP.H4=0.91). *IL18R1* encodes a receptor for the cytokine IL18, and is the direct target of IL18 receptor agonists such as Iboctadekin that have been trialled to treat lymphoma and melanoma. The lead colocalising eQTL variant from this association, chr2:102390760:T:C, shows opposing

directions of effect in ILCs Nkp44 (Cross-site) (beta=0.53, p=2.64e-13) and NK2 cells from the TI (beta=-0.544, p=1.39e-07), shown below, left. While the number of variants included in the colocalising credible set from these conditions is large (84 variants in each), they colocalise with one another (PP.H4=0.86), suggesting they represent the same genetic signal. The posterior inclusion probability (PIP) of variants in the credible set containing the colocalising variant in each condition is shown below, right, with the colocalising QTL variant shown in orange. Taken together, we believe this indicates that although rare, it is possible for disease-colocating eQTLs to show opposing directions of effect across different cell-types - even within the same major population (in this case, T cells). Notably, the rate of opposing directions of eQTL effects we observe for these disease effector genes is similar to that of eQTLs identified from immune cell-types by recent work (5%, Kanai *et al.,* 2025, *medRxiv*).

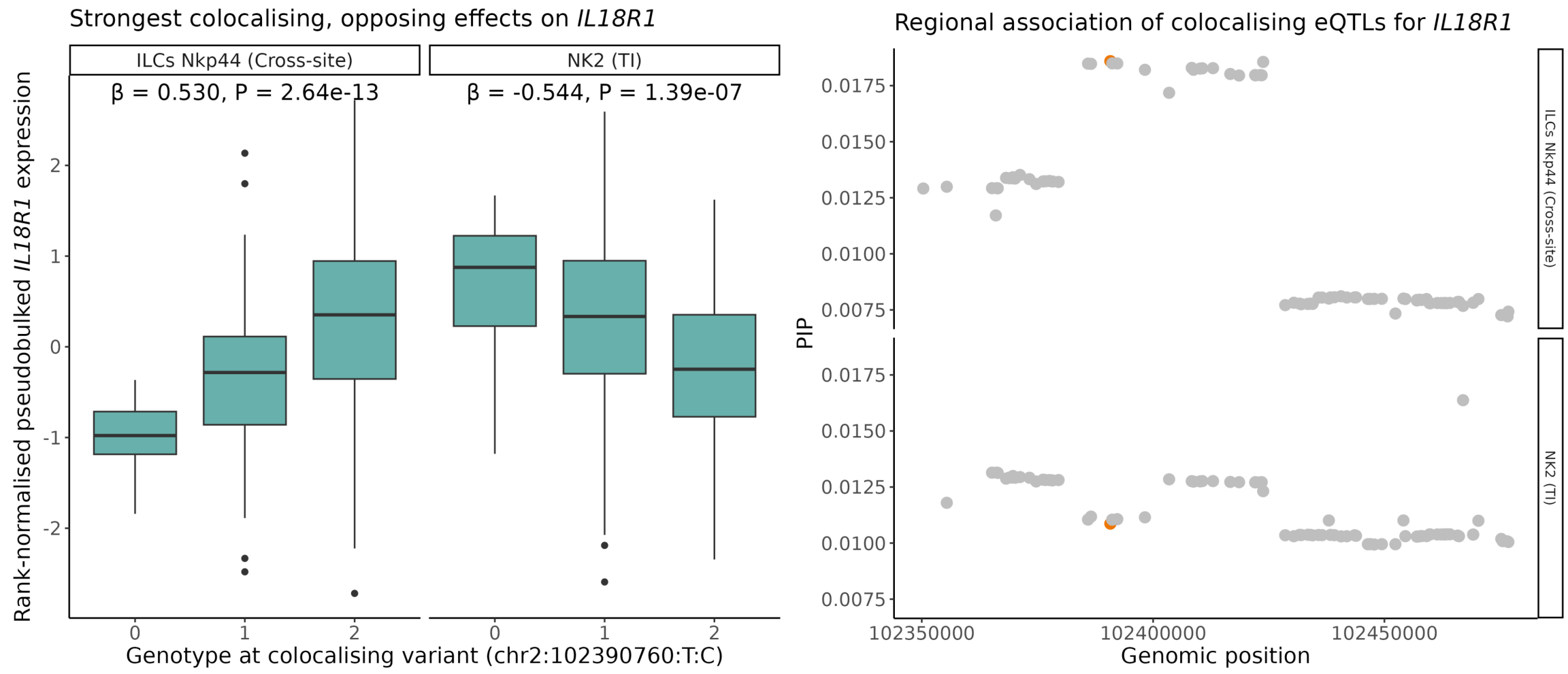

Regarding *FUBP1*, unfortunately, eQTL associations could not be fine-mapped in the three cell-types in question. This is likely due to the limited statistical power, representing the precise scenario we sought to overcome by testing for the proportion of variance explained by the colocalising variant. Overall, eQTLs regulating *FUBP1* could be fine-mapped in 11 conditions, and after merging credible sets that colocalised across conditions (PP.H4>0.75 in all pairwise comparisons), this includes a total of 15 credible sets, including one that contained the colocalising eQTL variant, chr1:77984833:C:A. In the 7 conditions where the disease-colocalising variant was included in a credible set, dosage of the 'A' allele was always associated with a reduction in gene expression (minimum beta=-0.41, maximum beta=-0.31).

In lieu of the statistical power to fine-map eQTLs regulating *FUBP1* in the three cell-types highlighted in the manuscript, we checked the direction of eQTL effects for variants from non-colocalising CS, and of the colocalising variant after conditioning on these other variants. Results from the univariate test of the colocalising variant are shown below in orange ('univariate-coloc'), univariate test of variants from other credible sets in blue ('univariate-other') and conditional tests of the colocalising variant

on variants from non-colocalising CS in grey ('coloc-conditioned'). The horizontal grey line indicates a nominal p-value of 0.05.

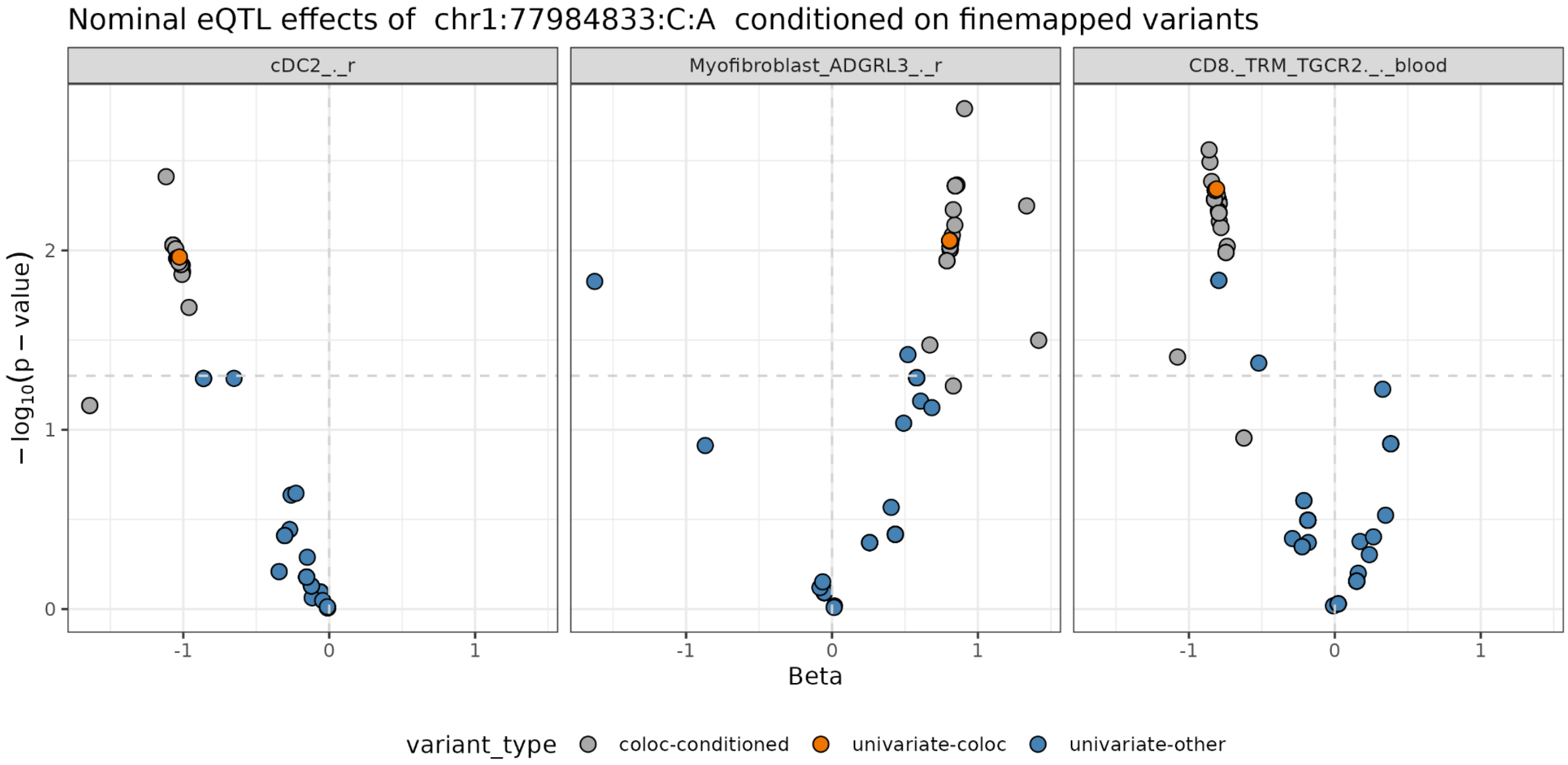

In cDC2 cells from the rectum, all tests have directions of effect that are concordant with the colocalising variant, and only the highlighted variant shows a nominal association with *FUBP1* expression (p<0.05) - hence it is unlikely that any variants from these other credible sets could confound our observation. In the other cell-types, there are variants with opposing direction of effect to the colocalising variant, with one nominally significant opposing association in Myofibroblasts from the rectum (chr1:78468881:A:G). While this could suggest that there is a distinct eQTL influencing the opposite effects we see in Myofibroblasts, conditioning the colocalising eQTL variant on chr1:78468881:A:G still results in a positive direction of effect that is nominally significant (beta=0.91, conditional p-value=1.62e-3). Similarly, in CD8 TRM TGRC2 cells from the blood, there is one nominally significant association that has the opposite direction of effect to the colocalising variant (chr1:78797345:G:A), but conditioning the colocalising QTL on this variant still results in a negative direction of effect that is nominally significant (beta=-0.74, conditional p-value=9.49e-3). Together, this evidence suggests the other putatively causal variants identified in our analysis are unlikely to confound our observation of opposite direction of effects for this eQTL across these cell-types.

In summary, while we feel we have supplemented our previous analysis with additional evidence that finds no other putative causal variants to influence the opposing directions of effect we observe for *FUBP1*, we agree that in lieu of fine-mapping in these cell-types we are limited in our ability to claim these are indeed the same genetic signal with opposing directions of effect. Similarly, eQTL signals could not be fine-mapped in the cell-types with proposed opposing directions of effect for *PSEN2.* Importantly, however, these claims are not central to our argument that regulatory

effects on these genes are influencing disease susceptibility, and so to be cautious we have either removed text that draws the reader's attention to the opposing effects in our variance explained analysis, or updated text to soften these claims in the manuscript:

L367: "*A role for MYC expression in gastrointestinal inflammation is further supported by additional colocalisation between CD risk and an eQTL for FUBP1 (*PP_{h4}*=1.00, Supplementary Figure S11), a regulator of MYC and Wnt components such as DVL1*[65,66]*, and functional evidence from a DSS-induced colitis model in which pharmacological activation of Wnt signalling improved disease severity and restored Myc expression in epithelial cells*[67]"

L453: "*Another notable example was PSEN2, where we observed colocalisation between an eQTL and UC risk (*PP_{h4} *= 0.91) (Supplementary Fig. S12). PSEN2 encodes presenilin 2, a catalytic component of the γ-secretase complex required for Notch receptor cleavage and activation.*"

L461: "*Notably, murine Psen2 knockout models also show susceptibility to colitis*[81]*, in line with our data suggesting that PSEN2 dysregulation may compromise epithelial homeostasis and barrier integrity. Hence, colocalisation of eQTLs across diverse single cell atlases could potentially be used to anticipate context-specific effects of targeted therapies.*"

L561: "*In contrast, at the PSEN2 locus the IBD risk allele was associated with increased expression across epithelial and endothelial cells - likely to enhance Notch activity.*"

4. **(Previous comment #9): We appreciate the authors' rebuttal that the purpose of their study was building a resource for hypothesis generation. Given that big data resources are being published so frequently, it is important for the authors to make a clear case for the utility of theirs with respect to what is currently available. In regard to the benchmarking of this study using data from the Open Targets Genetic Portal, there is still more that can be done to appropriately place this resource in the broader context of IBD genetics research. In lines 200-204 the authors state "Among the 321 IBD loci reported to date, we observed colocalisation with either eQTLs or ieQTLs at 180 loci, implicating 412 distinct effector genes. This includes 74 loci where no effector gene had been previously nominated by colocalisation in the Open Target Genetics portal and 257 novel disease effector genes." From the text it is unclear whether the 257 novel disease effector genes are only at the 74 loci, or if they include novel effector genes at previously resolved loci? Later, on lines 213-214 the authors state "Our eQTLs and ieQTLs also colocalised with 104 of the 137 GWAS loci already reported to colocalise with molecular QTLs in the Open Target Genetics portal. At 78 of these loci, at least one effector gene**

colocalised in both our data and the Open Targets analysis." Can the authors provide an explanation for the 33 loci which they do not replicate in this study? Were those previously reported colocalisations not with eQTL and rather other molecular QTLs? Can the authors provide an explanation for the 26 loci where they nominate different effector genes than previously reported? Were those previous reports from eQTL studies, or other molecular QTLs, and in what tissues?

For clarity, we have addressed the multiple points in this comment separately.

Regarding the specific statement on line 200-204, we apologise for any confusion in our updated text, and appreciate this statement is somewhat ambiguous. We thank the reviewer for pointing this out, and have now updated the text to read:

L201: "*This includes 74 loci where no effector gene had previously been nominated by colocalisation in the Open Targets Genetics portal (Fig. 3a) and 257 novel disease effector genes (156 at loci with previous colocalisation, 101 at newly colocalised loci). Even compared with a sc-eQTL study of peripheral blood mononuclear cells from 1,925 healthy individuals(Cuomo et al. 2025), our study nominates 173 novel effector genes for IBD, including 88 across 49 loci where one is not otherwise nominated* (PP_{h4}*>0.8)...*"

With respect to the question: "**Can the authors provide an explanation for the 33 loci which they do not replicate in this study? Were those previously reported colocalisations not with eQTL and rather other molecular QTLs?**" - After checking the sources of colocalised QTLs from the 33 loci colocalised by Open Targets but not ourselves, 6 (18.2%) are colocalisations with non-expression QTLs in Open Targets. As this represents a 2.5-fold enrichment compared with all loci colocalised by Open Targets (10 out of 137, 7.3%), this suggests the lack of previous eQTL evidence to be a factor contributing to the number of loci for which we do not find any effects.

And finally, for the questions: "**Can the authors provide an explanation for the 26 loci where they nominate different effector genes than previously reported? Were those previous reports from eQTL studies, or other molecular QTLs, and in what tissues?**" - For the 26 loci where different disease effector genes are nominated by Open Targets, 4 (15.4%) of these loci were nominated by only non-expression QTLs in Open Targets, a similar enrichment (2.1-fold) to that outlined in response to the previous question. In addition, at 20 (77%) of these loci we find colocalisation in only contextually restricted cellular annotations (major population or cell-type, but not 'All Cells'). This represents a 1.8-fold enrichment on the total, 43% of the loci we find colocalisation, and suggests that mapping eQTLs at higher resolutions may also contribute to discrepancies between these datasets.

We have now summarised the evidence for the previous two questions in the following text of the revised manuscript:

L228: "*For the 33 and 26 loci for which we either nominate no effector gene, or a different effector gene, colocalisation was more likely to be detected with non-eQTL-based assays only in Open Targets Genetics (2.5-fold and 2.1-fold-enrichment respectively) - indicating assay type could potentially influence the detection and identity of the effector gene being nominated at a minority of loci.*"

Minor:

1. **There are both main and supplementary figures (e.g. Figure 4A-B or Figure 5A-C) where some panels have boxes surrounding the figures, and these should be removed as it is not stylistically consistent.**

   We believe the reviewer is referring to the Manhattan plots that show the regional association of variants for GWAS or eQTL effects, and the x-axis for the GWAS plot as contributing to the 'box'. On review, we agree this is stylistically inconsistent and have therefore removed this x-axis.

2. **In Figure 3A the text overlapping the pie chart should be moved to the side with lines directing it to the relevant fraction because currently some of the letters are not visible.**

   We have now adjusted the sizing and spacing of this figure so there is no longer text exceeding individual sectors of the pie chart. We have therefore left the text within the relevant sectors and updated the colours to match that of the updated Figure 6 in response to minor comment #6.

3. **In Figure 3B the "0.75x" text should be above the "All Cells" point on the plot similar to the text above the points for "Major population" and "Cell type".**

   We appreciate the reviewer's comments on the style of our figures, but are slightly confused by this comment as the text for the 'All Cells' point was previously above (at a greater y-axis position) than the corresponding point in the previous version. After review, we have increased the size of points being plotted, and instead plotted the text below the point for 'All Cells', as we believe this helps convey the negative enrichment of disease effector gene discovery at this resolution.

4. **In Figure 4B the "Fraction of cells in group (%)" text is overlapping and it's not necessary to label each break point. This is also true in Supplementary Figure 15.**

We believe the reviewer is referring to Figure 4A, and have now increased the size of the legend for this figure and all Supplementary Figure 15 panels so that the labels for point size in the legends are no longer overlapping. We have decided to keep each breakpoint to help interpretation.

5. **The legend for Figure 5 needs to include mention of "lead variant presented as diamond" which they have in Figure 4 legend, but it should be in both.**

   The legend of Figure 5 has now been updated to include this detail.

6. **The colors chosen for "novel" and "in OTG" are so close to each other that I'd recommend the authors choose something more distinct. They could remove the y-axis label "mechanism of action" from Figure 6 because it kind of gets lost anyway, and just mention it in the figure legend.**

   We thank the reviewer for pointing out this issue with Figure 6. We have now adjusted the colours to be grey and dark blue which are much more contrasting and match Figure 3a. We have also removed the y-axis label as suggested. The legend for this figure now reads:

   "***Identifying therapeutic repurposing opportunities by colocalisation.*** *Comparison between the disease effector genes identified by colocalisation in IBDverse and the maximum stage of clinical trials therapeutic modifiers of each gene are described to be at in the CHEMBL database (see Methods). Hollow circle = maximum trial phase is 0.5-3, Circle with central dot = drug is approved for use/trial phase 4. Associations for genes that are previously colocalised in Open Targets Genetics (OTG) are grey, genes that colocalise only in IBDverse are dark blue. Drugs are grouped by their broad mechanism of action (y-axis, right) and diseases are grouped into related families (x-axis).*"

Referee #3 (Remarks on code availability):

For the core scripts for eQTL mapping [https://github.com/andersonlab/IBDVerse-sc-eQTL-code [github.com]](https://github.com/andersonlab/IBDVerse-sc-eQTL-code), the README file is missing and I was not able to find scripts to pseudo-bulk single-cell data and run eQTL analyses. The authors should make these scripts clearly available.

We apologise for forgetting to push the updates to the repository. The updated repository is now live with all of the scripts required to run the analyses.

Referee #4 (Remarks to the Author):

I co-reviewed this manuscript with one of the reviewers who provided the listed reports.

Referee #5 (Remarks to the Author):

The authors have addressed my previous concerns.

Referee #5 (Remarks on code availability):

I briefly looked through the provided github and while I didn't run the code it appeared to have sufficient detail to do so.

We would again like to thank reviewers #4/5 for their careful consideration of our manuscript and checking the detail provided in our code.
